# Accurate Diagnostics for *Bovine tuberculosis* Based on High-Throughput Sequencing

**DOI:** 10.1371/journal.pone.0050147

**Published:** 2012-11-30

**Authors:** Alexander Churbanov, Brook Milligan

**Affiliations:** 1 Beijing Institute of Genomics (BIG), Chinese Academy of Sciences, Beijing, China; 2 Biology Department, New Mexico State University, Las Cruces, New Mexico, United States of America; University of Liverpool, United Kingdom

## Abstract

**Background:**

Bovine tuberculosis (bTB) is an enduring contagious disease of cattle that has caused substantial losses to the global livestock industry. Despite large-scale eradication efforts, bTB continues to persist. Current bTB tests rely on the measurement of immune responses *in vivo* (skin tests), and *in vitro* (bovine interferon-γ release assay). Recent developments are characterized by interrogating the expression of an increasing number of genes that participate in the immune response. Currently used assays have the disadvantages of limited sensitivity and specificity, which may lead to incomplete eradication of bTB. Moreover, bTB that reemerges from wild disease reservoirs requires early and reliable diagnostics to prevent further spread. In this work, we use high-throughput sequencing of the peripheral blood mononuclear cells (PBMCs) transcriptome to identify an extensive panel of genes that participate in the immune response. We also investigate the possibility of developing a reliable bTB classification framework based on RNA-Seq reads.

**Methodology/Principal Findings:**

Pooled PBMC mRNA samples from unaffected calves as well as from those with disease progression of 1 and 2 months were sequenced using the Illumina Genome Analyzer II. More than 90 million reads were splice-aligned against the reference genome, and deposited to the database for further expression analysis and visualization. Using this database, we identified 2,312 genes that were differentially expressed in response to bTB infection (*p*<10^−8^). We achieved a bTB infected status classification accuracy of more than 99% with split-sample validation on newly designed and learned mixtures of expression profiles.

**Conclusions/Significance:**

We demonstrated that bTB can be accurately diagnosed at the early stages of disease progression based on RNA-Seq high-throughput sequencing. The inclusion of multiple genes in the diagnostic panel, combined with the superior sensitivity and broader dynamic range of RNA-Seq, has the potential to improve the accuracy of bTB diagnostics. The computational pipeline used for the project is available from http://code.google.com/p/bovine-tb-prediction.

## Introduction

Bovine tuberculosis (bTB) is an insidious, progressive disease of livestock that has cost the United States livestock industry millions of dollars in losses prior to and since the establishment of a national eradication campaign in 1917 [1]. Despite this large-scale eradication effort, bTB is a reemerging infectious disease in the U.S. It is endemic in select areas of Michigan and recent outbreaks have occurred in Minnesota, California, and New Mexico.

*Mycobacterium bovis*, the causative agent of bovine tuberculosis, creates significant problems for agriculture at both the state and national levels. From a management and animal health perspective, it is essential that infected animals are reliably detected and removed to prevent the spread of the disease. Current diagnostic tests are primarily based on immune responses to crude protein extracts from *M. bovis* (PPDb) injected intradermally. Three days after injection of PPDb, excessive swelling at the injection site indicates that the animal may be infected with *M. bovis*.

The sensitivity (*Se*) and specificity (*Sp*) of the single intradermal test (SIT) depends on a cut-off value, and there is an inverse relationship between test *Se* and *Sp* values [2]. For example, the SIT test *Se* could be as high as 91.2%, with an *Sp* of only 75.5% [3], or the *Se* could be only 63.2%, with the *Sp* as high as 99.0% [4], depending on the cut-off. The *Sp* of the tuberculin skin test can be reduced by exposure to environmental non-tuberculous mycobacteria such as *M. avium* and *M. avium subsp. paratuberculosis* [5]. This reduction in *Sp* is due to immunological cross-reactivity between these species. To increase *Sp* while maintaining reasonable *Se*, animals that test positive to the caudal fold (CF) test are tested 60 days later using the Comparative Cervical Test (CCT). The CCT consists of injecting PPDb and a crude protein derivative from *M. avium* (PPDa) at adjacent sites on the neck. Three days later, the swelling at each injection site is compared. If the inflammation at the PPDb injection site is greater than that at the PPDa site, the animal is considered *M. bovis* infected. Conversely, if the swelling at the PPDa site is greater than that at the PPDb site, the animal is considered clinically negative.

Traditional skin testing requires at least 2 animal handling events, one for PPD injection, and another for the evaluation of the test. The need to hold animals for 72 h is a significant disadvantage of PPD testing [6]. The recognition of cytokines and their role in tuberculosis immunology has led to the development of an in vitro assay for bovine interferon-γ (IFN-γ) production [7], [8]. The Bovigam™ assay detects IFN-γ released in response to PPDb in a whole-blood culture assay [8], [9]. Because the Bovigam™ uses the same antigens as the skin test, it has *Se* similar to the SIT with a slightly lower corresponding *Sp* [2]. The reported *Se* of the use of IFN-γ release as a diagnostic tool was 91.4%, whereas the *Sp* was 86.7% [6]; no significant difference was seen between the reliability of the IFN-γ assay and that of the SIT [2], [10], [11]. Despite the national programs in Brazil, the limited sensitivity and specificity of current tests do not facilitate complete bTB eradication in many countries [2], [12].

Using real-time PCR, it has been reported that the expression of IFN-γ, tumor necrosis factor alpha (TNF-*α*), inducible nitric oxide synthase (iNOS), and interleukin (IL)-4 by peripheral blood mononuclear cells (PBMCs) increased in response to infection, whereas that of IL-10 decreased. PPDb-stimulated PBMCs from animals in the high-pathology (with lesions in the lungs and associated lymph nodes) group expressed more IFN-γ, TNF-*α*, iNOS, and IL-4 mRNA than did those from animals in the low-pathology (only had lesions in the head lymph nodes) group at early time points. PBMC expression of the IL-10 gene decreased faster among animals in the high-pathology group, whereas the expression patterns of T-helper (TH) 1 and TH2 cytokines were different among the animals in the high- and low-pathology groups [13]. The maximal difference in expression occurred within the first month after experimental infection. However, over the next 2 months, the IFN-γ responses between the 2 groups reached similar levels. These data suggest that the outcome of disease may be established early after infection. Similar responses were detected in *M. bovis* infected white-tailed deer [14].

Measuring changes in cell products other than IFN-γ after *in vitro* stimulation can yield useful diagnostic assays. For example, an IL-2 receptor A (IL2RA) enzyme-linked immunosorbent assay (ELISA) exhibited a reported sensitivity of 94% and specificity of 98% [15]. ELISA-based and Griess reaction assays were used to determine that changes in TNF-*α* and iNOS expression in PBMCs exposed to PPDa or PPDb antigen could serve as additional diagnostic indices complementing IFN-γ measurements [16]. The advent of high-throughput functional genomics has facilitated studies based on targeted immunospecific bovine cDNA microarrays to discover changes in the expression levels of hundreds of genes, many of which are cytokines [17]–[19].

Diagnostics specific for *M. bovis* that can reliably detect early infection are critical for the eradication program. In this study, we explore the possibility of using next-generation sequencing from PBMC mRNA for the purpose of diagnosing bTB. By quantifying the host immunological response to infection by comparing the transcriptome of known infected and uninfected individuals, we can enhance our ability to detect *M. bovis* in agriculturally important species and, in the future, in potential wildlife reservoirs.

Whole transcriptome sequencing technology (RNA-Seq) based on second-generation sequencing platforms, such as the Illumina Genome Analyzer II, have revolutionized the field of transcriptomics [20]. Quantitative PCR (qPCR) has confirmed the accuracy of RNA-Seq in quantifying gene expression levels [21]. RNA-Seq analysis of spike-in RNA controls of known concentrations also confirmed the high fidelity of the novel technique [22]. Compared to microarray platforms, RNA-Seq delivers higher sensitivity, accuracy, and a broader dynamic range in a hypothesis-neutral way that can help elucidate and annotate novel transcripts [20], [23], [24]. The results of RNA-Seq are highly reproducible, for both technical and biological replicates [21], [25].

In this case-control study, RNA-Seq reads from PBMCs have been splice-aligned against the Btau 4.0 reference genome. We converted the alignment results and Btau 4.0 genome annotation to the general feature format GFF3 and uploaded the results to a MySQL database connected to the Generic Model (GMOD) Generic Genome Browser (GBrowse). Gene expression levels were measured by counting the number of reads mapped against the NCBI annotated gene loci. Based on the fact that RNA-Seq provides highly sensitive measures of absolute and relative gene expression levels, we constructed a probabilistic model for bTB diagnosis. We demonstrated that reliable classification of infected animals could be achieved using only 7,500 reads for each sample.

## Results

### PBMC transcriptome sequencing result

The following numbers of reads were obtained for each pooled transcriptome sample with the Illumina Genome Analyzer II and mapped against the Btau 4.0 reference genome as shown in Table 1. We applied the Fisher exact test to get the list of loci with significant expression changes in response to bTB as presented in Supporting Information S4. We also applied the Fisher exact test to obtain the list of annotated exons with significant coverage changes in response to bTB as presented in Supporting Information S5, which might indicate alternative exon inclusion levels.

**Table 1:** Number of reads.

Sample name	Number of reads	Number of reads mapped
		against reference genome
TCT1	10,421,654	7,616,528 (73.08%)
TCT2	29,316,410	18,324,561 (62.51%)
TCT3	13,881,201	10,143,019 (73.07%)
TCT4	14,512,900	10,645,842 (73.35%)
TCT5	14,606,602	10,627,915 (72.76%)
TCT6	15,189,216	11,071,421 (72.89%)

Number of reads from different pooled transcriptome samples and the total number of reads mapped against the Btau 4.0 reference genome.

The number of reads mapped against informative loci listed in Supporting Information S3 are shown in Table 2. As mentioned in the section *ssec:classification results*, our experiments demonstrated that reliable classification could be achieved with 100 or more reads that map against informative loci. Table 2 shows that the fraction of RNA-Seq reads mapped against the informative loci is approximately 1.35%, which translates to 7,500 RNA-Seq reads per sample that are necessary for reliable classification.

**Table 2:** Number of mapped reads.

Sample name	Number of reads mapped	Total number	Fraction
	to informative loci	of reads	
TCT1	78,434	7,616,528	1.03%
TCT3	137,912	10,143,019	1.36%
TCT5	142,741	10,627,915	1.34%

Number of reads mapped against informative loci.

### Classification results

**Figure 1:**
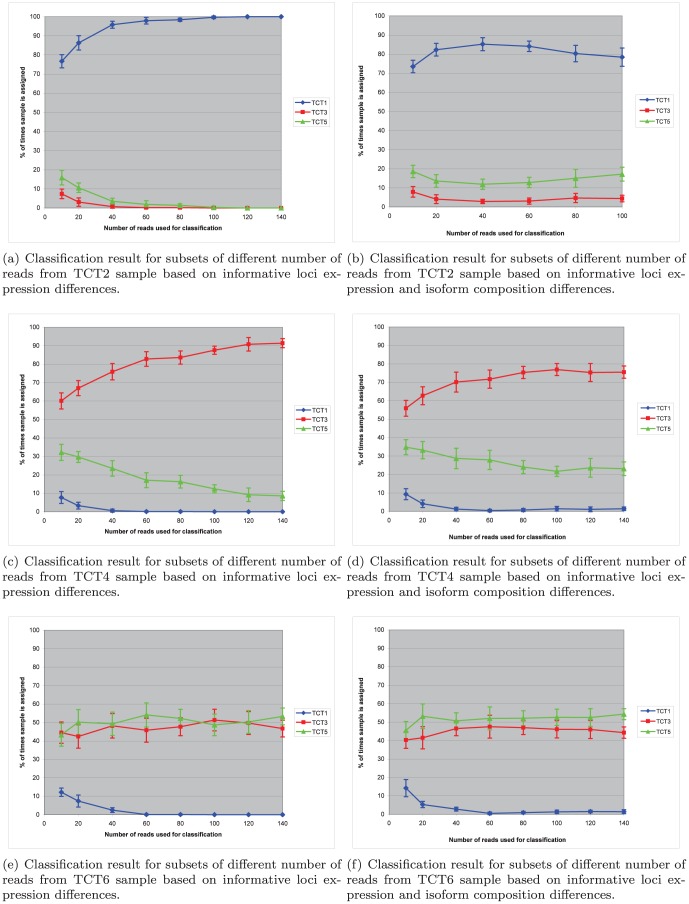
Classification performance. Classification performance for subsets of RNA-Seq reads from samples corresponding to various bTB post infection periods against control (TCT1), one month progression (TCT3) and two months progression (TCT5) trained profiles.

We used pools TCT1, TCT3, and TCT5 to train probabilistic profiles as described in the subsection *Samples classification*. Samples TCT2, TCT4, and TCT6 were used to estimate classification performance, where we used reads mapping against the informative loci mentioned in Supporting Information S3. From the reads that are known to map to informative loci, we randomly sampled groups of sizes 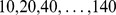 and conducted maximum a posteriori (MAP) classification according to the formulas mentioned in the subsection *Samples classification*. In each category, we formed 10 groups, each containing 100 read sets of sizes in the range 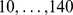 and reported the means and standard deviations of the classification accuracy in these groups. The results of these classifications are shown in Figures 1(a), 1(c) and 1(e).

Figure 1 compares the performance of 2 classification methods. One method aligns the reads against the profiles to calculate forward probability as discussed in the section *Samples classification*. The performance of the alignment-based method is represented in Figures 1(b), 1(d), and 1(f). Another method, based on a much simpler technique, assigns a constant logarithm of probability to all the reads that map against informative loci, listed in Supporting Information S3, according to the genomic short-read nucleotide alignment program (gsNap). This type of classification uses only gene expression mixture proportions for sample classification, as shown in equation (1). This simpler technique results in improved performance, as can be seen in Figures 1(a), 1(c), and 1(e).

### Significant gene expression changes

**Figure 2:**
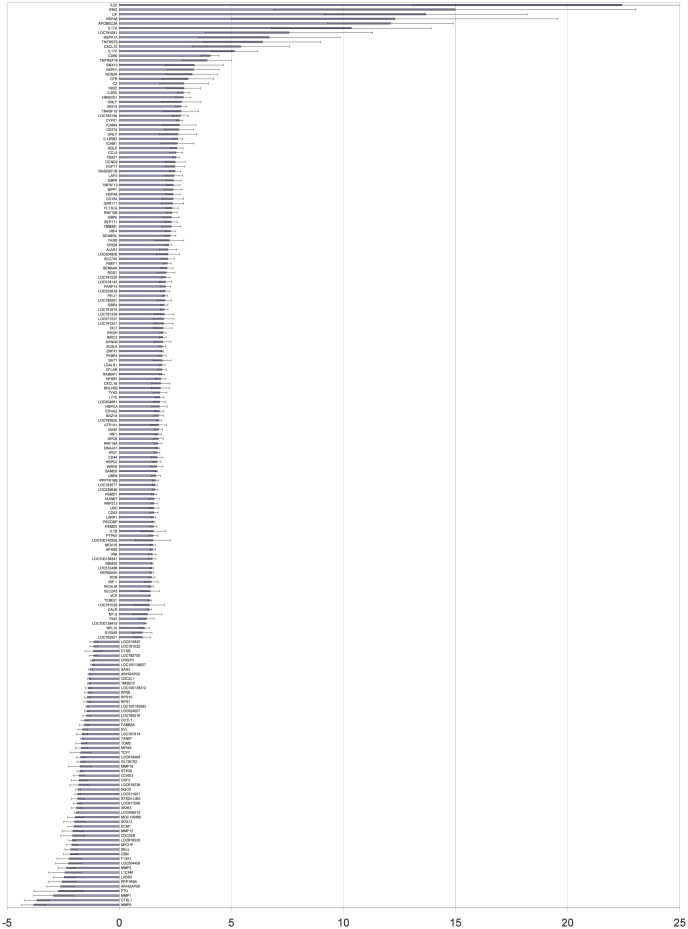
Gene expression changes.

**Figure 3:**
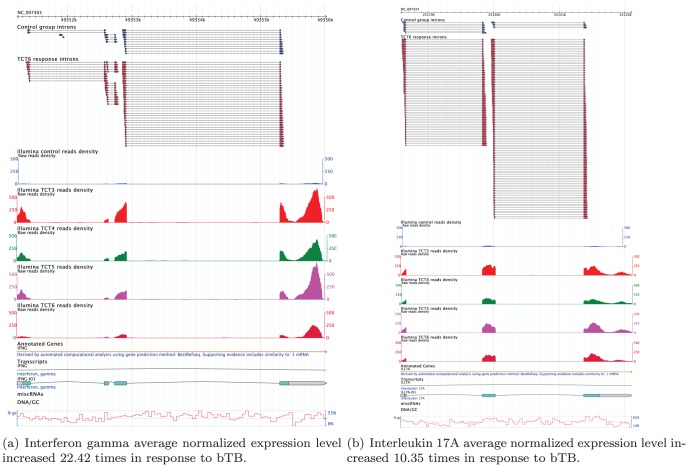
Example of IFN-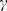 and IL17A unnormalized gene expression changes in response to bTB. On these GBrowse views we show coverage for mapped RNA-Seq reads along with Illumina short reads spanning across introns, i.e. cDNA reads that that partially map to two different exons thus anchoring the exonic boundaries. Here the control reads are the reads from TCT1 pool.

A heat map of statistically significant gene changes (*p*<10^−30^), along with their product names, is provided in Supporting Information S2. A histogram of the expression changes for these loci with error bars is provided in Figure 2. The locations and heat map of exons with statistically significant inclusion discrepancies (*p*<10^−8^) relative to the expression changes of the containing gene locus are provided in Supporting Information S5. Examples of the expression changes of the cytokines IFN-γ and IL-17 in response to bTB, as displayed in GBrowse, are shown in Figure 3.

## Materials and Methods

### RNA-Seq protocol

Seven Holstein calves were obtained from a TB-free herd and housed at the National Animal Disease Center in a biosafety level 3 facility. All animals were housed and cared for in accordance with institutional policies, and procedures were approved by the Institutional Animal Care and Use Committee. The calves received *M. bovis* strain 95-1315 by aerosol at 6 months of age, as described previously [16].

Blood was collected prior to infection, and at 1 month and 2 months post-infection. PBMCs were isolated after stimulation with PPD for 16 hours and the RNA isolated as previously described [14]. The quality of the RNA was tested using an Agilent 2100 Bioanalyzer using the Agilent RNA 6000 Nano Kit according to the manufacturer's instructions. All RNA samples had a RNA integrity number (RIN) value greater than 7.0. Samples (3.3 µg of RNA) from each animal were randomly assigned to 1 of 2 pools at the 0-, 1-, and 2-month timepoints of disease progression. Two RNA pools were generated for each time point, each containing randomly assigned RNA samples from 3 animals. RNA pools from uninfected animals were designated as TCT1 and TCT2, those from the 1-month progression animals were designated as TCT3 and TCT4, and those from the 2-month progression animals were designated as TCT5 and TCT6. Pooled samples were sent to the Iowa State DNA Facility for library preparation and sequencing (75 base run) on the Illumina Genome Analyzer II (one pooled sample per channel).

### Processing the mapped samples

The resulting cDNA reads were splice-aligned against the reference genome Btau 4.0, listed in Supporting Information S6, using the gsNap [26], [27] program. The gsNap tool has been cited [28] as one of the most accurate programs for RNA-Seq reads alignment in a splicing-aware fashion. The alignment results were parsed and deposited into a custom-designed MySQL database and then converted to GFF3 format. The Genbank files were parsed using a BioJava [29]-based parser. All the GFF3 files were uploaded to a MySQL database connected to GBrowse. We used a 2×2 Fisher test to compare the number of reads that map against a gene locus, as annotated in the Btau 4.0 reference genome, to the number of reads mapped against the chromosome containing the locus minus the number of reads that map to the locus in a case-control experiment. We also estimated patterns of differential inclusion of exons by comparing the number of reads that map against an exon, as annotated in Btau 4.0 reference genome, to the number of reads that map against the containing locus minus the number of reads mapped against the exon in case-control experiments. In our experiments, we reported statistically significant differences in gene expression and exon inclusion patterns at significance levels of 0.01 or less in the following tests: 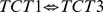, 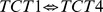, 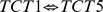, and 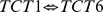. The probability that all 4 tests are significant is 10^−8^.

We show expression changes for genes with significance levels 1×10^−30^ using the heat map built using Bioconductor http://www.bioconductor.org/. The heat map dendrogram shown in Supporting Information S2, was used to identify genes in the top 3 clusters as the most informative classification loci, as listed in Supporting Information S3. These clusters group the most closely related expression profiles having the shortest dendrogram branches. Attempts to use loci in outgroups of these clusters results in suboptimal classification performance in our experiments.

### Samples classification

**Figure 4:**
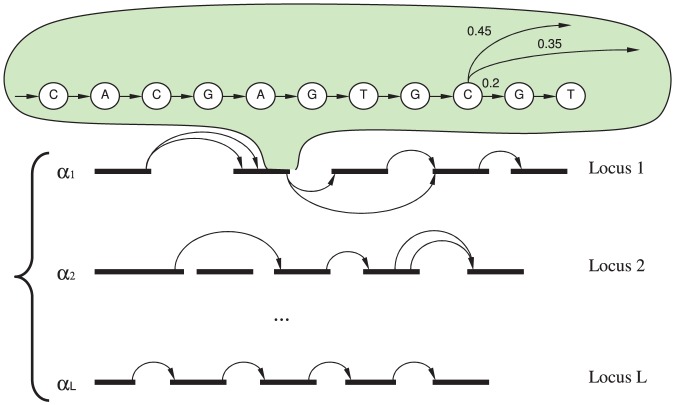
Symbolic representation of classification profiles mixture. Here the mixture components 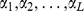 indicate the fraction of all hits mapping against loci used to build a profile. Transition frequencies across introns match the Illumina coverage density at each particular splice site.

In this work, we introduce a hierarchical mixture model for the classification of transcriptomes based on individual RNA-Seq reads. In our model, differential isoform expression patterns can be modeled with various probabilities of exonic isoforms, as shown in Figure 4. The mixture of profiles for different conditions, as seen in Figure 4, form a hierarchical model based on which we generate a MAP classification based on equation (1).

The hidden Markov model (HMM) is a widely accepted stochastic modeling tool [30] used in various domains, such as speech recognition [31] and bioinformatics [32]. HMM is a stochastic finite state machine where each transition between hidden states culminates in the emission of a symbol. The HMM can be represented as a directed graph with *N* states where each state can emit either a discrete character or a continuous value drawn from a probability density function (PDF). In order to describe the HMM, we need the following parameters:

- Set of states, we label individual states as 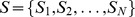, and denote the state visited at time 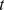 as 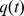,
- Set of PDFs 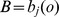 from where emission is drawn 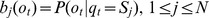. where 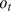 is observation at time moment 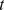 from the sequence of observations 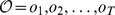.
- The state-transmission probability matrix 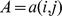, where 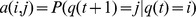,
- The initial state distribution vector 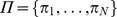.

The set of parameters 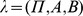 completely specifies the HMM.

Here we adopt the notation from [33]. We need to calculate the expected probability of being at a certain state at a certain moment in time using a forward-backward procedure.

#### Forward procedure

By definition 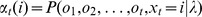 is calculated the following way

1. Initially 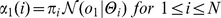,

2. 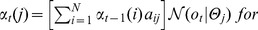
t = 2,3,…,T and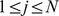,

3. Finally 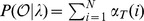 is the sequence *likelihood* according to the model.

Let us consider a set of 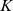 sequences 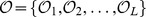 to find the likelihood of the mixture shown in Figure 4.

We calculate the likelihoods matrix as follows:
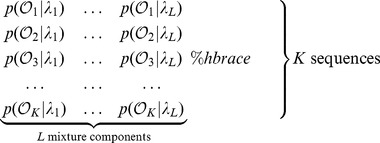



Let us define mixture parameters as 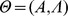 where 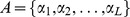 and 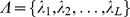


Mixture likelihood is then
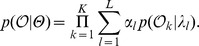



We use Bayes rule to find the posterior probability (responsibility) of a mixture component as shown in Figure 4 with parameters 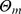 and emission sequences 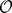 where 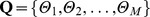


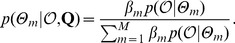
(1)


## Discussion

### Evidence of immunomodulating response

In this study, we demonstrated that RNA-Seq can be used for the early diagnosis of bTB. We identified an extensive panel of genes undergoing differential expression changes in response to infection, as shown in Figure 2. Many of the genes that show increased expression are cytokines, the immune response modulators. We observed significant expression changes in cytokines, including interleukins (IL-22, IL17A, IL17F, IL1A, IL1B) and interferons (IFNG).

According to a study by Meade *et.al.* [18] based on an immune microarray, 378 genes were differentially expressed at the level 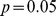 in bTB-infected and control animals. A significant proportion of genes (65%) were expressed at lower levels, among these genes are immune response modulators such as TLR2, TLR4, IFNG, IL-2, IL-4, and the bovine major histocompatibility complex proteins BoLA and BoLA-DRA. Suppression of the key genes modulating the immune response was suggested as one of the mechanisms by which bTB survives host immune defenses. A significant increase in the expression of IFN-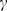, IL-22, CXCL9, CXCL10, GZMA, and IL17A has been reported based on high-density microarray gene expression profiling of the murine immune response against *M. bovis* infection [19], suggesting the use of the elevated expression of these genes as an additional biomarker for bTB diagnosis ante-mortem.

Similar to this study [19], we observed that the expression of key immune response players such as IFNG, IL-22, IL17A, IL17F, NOS2A, TNF, and IL1A increased, as seen in Figures 3 and 2, and in the Supporting Information S4. The majority of the genes (56%) mentioned in Supporting Information S4 show increased expression, as represented in Figure 2. In this study, we confirm the previously observed [13], [16] important roles of IFN-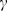 (IFNG), TNF-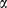 (TNF), and iNOS (NOS2A) in the bTB immune response. As seen in Figure 2, IL2RA gene expression is significantly higher; this confirms diagnostic utility of this gene as described earlier [15]. Expression of IL2RB is also higher, as seen in Supporting Information S4.

The Kyoto Encyclopedia of Genes and Genomes (KEGG) pathway involved in the bovine TB immune response was identified as listed in Supporting Information S1; the majority of immune modulators participating in this pathway were shown in this study to change expression. This study clearly reveals that IL17A responds to bTB infection synergistically with interleukin-1, TNF, iNOS, IFN-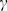, IL2RA and other immune modulators, as shown in Supporting Information S1. We used this coordinated expression as a disease signature for early bTB diagnostics based on RNA-Seq technology. RNA-Seq has been previously reported to be a very sensitive and accurate method of evaluating gene expression with a literally unlimited dynamic range. The inherent stochasticity of RNA-Seq reads is convenient for interrogating the expression of multiple loci.

Further studies are needed to determine if naturally infected animals can also be easily classified using RNA-Seq at the early stages of infection, because experimentally infected animals usually have extremely high immune responses to *M. bovis* antigens. In this study we did not investigate if RNA-Seq is more efficient in distinguishing immune responses from *M. avium* vs. those from *M. bovis*. Further investigation is needed to tell if RNA-Seq has any advantage in 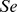 and 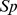 compared to the SIT or the in vitro 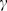-interferon assay. One of the advantages of pooling RNA samples from different animals, as in this study, is the ability to estimate the generalized immune response to bTB infection. However, this approach does not allow estimating differences in the immune reaction that might exist between individual calves, including assessments of the impact of shared sires on the classification results.

### Advantages and limitations of the RNA-Seq classification framework

Figures 1(a),1(c), and 1(e) indicate that for reliable classification, we would need 100 or more reads mapping to informative loci, as seen in Supporting Information S3. The performance of the classification based on the forward algorithm, as discussed in the section *Samples classification*, is represented in Figures 1(b), 1(d), and 1(f). The alignment-based classification is less accurate compared to the simple classification based on gene expression proportions shown in Figures 1(a), 1(c), and 1(e). This is explained by the fact that transitions corresponding to exonic isoform frequencies, as seen in Figure 4, have fluctuations associated with a limited number of reads interrupted by introns. These fluctuations generate some random noise in the loci classification profiles. We detected some gene isoform changes associated with the bTB response in the form of differential inclusion of the exons mentioned in Supporting Information S5. Although we did not use these predicted isoform changes in our bTB classification experiments, they might be useful for future developments in improved bTB diagnostics.

As expected, we had limited resolution between the 1-month and 2-month disease progression animals, as shown in Figures 1(c) and 1(e). In the case of the classification of the first month response to bTB, we have been able to achieve reliable classification, with an accuracy of more than 90%, as seen in Figure 1(c). The accuracy of classification of the 2-month progression samples was approximately 50%, mostly non-discriminatory between the first and second month profiles. However, in all rounds of classification, we had reliable resolution between the infected and uninfected animals. It is possible that extending the panel of the informative loci used for classification can further improve performance.

**Figure 5:**
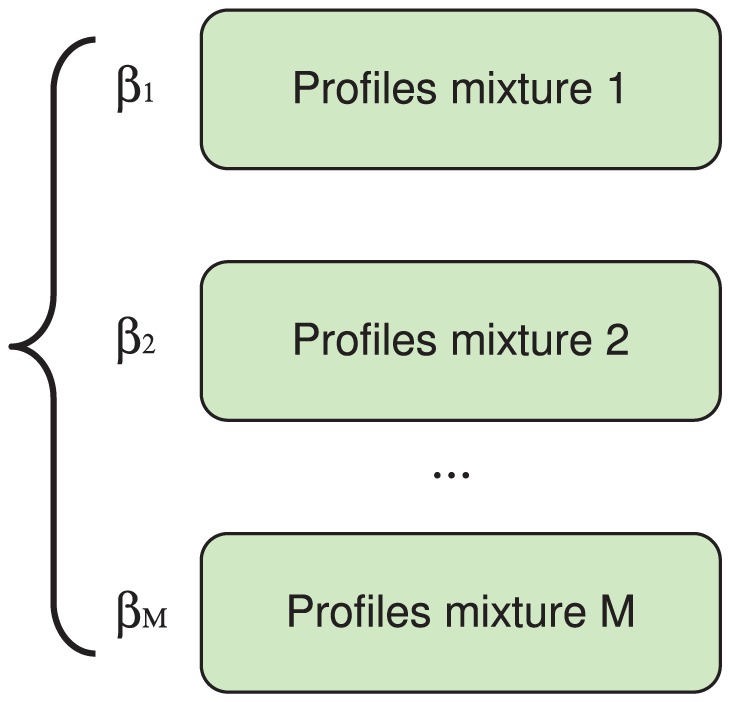
Transcriptome model. Here 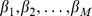 are the prior probabilities of cattle being infected based on previous experience. Each profiles mixture has structure as shown in Figure 4.

The proposed bTB classification framework based on RNA-Seq reads from the PBMC transcriptome has several advantages. It can easily handle the uneven coverage biases of modern sequencers such as Illumina and SOLID [34], and different lengths of underlying loci. The framework can also easily accommodate sequencing errors and known SNPs. The mixture framework is flexible and can model different numbers of genes. The classification process does not require isoform reconstruction, as in some cases it is impossible to infer isoforms from short cDNA reads [35]; it rather presents different isoforms as independent assortments of alternative exonic isoforms. The transcriptome model, as shown in Figure 5, can incorporate prior believes for optimal performance that can be adjusted based on the number of cattle found infected with bTB in a certain area.

## Supporting Information

Supporting Information S1 **Genes participating in immune response.**
(PDF)

Supporting Information S2 **Genes and the product names.**
(PDF)

Supporting Information S3 **Genes used for classification.**
(PDF)

Supporting Information S4 **Genes changing expression.**
(PDF)

Supporting Information S5 **Exons changing inclusion.**
(PDF)

Supporting Information S6 **Reference sequences used.**
(PDF)
