# Supplementary material for: Accurate Diagnostics for Bovine tuberculosis Based on High-Throughput Sequencing
Source: PLoS One. 2012 Nov 30;7(11):e50147. doi: 10.1371/journal.pone.0050147 (PMC3511461; doi:10.1371/journal.pone.0050147)
Supplement: Supporting Information S1 — Genes participating in immune response. (PDF) [file pone.0050147.s001.pdf]

Supporting Information S1

Accurate diagnostics for *Bovine tuberculosis* based on high-throughput sequencing

Alexander Churbanov and Brook Milligan

Genes participating in immune response

According to [1], interleukin (IL)-17 (encoded by the IL17A gene in the Btau 4.0 reference genome) is the founding member of a group of cytokines called the IL-17 family. Interleukin 17 is a cytokine that acts as a potent mediator in delayed-type reactions by increasing chemokine production in various tissues to recruit monocytes and neutrophils to the site of inflammation, similar to interferon gamma (IFN-γ). IL-17 is produced by T helper cells and is induced by IL-23 (IL23A) which results in destructive tissue damage in delayed-type reactions [2]. The members of the IL-17 family function as proinflammatory cytokines that respond to the invasion of the immune system by extracellular pathogens and induce the destruction of the cellular matrix of the pathogen. IL-17 acts synergistically with tumor necrosis factor (TNF) and interleukin-1 [3].

IL-1α and IL-1β are members of the IL-1 family [4, 5]. These proteins are encoded by the IL1A and IL1B genes in the Btau 4.0 cow reference genome. IL-1 possesses a wide spectrum of metabolic, physiological, and hematopoietic activities, and plays a central role in the regulation of immune responses [6, 7, 8].

According to [9], the TNF family refers to a group of cytokines such as TNF-α and TNF-β. In cows, the TNF-α protein is encoded by the TNF gene in the Btau 4.0 reference genome. The primary role of TNF is in the regulation of immune cells. TNF is able to induce apoptotic cell death, induce inflammation, and inhibit tumorigenesis and viral replication [10].

Interferon-gamma (IFN-γ), the only member of the type II interferons, is a dimerized soluble cytokine. In cows, the IFN-γ protein is encoded by the IFNG gene in the Btau 4.0 reference genome. According to RefSeq (`http://www.ncbi.nlm.nih.gov/RefSeq/`), the encoded protein is

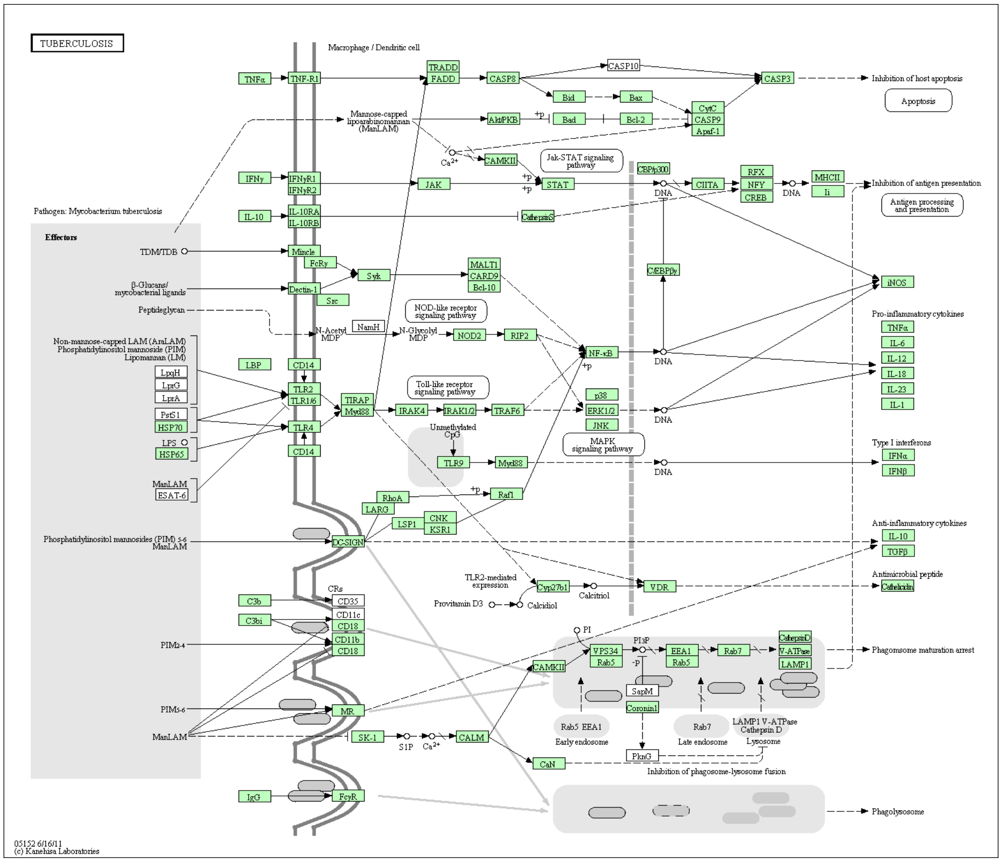

Figure 1: Bovine tuberculosis pathway from KEGG database [16, 17].

a soluble cytokine with antiviral, immunoregulatory, and anti-tumor properties, and is a potent activator of macrophages. The importance of IFN-γ in the immune system stems in part from its ability to inhibit viral replication directly, and most importantly, from its immunostimulatory and immunomodulatory effects [11]. IFN-γ is produced predominantly by natural killer (NK) and natural killer T (NKT) cells as part of the innate immune response, and by CD4 and CD8 cytotoxic T lymphocyte (CTL) effector T cells once antigen-specific immunity develops [12, 13].

The inducible nitric oxide synthase (iNOS) enzyme [14] is encoded by the NOS2A gene in the Btau 4.0 reference genome. According to RefSeq, nitric oxide is a reactive free radical that acts as a biological mediator in several processes, including neurotransmission and antimicrobial and antitumor activities. This gene encodes a nitric oxide synthase that is expressed in the liver and is inducible by a combination of lipopolysaccharide and certain cytokines.

According to RefSeq, the interleukin-2 (IL-2) receptor α (IL2RA) and β (IL2RB) chains, together with the common γ chain (IL2RG), constitute the high-affinity IL-2 receptor [15]. Homodimeric α chains (IL2RA) result in a low-affinity receptor, while homodimeric β (IL2RB) chains produce a

medium-affinity receptor.

Bovine tuberculosis immune response pathway from KEGG database `http://www.genome.jp/kegg-bin/show_pathway?bta05152` is shown in Figure 1.

References

[1] contributors W (2011). Interleukin 17. Wikipedia, The Free Encyclopedia.

[2] Kuby J, Kindt T, Goldsby R, Osborne B (2007) Kuby immunology, San Francisco: W.H. Freeman, chapter 15. p. 396.

[3] Miossec P, Korn T, Kuchroo V (2009) Interleukin-17 and type 17 helper T cells. N Engl J Med 361: 888-898.

[4] Kanehisa M, Goto S, Sato Y, Furumichi M, Tanabe M (2012) KEGG for integration and interpretation of large-scale molecular datasets. Nucleic Acids Research 40: D109–D114.

[5] Kanehisa M, Goto S (2000) KEGG: kyoto encyclopedia of genes and genomes. Nucleic Acids Research 28: 27–30.

[6] March C, Mosley B, Larsen A, Cerretti D, Braedt G, et al. (1985) Cloning, sequence and expression of two distinct human interleukin-1 complementary DNAs. Nature 315: 641-647.

[7] Nicklin M, Weith A, Duff G (1994) A physical map of the region encompassing the human interleukin-1 alpha, interleukin-1 beta, and interleukin-1 receptor antagonist genes. Genomics 19: 382-384.

[8] Bankers-Fulbright J, Kalli K, McKean D (1996) Interleukin-1 signal transduction. Life Sci 59: 61-83.

[9] Dinarello C (1997) Induction of interleukin-1 and interleukin-1 receptor antagonist. Semin Oncol 24: S981-S993.

[10] contributors W (2011). Il1a. Wikipedia, The Free Encyclopedia.

[11] contributors W (2011). Tumor necrosis factors. Wikipedia, The Free Encyclopedia.

[12] contributors W (2011). Tumor necrosis factor-alpha. Wikipedia, The Free Encyclopedia.

[13] Schroder K, Hertzog P, Ravasi T, Hume D (2004) Interferon-gamma: an overview of signals, mechanisms and functions. J Leukoc Biol 75: 163-89.

[14] Schoenborn J, Wilson C (2007) Regulation of interferon-gamma during innate and adaptive immune responses. Adv Immunol 96: 41-101.

[15] contributors W (2011). Interferon-gamma. Wikipedia, The Free Encyclopedia.

[16] Geller D, Lowenstein C, Shapiro R, Nussler A, Di Silvio M, et al. (1993) Molecular cloning and expression of inducible nitric oxide synthase from human hepatocytes. Proc Natl Acad Sci USA 90: 3491-3495.

[17] Waldmann T (1991) The interleukin-2 receptor. J Biol Chem 266: 2681-2684.
