# Supplementary material for: Accurate Diagnostics for Bovine tuberculosis Based on High-Throughput Sequencing
Source: PLoS One. 2012 Nov 30;7(11):e50147. doi: 10.1371/journal.pone.0050147 (PMC3511461; doi:10.1371/journal.pone.0050147)
Supplement: Supporting Information S2 — Genes and the product names. (PDF) [file pone.0050147.s002.pdf]

Supporting Information S2

Accurate diagnostics for *Bovine tuberculosis* based on high-throughput sequencing

Alexander Churbanov and Brook Milligan

Genes and the product names

Heatmap for genes changing expression at the significance level of 1×10^{-30} could be seen in Figure 1.

Table 1: Gene along with the product names.

Gene	Product
ACSL5	Acyl-CoA synthetase long-chain family member 5
AHNAK	AHNAK nucleoprotein, transcript variant 1
ALAS1	Aminolevulinate, delta-, synthase 1
APOBEC3A	Similar to Apobec3G
ARHGAP26	Similar to ARHGAP26 protein
ARHGAP30	Similar to Rho GTPase activating protein 30, transcript variant 1
ATP1A1	ATPase, Na+/K+ transporting, alpha 1 polypeptide
BAZ1A	Similar to Bromodomain adjacent to zinc finger domain protein 1A (Cbp146), transcript variant 2
BHLHB2	Basic helix-loop-helix domain containing, class B, 2
BIRC3	Baculoviral IAP repeat-containing 3
C2	Complement component 2
CALR	Calreticulin
CCL5	Chemokine (C-C motif) ligand 5
CCND2	Cyclin D2
CCND3	Cyclin D3
CD164	CD164 molecule, sialomucin
CD274	Similar to CD274 antigen
CD44	CD44 molecule (Indian blood group)
CD53	CD53 molecule
CD69	CD69 molecule
CDC25B	Similar to cell division cycle 25B, transcript variant 2

Continued on next page...

Table 1 – continued from previous page

Gene	Product
CDC2L1	Cell division cycle 2-like 1 (PITSLRE proteins)
CFB	Complement factor B
CFLAR	CASP8 and FADD-like apoptosis regulator
COTL1	Coactosin-like 1 (Dictyostelium)
CREM	CAMP responsive element modulator
CRISP3	Cysteine-rich secretory protein 3
CSF3	Colony stimulating factor 3 (granulocyte)
CTSB	Cathepsin B
CTSL1	Cathepsin L1
CXCL10	Chemokine (C-X-C motif) ligand 10
CXCL16	Chemokine (C-X-C motif) ligand 16
CYP51	Cytochrome P450, family 51, subfamily A, polypeptide 1
DCT	Dopachrome tautomerase (dopachrome delta-isomerase, tyrosine-related protein 2)
DNAJA1	DnaJ (Hsp40) homolog, subfamily A, member 1
ECM1	Extracellular matrix protein 1
EIF4A2	Eukaryotic translation initiation factor 4A, isoform 2
EVL	Similar to Enah/Vasp-like, transcript variant 1
F13A1	Coagulation factor XIII, A1 polypeptide
FAM62A	Family with sequence similarity 62 (C2 domain containing), member A
FDFT1	Farnesyl-diphosphate farnesyltransferase 1
FKBP4	FK506 binding protein 4, 59kDa
FLT3LG	Fms-related tyrosine kinase 3 ligand
FTO	Fat mass and obesity associated
GBP4	Guanylate binding protein 4
GBP5	Guanylate binding protein 5
GBP6	Similar to guanylate binding protein family, member 6, transcript variant 1
GLTSCR2	Glioma tumor suppressor candidate region gene 2
GNLY	Granulysin
GPC6	Glypican 6
GPR171	G protein-coupled receptor 171
GSN	Gelsolin (amyloidosis, Finnish type)
HMGCS1	3-hydroxy-3-methylglutaryl-coenzyme A synthase 1, transcript variant 1
HSP90AB1	Heat shock 90kDa protein 1, beta
HSPA1A	Heat shock 70kDa protein 1A
HSPA6	Similar to heat shock protein 70
HSPA8	Heat shock 70kDa protein 8
HSPCA	Heat shock 90kD protein 1, alpha
HSPD1	Heat shock 60kDa protein 1 (chaperonin)
HSPH1	Heat shock 105kDa/110kDa protein 1
HUWE1	HECT, UBA and WWE domain containing 1
ICAM1	Intercellular adhesion molecule 1 (CD54), human rhinovirus receptor
ICAM4	Intercellular adhesion molecule 4 (Landsteiner-Wiener blood group)
IFNG	Interferon, gamma
IL12RB2	Interleukin 12 receptor, beta 2
IL17A	Interleukin 17A
IL17F	Similar to interleukin 17F
IL1B	Interleukin 1, beta
IL22	Interleukin 22
IL2RA	Interleukin 2 receptor, alpha
INDO	Indoleamine-pyrrole 2,3 dioxygenase
IPO7	Similar to Importin-7 (Imp7) (Ran-binding protein 7) (RanBP7)

Continued on next page...

Table 1 – continued from previous page

Gene	Product
IRF1	Hypothetical LOC789216
IRF4	Similar to interferon regulatory factor 4, transcript variant 2
ISG15	ISG15 ubiquitin-like modifier
JSP.1	MHC Class I JSP.1
L1CAM	Similar to L1 cell adhesion molecule, transcript variant 1
LAP3	Leucine aminopeptidase 3
LARP1	Similar to la related protein
LASS3	LAG1 homolog, ceramide synthase 3
LGALS1	Lectin, galactoside-binding, soluble, 1 (galectin 1)
LIF	Leukemia inhibitory factor (cholinergic differentiation factor)
LOC100138312	Similar to Long-chain-fatty-acid–CoA ligase 3 (Long-chain acyl-CoA synthetase 3) (LACS 3)
LOC100138341	Hypothetical protein LOC100138341
LOC100138627	Hypothetical protein LOC100138627
LOC100139419	Similar to eukaryotic translation elongation factor 1 alpha 1, transcript variant 1
LOC100140226	Similar to zinc finger protein 813
LOC100140583	Similar to OVARIAN/Breast septin gamma
LOC504406	Similar to hydrocephalus inducing
LOC504806	Similar to hCG30005
LOC504861	Similar to cationic amino acid transporter 5
LOC506412	Serum amyloid A-like
LOC511531	Similar to guanylate binding protein 1, transcript variant 2
LOC511901	Similar to H1 histone family, member X
LOC512486	Similar to Interferon-induced guanylate-binding protein 1 (GTP-binding protein 1) (Guanine nucleotide-binding protein 1) (HuGBP-1), transcript variant 3
LOC513842	Similar to Ferritin, heavy polypeptide 1, transcript variant 1
LOC514143	Hypothetical LOC514143
LOC524507	Similar to ribosomal protein S19
LOC533818	Similar to guanylate binding protein 4
LOC535649	Similar to Formin-binding protein 4 (Formin-binding protein 30)
LOC616035	Similar to serum amyloid A1 preproprotein
LOC617566	Similar to bone morphogenetic protein 6
LOC618238	Similar to mammary serum amyloid A3.2
LOC618464	Similar to 60S ribosomal protein L35
LOC781022	Similar to ferritin H subunit
LOC781039	Similar to pericardine
LOC781081	Similar to heat-shock 70-kilodalton protein 1A
LOC781225	Similar to guanylate binding protein 4
LOC781251	Similar to 90-kDa heat shock protein alpha
LOC781339	Similar to 90-kDa heat shock protein alpha
LOC781675	Similar to guanylate binding protein 4
LOC782705	Similar to Ferritin, heavy polypeptide 1
LOC782921	Similar to ferritin L subunit
LOC782951	Similar to DnaJ (Hsp40) homolog, subfamily A, member 1
LOC783184	Similar to HMGCS1 protein
LOC783577	Similar to 90-kDa heat shock protein beta
LOC783920	Similar to mCG1046517
LOC785516	Similar to mCG10725, transcript variant 1
LOC787914	Similar to ribosomal protein S6-like
LY75	Lymphocyte antigen 75

Continued on next page...

Table 1 – continued from previous page

Gene	Product
MFNG	MFNG O-fucosylpeptide 3-beta-N-acetylglucosaminyltransferase
MGC128480	Hypothetical gene LOC539635
MMP1	Matrix metallopeptidase 1 (interstitial collagenase)
MMP12	Similar to matrix metallopeptidase 12
MMP19	Matrix metallopeptidase 19
MMP3	Matrix metallopeptidase 3 (stromelysin 1, progelatinase)
MMP9	Matrix metallopeptidase 9 (gelatinase B, 92kDa gelatinase, 92kDa type IV collagenase)
MOV10	Mov10, Moloney leukemia virus 10, homolog (mouse)
MPP7	Membrane protein, palmitoylated 7 (MAGUK p55 subfamily member 7)
MT-2	Metallothionein-2
MYO1F	Similar to myosin IF
NFKB1	Nuclear factor of kappa light polypeptide gene enhancer in B-cells 1 (p105)
NFKB2	Nuclear factor of kappa light polypeptide gene enhancer in B-cells 2 (p49/p100)
NOS2A	Nitric oxide synthase 2A (inducible, hepatocytes)
NQO2	NAD(P)H dehydrogenase, quinone 2
NUB1	Negative regulator of ubiquitin-like proteins 1
PAI2	Plasminogen activator inhibitor-2, transcript variant 1
PARP14	Poly (ADP-ribose) polymerase family, member 14
PBEF1	Pre-B-cell colony enhancing factor 1, transcript variant 4
PELI1	Pellino homolog 1 (Drosophila)
PICALM	Phosphatidylinositol binding clathrin assembly protein
PPP1R16B	Protein phosphatase 1, regulatory (inhibitor) subunit 16B
PPP1R9A	Protein phosphatase 1, regulatory (inhibitor) subunit 9A
PSCDBP	Pleckstrin homology, Sec7 and coiled-coil domains, binding protein
PSMD1	Proteasome (prosome, macropain) 26S subunit, non-ATPase, 1
PSMD2	Proteasome (prosome, macropain) 26S subunit, non-ATPase, 2
PTPN1	Protein tyrosine phosphatase, non-receptor type 1
RAB6IP1	Similar to Rab6 interacting protein 1
RASGEF1B	RasGEF domain family, member 1B
RBM39	RNA binding motif protein 39, transcript variant 13
RGS1	Regulator of G-protein signaling 1, transcript variant 1
RHOH	Ras homolog gene family, member H
RNF19A	Similar to ring finger protein 19
RNF19B	Similar to IBR domain containing 3
RNF213	Similar to mCG142721
RPL10	Similar to ribosomal protein L10
RPS10	Ribosomal protein S10
RPS6	Similar to ribosomal protein S6-like
RPS7	Ribosomal protein S7
S100A9	S100 calcium binding protein A9
SAA3	Serum amyloid A 3
SAMD9	Sterile alpha motif domain containing 9
SAT1	Spermidine/spermine N1-acetyltransferase 1
SC4MOL	Sterol-C4-methyl oxidase-like
SELL	Selectin L (lymphocyte adhesion molecule 1)
SEMA4A	Sema domain, immunoglobulin domain (Ig), transmembrane domain (TM) and short cytoplasmic domain, (semaphorin) 4A
SEPT11	Septin 11
SLC2A3	Solute carrier family 2 (facilitated glucose transporter), member 3
SLC7A5	Solute carrier family 7 (cationic amino acid transporter, y+ system), member 5

Continued on next page...

Table 1 – continued from previous page

Gene	Product
SNX10	Sorting nexin 10
SON	SON DNA binding protein
SOX13	Similar to type 1 diabetes autoantigen ICA12
SQLE	Squalene epoxidase
STK24-LIKE	Serine/threonine kinase 24 (STE20 homolog, yeast)-like
STK38	Serine/threonine kinase 38
TARS	Threonyl-tRNA synthetase
TBX21	Similar to T-box transcription factor TBX21 (T-box protein 21) (Transcription factor TBLYM) (T-cell-specific T-box transcription factor T-bet)
TCF7	Transcription factor 7 (T-cell specific, HMG-box)
TCIRG1	T-cell, immune regulator 1, ATPase, H+ transporting, lysosomal V0 subunit A3
TGM3	Transglutaminase 3 (E polypeptide, protein-glutamine-gamma-glutamyltransferase)
TM4SF19	Similar to transmembrane 4 L six family member 19
TMBIM1	Transmembrane BAX inhibitor motif containing 1
TMSB10	Thymosin, beta 10
TNFRSF18	Similar to tumor necrosis factor receptor superfamily, member 18
TNFRSF9	Tumor necrosis factor receptor superfamily, member 9
TNFSF10	Similar to TNF-related apoptosis-inducing ligand
TXNIP	Thioredoxin interacting protein
TYK2	Tyrosine kinase 2
UBC	Ubiquitin C, transcript variant 12
UBR4	Similar to E3 ubiquitin-protein ligase UBR4 (N-recognin-4) (Zinc finger UBR1-type protein 1) (Retinoblastoma-associated factor of 600 kDa) (600 kDa retinoblastoma protein-associated factor) (RBAF600) (p600), transcript variant 1
VCP	Valosin-containing protein
VIM	Vimentin
WARS	Tryptophanyl-tRNA synthetase
WDR3	Similar to WD repeat-containing protein 3
ZNFX1	Similar to NFX1-type zinc finger-containing protein 1

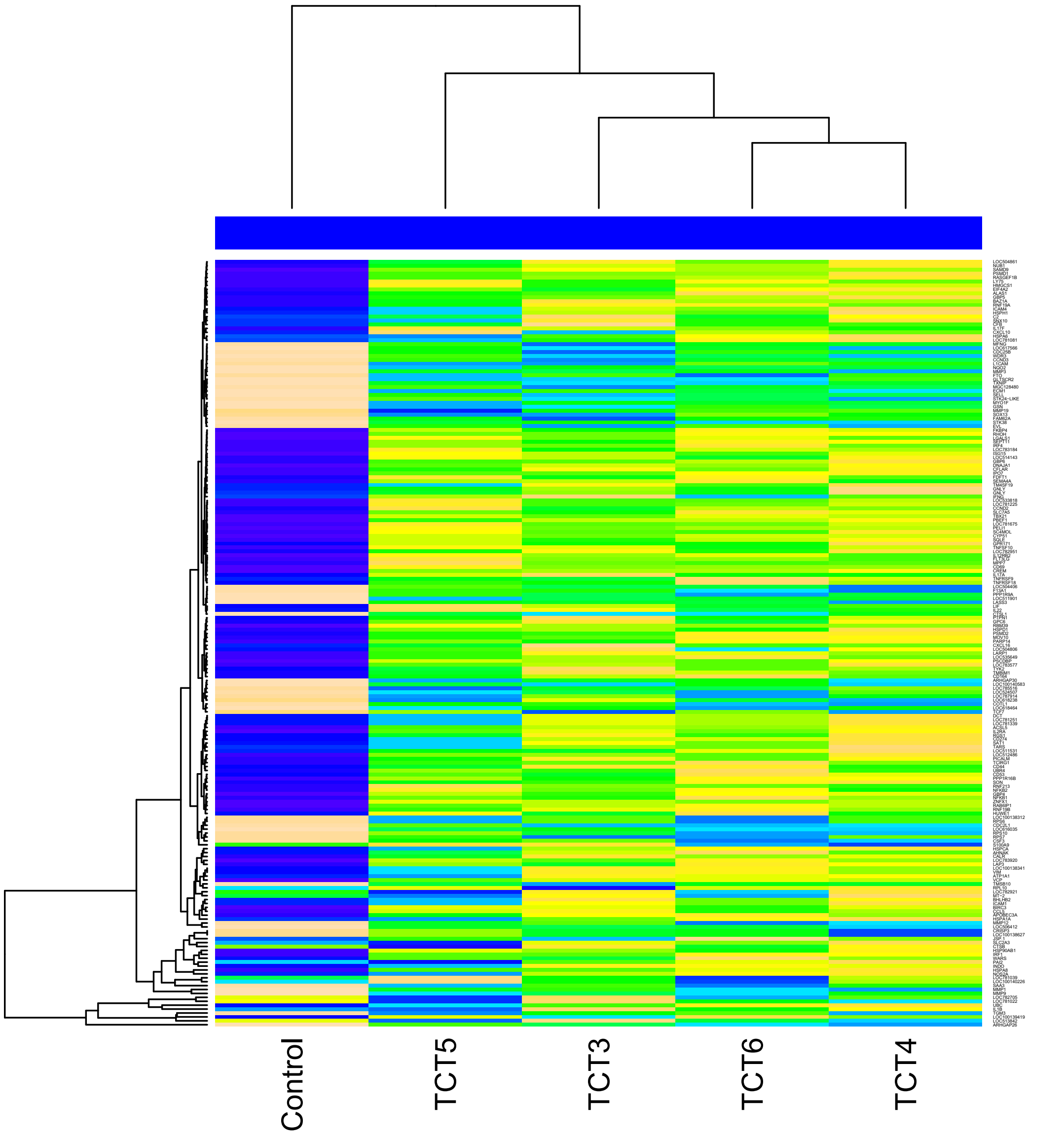

Figure 1: **Heatmap of gene expression changes.** Heat map along the dendrograms of gene expressions. Blue color indicates genes relative underexpressed and sandstone color indicates genes relatively overexpressed.
