# Supplementary material for: Accurate Diagnostics for Bovine tuberculosis Based on High-Throughput Sequencing
Source: PLoS One. 2012 Nov 30;7(11):e50147. doi: 10.1371/journal.pone.0050147 (PMC3511461; doi:10.1371/journal.pone.0050147)
Supplement: Supporting Information S3 — Genes used for classification. (PDF) [file pone.0050147.s003.pdf]

Supporting Information S3

Accurate diagnostics for *Bovine tuberculosis* based on high-throughput sequencing

Alexander Churbanov and Brook Milligan

Genes used for classification

We used three upper clusters of genes in the dendrogram for classification.

Table 1: List of genes used for classification.

Gene	Location
ALAS1	NC_007320:49685455..49699524
BAZ1A	NC_007319:45912454..46003648
C2	NC_007324:27213701..27225299
CCND2	NC_007303:112629798..112652687
CCND3	NC_007324:16321575..16328397
CD69	NC_007303:107783338..107790440
CDC25B	NC_007311:51954757..51964354
CFB	NC_007324:27207241..27213267
CFLAR	NC_007300:94017519..94051080
CREM	NC_007311:17474894..17541383
CXCL10	NC_007304:94129095..94131447
CYP51	NC_007302:9698100..9714943
DNAJA1	NC_007306:78911556..78921312
ECM1	NC_007301:21721706..21727111
EIF4A2	NC_007299:82284446..82290755
EVL	NC_007319:65129949..65192725
FAM62A	NC_007303:61744941..61759003
FDFT1	NC_007306:7470094..7496546

Continued on next page...

Table 1 – continued from previous page

Gene	Location
FKBP4	NC_007303:113816230..113824044
FLT3LG	NC_007316:55881354..55890835
FTO	NC_007316:21434346..21904695
GBP5	NC_007301:57497535..57514016
GBP6	NC_007301:57264867..57290616
GLTSCR2	NC_007316:54505640..54514304
GNLY	NC_007309:51004917..51007329
GNLY	NC_007309:50960680..50968719
GPR171	NC_007299:118471626..118478167
GSN	NC_007306:116103605..116131438
HMGCS1	NC_007318:33424018..33449753
HSPA6	NC_007301:8748711..8750920
HSPH1	NC_007310:29596281..29619754
ICAM4	NC_007305:13286426..13288828
IFNG	NC_007303:49351209..49356033
IL12RB2	NC_007301:83639585..83713938
IL17A	NC_007324:25118586..25122111
IL17F	NC_007324:25158307..25165851
IPO7	NC_007313:42206076..42251721
IRF4	NC_007324:53251659..53264380
ISG15	NC_007314:48718794..48719821
L1CAM	NC_007331:23379678..23393995
LGALS1	NC_007303:116532588..116535544
LOC504861	NC_007316:60944626..60950509
LOC514143	NC_007301:57607852..57631551
LOC533818	NC_007301:58115305..58135887
LOC617566	NC_007324:48404191..48567050
LOC781081	NC_007324:27325486..27326188
LOC781225	NC_007301:57896817..57916331
LOC781675	NC_007301:58148065..58164989
LOC782951	NC_007313:18562896..18564373
LOC783184	NC_007318:33462349..33481091
LY75	NC_007300:37616016..37922391
MFNG	NC_007303:81642453..81658180
MGC128480	NC_007324:33026253..33108031
MMP19	NC_007303:61991908..61998019
MMP3	NC_007313:4728091..4734670
MPP7	NC_007311:36244688..36467676
MYO1F	NC_007305:15580048..15622883
NQO2	NC_007324:51580497..51591400
NUB1	NC_007302:118184860..118224078
PBEF1	NC_007302:49240668..49281361
	Continued on next page...

Table 1 – continued from previous page

Gene	Location
PELI1	NC_007309:64300160..64356728
PSMD1	NC_007300:123040871..123122125
RASGEF1B	NC_007304:99642884..99681165
RHOH	NC_007304:61579571..61627674
RNF19A	NC_007312:62125690..62198945
SAMD9	NC_007302:10705119..10725602
SC4MOL	NC_007315:493451..510407
SELL	NC_007314:34766862..34792450
SEMA4A	NC_007301:15943413..15962532
SEPT11	NC_007304:95019677..95120079
SLC7A5	NC_007316:12563370..12592282
SNX10	NC_007302:72320292..72350997
SOX13	NC_007314:897405..911101
SQLE	NC_007312:14605537..14627772
STK24-LIKE	NC_007310:76824126..76860443
STK38	NC_007324:10699037..10740337
TBX21	NC_007317:39957707..39969532
TM4SF19	NC_007299:72093751..72109873
TNFRSF18	NC_007314:48565346..48568099
TNFRSF9	NC_007314:42581703..42600709
TNFSF10	NC_007299:97101994..97118087
TXNIP	NC_007301:23256754..23259469
WDR3	NC_007301:27529412..27568528
