# Supplementary material for: Accurate Diagnostics for Bovine tuberculosis Based on High-Throughput Sequencing
Source: PLoS One. 2012 Nov 30;7(11):e50147. doi: 10.1371/journal.pone.0050147 (PMC3511461; doi:10.1371/journal.pone.0050147)
Supplement: Supporting Information S4 — Genes changing expression. (PDF) [file pone.0050147.s004.pdf]

Supporting Information S4

Accurate diagnostics for *Bovine tuberculosis* based on high-throughput sequencing

Alexander Churbanov and Brook Milligan

Genes changing expression

Table 1: List of genes changing expression with statistical significance equal or less than 0.01 with the Fischer test comparing expression levels of TCT1 with TCT3, TCT4, TCT5 and TCT6. Number of Illumina reads mapped to the gene loci normalized by the number of reads mapped against the containing chromosome.

Gene	Location	TCT1	TCT3	TCT4	TCT5	TCT6
A2BP1	NC_007326:6757055..7522724	247.00	183.84	101.19	173.98	124.64
AACS	NC_007315:53550932..53603916	249.00	314.09	353.70	464.84	427.88
AARS	NC_007316:1142685..1163961	1,092.00	1,403.88	1,336.84	1,415.84	1,515.91
ABCA1	NC_007306:99370237..99496019	1,834.00	2,114.52	1,363.81	1,310.41	1,336.86
ABCA3	NC_007326:2303746..2335485	1,258.00	1,560.15	1,011.10	891.10	963.22
ABCC3	NC_007317:37220616..37268457	120.00	63.23	69.38	73.06	78.48
ABCF3	NC_007299:84835494..84843338	479.00	584.57	765.05	738.60	724.52
ABCG1	NC_007299:145352437..145416305	93.00	28.29	53.89	21.52	27.29
ABHD13	NC_007310:81650476..81663830	126.00	210.23	197.72	169.33	267.46
ABHD14A	NC_007320:49945052..49951545	59.00	27.77	27.59	21.82	16.14
ABHD5	NC_007320:16004811..16046288	64.00	96.38	121.08	107.72	102.42
ABI1	NC_007311:17077374..17160291	612.00	957.71	934.35	959.35	878.41
ABI3	NC_007317:38557536..38566767	207.00	146.32	114.27	144.06	140.50
ABL1	NC_007309:104723923..104867018	758.00	644.75	505.97	566.74	627.21
ABR	NC_007317:21829660..21887283	2,050.00	1,796.98	1,512.04	1,445.39	1,475.25
ABTB2	NC_007313:64458475..64645633	285.00	578.69	541.67	483.26	526.91
ACAA2	NC_007325:51171526..51204194	359.00	486.23	511.26	455.46	525.23
ACCN1	NC_007317:16896935..17007203	126.00	72.06	42.85	84.09	70.25
ACO1	NC_007306:11700824..11763811	817.00	1,269.17	964.77	688.19	965.47
ACP5	NC_007305:14363311..14366902	758.00	331.09	421.02	453.61	429.17
ACSL4	NC_007331:35810908..35856002	945.00	1,486.64	1,560.13	1,242.31	1,483.74
ACSL5	NC_007327:33318726..33369839	2,575.00	5,026.67	5,423.18	4,476.56	4,868.27
ACSL6	NC_007305:21167803..21225082	2,195.00	5,382.35	4,782.79	2,501.37	3,520.28

Continued on next page...

Table 1 – continued from previous page

Gene	Location	TCT1	TCT3	TCT4	TCT5	TCT6
ACSM3	NC_007326:19581626..19690541	397.00	623.22	637.72	535.83	537.35
ACSS1	NC_007311:42772503..42815875	729.00	1,140.47	874.94	843.51	1,016.85
ACTB	NC_007326:40635163..40638584	45,681.00	36,140.60	43,635.70	50,014.87	50,206.57
ACTG1	NC_007317:52748526..52751375	36,791.00	24,121.05	29,451.10	38,158.31	35,459.23
ACTR3	NC_007300:68805338..68852297	4,466.00	5,155.03	5,605.42	5,171.98	5,061.45
ADAM17	NC_007309:90693086..90737685	730.00	1,188.20	1,165.22	1,110.76	1,260.86
ADAM19	NC_007305:68882927..68971338	431.00	638.48	564.82	521.15	786.04
ADAM8	NC_007327:51582540..51595073	2,720.00	2,315.31	2,293.31	1,805.58	2,460.15
ADAM9	NC_007328:36448783..36529046	843.00	1,149.36	1,281.48	545.02	1,241.93
ADAMTS17	NC_007319:5065926..5481345	248.00	159.79	115.61	146.81	146.47
ADAMTS6	NC_007318:14902334..15177498	115.00	63.47	61.96	69.73	71.61
ADAMTSL1	NC_007306:26844956..27381925	179.00	104.24	92.20	134.85	101.29
ADAR	NC_007301:17351645..17380082	1,852.00	2,160.05	2,278.73	2,599.09	2,499.63
ADAT1	NC_007316:2322596..2338662	54,362.00	51,859.34	53,107.32	59,345.26	62,420.76
ADCY2	NC_007318:69208806..69644735	179.00	111.83	92.56	116.21	109.53
ADCY7	NC_007316:17692814..17771452	1,328.00	1,017.51	1,176.69	1,166.15	1,556.56
ADCYAP1R1	NC_007302:67888374..67946245	315.00	65.56	111.93	112.04	184.16
ADD3	NC_007327:30833757..30964549	1,544.00	947.35	1,149.70	971.79	1,134.91
ADM	NC_007313:41393523..41395806	1,335.00	3,141.82	3,008.15	1,761.42	1,717.26
ADORA3	NC_007301:34320118..34405385	411.00	221.20	133.22	346.02	302.61
ADRBK1	NC_007330:47105450..47123218	2,409.00	2,009.74	2,627.69	3,021.03	2,748.34
ADRM1	NC_007311:55653779..55659514	1,021.00	1,225.58	1,309.71	1,160.04	1,225.31
ADSS	NC_007314:30090031..30122691	360.00	470.03	467.84	438.59	468.28
AFF1	NC_007304:105980394..106119784	665.00	1,209.17	1,113.40	1,061.15	1,302.41
AFF4	NC_007305:44108123..44188792	439.00	884.74	777.93	588.69	768.35
AGA	NC_007328:9465218..9476582	259.00	179.26	158.61	149.51	189.90
AGC1	NC_007319:20147595..20216259	33.00	14.80	6.27	12.49	12.55
AGPAT1	NC_007324:27034295..27044176	795.00	695.88	658.55	703.72	688.07
AGTPBP1	NC_007306:82983196..83183693	303.00	186.42	162.27	240.39	191.80
AHCTF1	NC_007314:27462963..27557027	608.00	756.95	762.03	805.44	788.90
AHNAK	NC_007330:42569995..42574953	5,646.00	11,484.12	10,185.23	8,530.85	13,577.87
AHR	NC_007302:27033540..27083645	535.00	742.23	872.80	703.33	848.55
ALAS1	NC_007320:49685455..49699524	1,010.00	1,979.80	2,277.45	1,831.18	2,712.79
ALDH1A1	NC_007306:51403770..51456856	32.00	149.13	98.10	67.43	173.84
ALDH2	NC_007315:65572702..65598235	1,360.00	752.02	892.02	822.81	1,056.30
ALDH4A1	NC_007300:138380220..138413476	157.00	219.81	275.37	228.27	264.22
ALDH9A1	NC_007301:3917711..3947247	584.00	423.44	500.73	494.32	499.41
ALK	NC_007309:71887433..72627095	634.00	439.20	316.97	524.95	436.97
ALOX5AP	NC_007310:29923505..29946318	61.00	15.66	9.22	14.66	30.48
ALPK1	NC_007304:14247871..14314308	75.00	144.21	139.84	149.78	138.63
ANGPT1	NC_007312:54783106..55084883	209.00	136.39	114.21	155.24	130.74
ANKH	NC_007318:61874308..62043518	198.00	108.05	97.79	116.89	111.63
ANKRD12	NC_007325:43067959..43132571	1,041.00	1,719.71	1,589.92	1,494.63	1,618.16
ANKRD13A	NC_007315:66559251..66593447	1,755.00	1,443.61	1,318.08	1,212.85	1,252.57
ANKRD17	NC_007304:91163719..91330995	1,025.00	1,246.46	1,339.43	1,481.43	1,705.11
ANKRD44	NC_007300:89675819..89845902	721.00	916.93	923.26	1,297.29	1,250.54
ANTXR2	NC_007304:98000385..98236768	2,898.00	4,922.86	5,310.22	3,286.02	4,805.05
ANXA1	NC_007306:51673583..51691212	638.00	1,536.24	1,380.04	962.29	1,425.21
AOX1	NC_007300:93394313..93466310	53.00	17.65	16.06	20.89	28.52
AP1GBP1	NC_007317:13245125..13351984	669.00	565.42	572.71	578.29	554.41
AP2S1	NC_007316:53724455..53734090	1,212.00	864.43	647.37	729.91	641.07
AP3D1	NC_007305:20140485..20174011	2,873.00	3,780.69	3,506.75	3,505.29	3,674.10

Continued on next page...

Table 1 – continued from previous page

Gene	Location	TCT1	TCT3	TCT4	TCT5	TCT6
AP3S2	NC_007319:21009346..21050537	356.00	255.22	259.78	254.89	288.06
API5	NC_007313:73430180..73457295	688.00	901.57	973.63	792.48	806.34
APLP2	NC_007330:37963995..38026747	2,430.00	4,277.00	3,575.53	3,158.67	2,909.77
APOA1BP	NC_007301:15693725..15696624	376.00	248.59	272.46	280.33	257.45
APOBEC3A	NC_007303:117607087..117619028	623.00	8,513.00	7,192.24	5,221.76	9,210.52
AQP3	NC_007306:79337490..79343445	61.00	31.96	30.24	30.05	32.33
ARAF	NC_007331:56264861..56275779	653.00	526.86	747.24	850.04	809.11
ARCN1	NC_007313:27662264..27690872	1,142.00	1,450.45	1,599.10	1,436.82	1,427.46
ARF1	NC_007305:2918822..2936881	2,679.00	3,296.36	3,446.10	3,210.56	3,155.71
ARFGEF1	NC_007312:31219174..31406114	1,038.00	1,390.02	1,454.71	1,366.36	1,462.01
ARFGEF2	NC_007311:78104206..78184475	684.00	807.20	905.55	851.67	907.85
ARFRP1	NC_007311:54723979..54730154	568.00	680.88	692.21	858.20	739.17
ARHGAP1	NC_007313:76970779..76987452	1,290.00	693.77	901.06	996.46	1,109.71
ARHGAP15	NC_007300:55212740..55809064	732.00	567.97	582.86	898.18	634.28
ARHGAP17	NC_007326:23908150..24000651	794.00	1,016.20	956.19	929.84	949.37
ARHGAP25	NC_007309:69024511..69116669	1,180.00	903.53	959.85	972.19	1,021.99
ARHGAP26	NC_007305:53297254..53895494	134,827.00	53,358.28	40,026.97	71,036.90	50,701.39
ARHGAP30	NC_007301:9098206..9113180	4,759.00	3,617.16	3,497.76	3,402.36	3,722.35
ARHGAP9	NC_007303:60444226..60451833	397.00	285.94	313.08	314.13	301.41
ARHGDIB	NC_007303:101897689..101916582	4,152.00	2,940.57	3,502.34	3,501.04	3,519.32
ARHGEF1	NC_007316:51054494..51071433	3,642.00	3,226.06	3,016.53	2,889.84	3,439.83
ARHGEF11	NC_007301:15294878..15345588	263.00	130.61	203.51	119.68	158.08
ARHGEF3	NC_007320:45158968..45228124	1,122.00	581.53	607.68	1,002.54	819.38
ARID1A	NC_007300:130668904..130737823	1,686.00	2,003.13	1,840.91	2,223.81	2,288.65
ARID5A	NC_007309:2838506..2850175	969.00	1,427.76	1,512.70	1,401.83	1,403.89
ARMC8	NC_007299:132863623..132970686	365.00	448.28	558.15	562.60	522.72
ARPC1B	NC_007310:56595499..56596944	3,497.00	3,207.86	3,272.35	2,720.23	2,899.43
ARRDC4	NC_007319:7522025..7536366	667.00	1,911.54	1,452.14	1,008.93	1,336.36
ARSG	NC_007317:63577400..63612942	193.00	129.41	126.51	68.24	149.36
ASCC3	NC_007307:51214258..51556887	403.00	480.17	486.05	489.61	512.49
ASPSCR1	NC_007317:52377558..52405315	782.00	522.04	565.91	627.23	637.95
ASRGL1	NC_007330:42448243..42466938	643.00	425.67	205.04	467.79	397.62
ASXL1	NC_007311:62682338..62741337	1,463.00	1,780.14	1,954.22	2,037.81	2,067.12
ATAD1	NC_007327:9790454..9831330	847.00	1,231.72	1,436.18	1,262.18	1,261.09
ATF3	NC_007314:68874132..68887086	800.00	1,646.28	1,681.78	1,075.39	1,423.11
ATG16L2	NC_007313:51861012..51876539	210.00	277.34	286.82	327.84	383.21
ATP11A	NC_007310:84503055..84551415	843.00	1,293.53	1,212.26	974.92	1,658.12
ATP11B	NC_007299:86096361..86199921	755.00	977.99	1,149.99	941.51	1,143.85
ATP13A3	NC_007299:74431758..74486490	799.00	1,429.71	1,630.19	1,106.75	1,301.00
ATP1A1	NC_007301:29323173..29344077	5,069.00	10,100.11	9,741.52	6,179.66	9,653.32
ATP2A2	NC_007315:56977046..57034513	2,725.00	3,583.28	3,662.09	3,301.27	3,658.35
ATP2A3	NC_007317:24651968..24685977	2,201.00	1,360.24	1,660.32	1,866.53	1,942.95
ATP2B1	NC_007303:22130102..22262198	553.00	724.33	720.29	664.03	652.95
ATP4B	NC_007310:84907242..84912171	62.00	15.66	9.22	30.05	34.29
ATP5A1	NC_007325:47456738..47465645	3,296.00	3,094.84	3,528.66	3,767.54	3,496.10
ATP6V0B	NC_007301:108957203..108961227	2,114.00	1,667.79	1,782.02	1,450.44	1,654.38
ATP6V1A	NC_007299:59265286..59329329	1,345.00	2,585.98	2,670.47	1,810.77	2,085.90
ATR	NC_007299:128603949..128724855	377.00	498.85	514.85	466.53	535.95
AUH	NC_007306:90808901..91003539	669.00	827.09	878.48	906.59	857.71
AZI1	NC_007317:52991344..53004931	104.00	52.20	53.73	69.62	71.52
AZIN1	NC_007312:59765276..59795942	1,283.00	1,630.81	1,838.19	1,532.32	1,636.83
B3GAT1	NC_007313:84564169..84572371	117.00	47.19	54.43	74.47	24.75

Continued on next page...

Table 1 – continued from previous page

Gene	Location	TCT1	TCT3	TCT4	TCT5	TCT6
B3GNT5	NC_007299:85739906..85802333	430.00	507.43	627.44	530.32	513.62
B4GALT2	NC_007301:108946326..108960858	2,176.00	1,743.63	1,877.75	1,456.29	1,741.48
B4GALT5	NC_007311:78750008..78770490	637.00	964.88	868.64	801.91	1,039.13
BACH1	NC_007299:6048792..6064735	282.00	399.43	474.43	194.45	348.20
BAG3	NC_007327:40280054..40303491	208.00	685.96	1,052.18	271.70	717.87
BAT2	NC_007324:27461346..27476474	2,873.00	3,076.94	2,519.16	2,286.64	2,473.86
BAT4	NC_007324:27437573..27440869	762.00	632.75	594.71	476.54	420.45
BATF2	NC_007330:45025771..45031628	86.00	253.45	299.46	131.54	154.28
BAZ1A	NC_007319:45912454..46003648	1,212.00	2,461.93	2,140.95	1,824.19	2,146.83
BAZ2A	NC_007303:61368287..61397772	1,318.00	1,965.18	1,653.11	1,608.53	1,749.77
BCAS3	NC_007317:10957337..11543629	968.00	797.76	730.51	807.13	705.67
BCL2	NC_007325:64011852..64217313	337.00	619.13	451.38	676.56	540.80
BCL2L13	NC_007303:116102891..116154028	748.00	543.06	587.65	528.70	583.70
BEGAIN	NC_007319:65524518..65552022	109.00	52.52	64.08	58.72	75.33
BEST1	NC_007330:42108642..42118471	20.00	69.86	40.47	43.56	67.60
BGN	NC_007331:23017011..23030799	27.00	0.78	0.81	1.54	1.49
BHLHB2	NC_007320:22079281..22084924	4,313.00	9,611.50	8,819.35	5,678.00	7,532.94
BID	NC_007303:116157362..116174872	1,591.00	1,004.21	1,063.32	1,065.81	1,057.25
BIRC3	NC_007313:5396019..5426778	3,841.00	7,760.59	7,765.72	7,768.54	6,587.22
BLNK	NC_007327:17957045..18059093	1,255.00	681.21	657.52	955.98	771.34
BLOC1S1	NC_007303:62103542..62106757	497.00	357.24	366.57	393.37	339.01
BLVRB	NC_007316:49429540..49446560	363.00	438.76	491.73	441.92	575.00
BOLA	NC_007324:28655774..28659565	34,191.00	36,472.69	35,845.91	38,188.83	38,773.26
BOLA-DQA1	NC_007324:26297690..26301588	503.00	1,033.78	1,483.94	838.56	901.14
BOLA-DRB2	NC_007324:26397092..26438196	708.00	1,198.78	856.36	606.45	605.68
BOLL	NC_007300:90432467..90501343	982.00	440.42	388.57	812.39	640.28
BRWD3	NC_007331:43610026..43862017	190.00	294.35	271.50	292.08	331.37
BST2	NC_007305:5686588..5689315	99.00	352.07	267.68	329.27	204.59
BTAF1	NC_007327:13881111..14007549	746.00	1,063.00	1,030.08	1,021.39	1,074.31
BTG3	NC_007299:19111628..19130585	311.00	763.71	833.38	517.25	520.23
BXDC5	NC_007301:63570871..63587351	420.00	301.25	354.13	299.19	274.87
BZRAP1	NC_007317:8488620..8513481	221.00	128.67	145.56	161.29	149.99
C12H13orf27	NC_007310:76784924..76791570	123.00	170.66	180.12	183.99	165.35
C13H20orf149	NC_007311:54812588..54814239	535.00	383.44	403.27	319.78	325.42
C14H8orf46	NC_007312:30640296..30666569	9.00	48.83	35.01	77.26	42.58
C16H1orf93	NC_007314:47581073..47584187	170.00	61.82	87.96	90.23	88.59
C19H17orf61	NC_007317:27497399..27498549	425.00	330.13	328.53	353.59	261.38
C19H17orf62	NC_007317:51609980..51616022	456.00	316.90	331.25	326.71	345.55
C1H3orf26	NC_007299:45123362..45518364	1,559.00	1,354.28	1,037.39	1,394.20	1,338.22
C1QA	NC_007300:134672736..134675624	88.00	12.84	22.48	5.22	21.02
C1QB	NC_007300:134649446..134656193	141.00	42.52	85.10	17.16	49.54
C1QC	NC_007300:134664248..134668596	81.00	18.45	41.75	12.68	13.51
C1QTNF6	NC_007303:81256864..81264139	14.00	1.52	3.57	3.51	2.64
C2	NC_007324:27213701..27225299	755.00	3,164.46	2,551.39	1,443.16	1,571.59
C29H11orf10	NC_007330:41890967..41894655	399.00	482.53	677.16	603.68	575.75
C3H1orf164	NC_007301:108234395..108271816	1,456.00	1,108.11	998.79	1,242.96	1,130.45
C3H1orf85	NC_007301:15842688..15845541	446.00	360.94	332.71	256.27	303.26
C4orf32	NC_007304:14486290..14529063	235.00	338.14	419.51	365.82	340.46
C5AR1	NC_007316:54104908..54123672	899.00	438.76	430.07	356.09	590.24
C5H22orf32	NC_007303:120198466..120202167	615.00	455.84	529.88	450.16	430.68
C6ORF27	NC_007324:27349792..27359109	14.00	40.89	44.24	39.40	34.66
C7H5orf32	NC_007305:50688959..50759632	788.00	1,135.57	1,110.58	989.34	1,034.47

Continued on next page...

Table 1 – continued from previous page

Gene	Location	TCT1	TCT3	TCT4	TCT5	TCT6
C8H9orf80	NC_007306:107139121..107165115	5,043.00	4,229.79	4,531.04	4,788.73	4,677.20
C9orf89	NC_007306:88654335..88669776	444.00	307.40	272.17	326.87	344.09
CA2	NC_007312:75898446..75914801	149.00	91.77	52.52	75.11	77.70
CA6	NC_007314:41524317..41549081	14.00	3.17	3.03	2.96	3.52
CAB39	NC_007300:122683702..122806003	604.00	852.75	959.39	778.82	959.30
CABC1	NC_007314:26957896..27005320	87.00	19.82	19.71	29.58	25.31
CACNA1B	NC_007309:110097645..110171688	141.00	65.81	58.04	101.18	57.21
CALCOCO1	NC_007303:29228196..29242321	910.00	459.63	540.58	577.08	616.01
CALCOCO2	NC_007317:38884778..38900924	879.00	1,212.45	1,110.73	1,160.03	1,026.54
CALR	NC_007305:10883303..10887133	8,208.00	11,899.35	11,341.48	10,039.25	10,963.09
CAMKK1	NC_007317:24594779..24623204	83.00	54.41	30.61	26.88	34.81
CAMKK2	NC_007315:56623304..56655083	60.00	102.21	92.65	156.28	90.82
CAMLG	NC_007305:45443856..45451614	131.00	192.45	234.76	175.76	191.51
CAND1	NC_007303:50301462..50335768	881.00	1,002.69	1,181.00	1,119.80	1,087.59
CAP1	NC_007301:113134310..113169361	4,273.00	3,630.50	3,794.99	3,355.53	3,517.81
CAPG	NC_007309:51351786..51367047	1,150.00	919.06	861.64	706.78	957.62
CAPN10	NC_007301:125930022..125940175	544.00	240.16	222.25	300.49	334.23
CAPN7	NC_007299:155717663..155776866	548.00	656.57	697.69	664.82	763.40
CAPRIN1	NC_007313:64373885..64409057	2,078.00	2,783.35	3,074.67	2,893.07	3,040.13
CAPZA1	NC_007301:33181590..33235417	2,503.00	3,079.96	3,100.79	2,779.90	2,826.12
CARD14	NC_007317:53948408..53969135	29.00	7.35	8.16	6.89	12.02
CARD9	NC_007309:107730226..107737290	355.00	135.31	186.02	113.64	131.59
CARS	NC_007330:50426969..50480177	855.00	1,069.86	1,298.56	1,475.67	1,188.08
CASP3	NC_007328:16446490..16471673	217.00	134.45	120.12	159.58	153.07
CASP4	NC_007313:2097363..2116795	521.00	1,100.26	1,122.22	821.62	932.48
CASP8	NC_007300:94153163..94176829	1,113.00	1,711.93	1,746.17	1,415.15	1,333.11
CBLL1	NC_007302:50782839..50797465	154.00	230.24	218.02	227.53	250.88
CBX6	NC_007303:117540671..117548573	316.00	209.34	191.84	235.60	222.27
CC2D1B	NC_007301:100750233..100766072	1,595.00	1,037.19	996.78	1,099.21	1,030.44
CCBL2	NC_007301:58327621..58388019	184.00	442.40	358.81	329.76	452.95
CCDC109B	NC_007304:17122923..17217867	307.00	438.42	559.34	511.06	617.72
CCDC117	NC_007315:71572489..71584561	230.00	400.63	409.15	314.57	313.90
CCDC23	NC_007301:110374993..110381467	97.00	56.88	54.22	54.64	46.46
CCDC60	NC_007315:58723101..58921890	112.00	73.86	62.22	72.80	73.14
CCDC69	NC_007305:62099731..62137873	879.00	508.04	611.60	491.22	568.38
CCDC72	NC_007303:13350820..13351223	424.00	317.80	324.49	324.65	260.52
CCDC72	NC_007324:25314441..25315140	810.00	672.21	587.13	526.41	428.98
CCDC80	NC_007299:58110067..58145589	461.00	268.28	200.17	185.23	107.52
CCL16	NC_007317:13929310..13934358	34.00	14.71	5.44	8.27	13.29
CCL2	NC_007317:15665286..15667153	3,704.00	5,343.16	8,753.23	1,855.50	3,086.58
CCL22	NC_007316:25284633..25292673	635.00	357.72	261.65	480.93	433.43
CCL3	NC_007317:13822062..13823579	11,481.00	12,123.76	13,085.98	9,529.07	12,366.55
CCL5	NC_007317:13970868..13977618	2,787.00	7,965.84	7,024.22	7,310.33	6,059.86
CCL8	NC_007317:15403977..15406012	421.00	983.05	1,654.88	244.69	587.95
CCL8	NC_007317:15607468..15609431	418.00	964.67	1,633.11	241.24	577.19
CCND2	NC_007303:112629798..112652687	436.00	848.72	1,051.91	1,325.25	1,119.24
CCND3	NC_007324:16321575..16328397	1,457.00	685.12	824.76	947.53	847.73
CCNL1	NC_007299:112620776..112634658	803.00	1,196.56	1,362.66	1,032.97	1,324.99
CCNL2	NC_007314:48411159..48419299	984.00	1,227.77	1,168.45	1,318.73	1,348.58
CCNT2	NC_007300:65311400..65344248	450.00	573.58	635.85	590.83	615.51
CCNY	NC_007311:17340385..17376434	734.00	994.44	1,108.08	996.88	1,095.62
CCR1	NC_007320:54585476..54591526	2,841.00	4,681.60	3,924.99	2,419.05	3,616.35

Continued on next page...

Table 1 – continued from previous page

Gene	Location	TCT1	TCT3	TCT4	TCT5	TCT6
CCR4	NC_007320:7326634..7331581	142.00	307.91	321.85	245.71	376.02
CCR5	NC_007320:54389968..54393154	49.00	125.78	154.79	128.13	111.54
CCT2	NC_007303:47359373..47375180	1,416.00	1,680.00	2,013.26	1,880.59	1,805.17
CCT4	NC_007309:62453931..62466568	1,471.00	1,767.14	2,229.24	2,107.88	1,910.95
CCT5	NC_007318:66718266..66732518	1,410.00	1,641.90	2,018.41	2,141.02	2,217.25
CCT6A	NC_007326:29552510..29562368	890.00	1,294.50	1,579.01	1,479.56	1,299.76
CCT7	NC_007309:11663192..11680487	1,912.00	2,119.08	2,276.12	2,755.27	2,550.32
CCT8	NC_007299:6393356..6407101	1,839.00	2,645.98	3,461.51	2,517.86	2,878.25
CD14	NC_007305:51085556..51086972	4,214.00	5,250.09	3,906.11	3,440.05	3,378.76
CD163	NC_007303:109174623..109205926	81.00	20.48	37.08	17.53	52.76
CD163L1	NC_007303:10001847..10064425	1,629.00	590.09	1,255.16	741.86	1,318.43
CD164	NC_007307:42598353..42608945	1,227.00	3,009.12	2,728.50	2,354.83	3,686.05
CD27	NC_007303:110721039..110726613	932.00	582.50	521.32	1,064.41	831.68
CD274	NC_007306:41389450..41425826	2,099.00	5,858.86	6,931.91	3,695.25	5,947.97
CD300LB	NC_007317:58640585..58650727	40.00	18.38	11.56	11.72	13.29
CD38	NC_007304:115601901..115658026	507.00	1,210.00	1,048.32	966.74	884.62
CD3D	NC_007313:27451132..27455296	2,073.00	1,758.43	2,563.23	2,779.74	3,308.38
CD3E	NC_007313:27435481..27446877	4,059.00	3,286.70	4,649.58	5,067.32	5,149.38
CD40	NC_007311:75614963..75625707	526.00	1,017.74	909.15	787.23	825.89
CD40LG	NC_007331:10095411..10107233	12.00	51.67	34.04	91.71	47.55
CD44	NC_007313:65364148..65451398	3,734.00	7,215.02	5,680.23	5,304.50	7,056.65
CD46	NC_007314:73893897..73926369	877.00	1,264.23	1,126.75	1,006.61	1,151.71
CD47	NC_007299:55589613..55654685	2,384.00	2,990.55	3,494.23	2,624.69	3,006.44
CD53	NC_007301:35138238..35177059	3,457.00	4,633.28	5,623.20	5,060.28	6,120.03
CD69	NC_007303:107783338..107790440	212.00	813.07	821.56	984.47	825.75
CD72	NC_007306:62457024..62464416	397.00	560.78	534.02	652.28	598.39
CD74	NC_007305:61216227..61223986	13,865.00	19,180.71	23,707.78	14,644.41	18,160.55
CD79A	NC_007316:51076128..51079791	1,163.00	614.76	874.43	624.93	898.07
CD83	NC_007324:43226520..43246562	426.00	706.64	916.40	746.82	637.50
CD84	NC_007301:10008224..10070767	406.00	138.34	249.70	200.98	312.94
CD9	NC_007303:110986876..111021621	1,385.00	2,665.24	2,724.99	1,170.29	1,910.70
CD97	NC_007305:9404243..9421531	6,063.00	7,465.60	7,843.40	6,466.41	8,078.10
CDC123	NC_007311:10639038..10685425	406.00	559.04	567.09	601.23	551.39
CDC14B	NC_007306:87385285..87505019	147.00	90.55	106.95	107.74	104.16
CDC25B	NC_007311:51954757..51964354	1,781.00	619.96	910.95	991.17	1,063.00
CDC27	NC_007317:47725723..47783192	309.00	436.75	466.60	470.08	447.45
CDC2L1	NC_007314:48256028..48276017	7,523.00	5,418.37	5,546.54	6,126.94	5,372.53
CDC37	NC_007305:13386561..13387524	465.00	789.89	695.63	788.25	855.27
CDC37L1	NC_007306:42031362..42046326	117.00	267.07	295.78	187.62	212.63
CDC42	NC_007300:135218936..135239719	2,194.00	2,474.03	2,845.26	2,014.19	1,831.52
CDC42BPG	NC_007330:44845347..44863739	103.00	32.49	50.36	57.49	61.23
CDC42EP3	NC_007309:20738541..20763714	54.00	28.10	29.76	30.79	27.18
CDC42SE1	NC_007301:21239222..21246848	2,805.00	2,339.81	2,223.84	2,082.65	2,394.46
CDC42SE2	NC_007305:21636607..21783438	1,722.00	2,155.31	2,498.38	2,564.31	2,203.54
CDH13	NC_007316:8336611..9361675	642.00	401.11	369.17	559.67	536.52
CDH18	NC_007318:56698744..57306541	1,253.00	584.83	425.48	883.20	718.25
CDH3	NC_007316:34990435..35036015	147.00	98.23	91.73	95.05	100.92
CDIPT	NC_007326:28201811..28205237	500.00	341.55	408.68	377.93	358.70
CDK2	NC_007303:61904800..61909483	127.00	84.19	90.57	84.14	84.42
CDK5RAP1	NC_007311:63707526..63742356	132.00	180.07	214.23	208.84	186.18
CDK6	NC_007302:10182420..10430210	310.00	541.65	436.76	397.66	460.06
CDKN1A	NC_007324:10906547..10914830	770.00	1,467.80	1,480.78	913.67	1,294.89

Continued on next page...

Table 1 – continued from previous page

Gene	Location	TCT1	TCT3	TCT4	TCT5	TCT6
CDON	NC_007330:30870430..30933814	69.00	256.70	357.91	150.70	215.51
CDR2L	NC_007317:58048364..58059934	113.00	52.20	50.33	42.05	62.66
CDS2	NC_007311:47627053..47683993	1,721.00	2,283.63	2,292.67	1,996.21	2,066.32
CEBPG	NC_007316:43232178..43239129	129.00	196.46	181.95	200.74	193.12
CECR5	NC_007303:115834639..115842510	380.00	189.62	239.62	285.38	314.60
CENPC1	NC_007304:85854568..85948893	220.00	306.64	321.88	353.11	300.85
CENTA2	NC_007317:17809414..17855933	82.00	22.06	29.93	15.16	10.76
CENTB1	NC_007317:27417668..27429572	1,352.00	746.29	923.00	1,077.32	1,028.44
CFB	NC_007324:27207241..27213267	649.00	3,088.41	1,768.97	1,459.16	1,650.57
CFH	NC_007314:4013443..4110593	122.00	56.28	44.74	72.48	54.84
CFLAR	NC_007300:94017519..94051080	957.00	1,955.00	2,006.29	1,620.30	1,715.17
CHCHD3	NC_007302:99808615..100124752	727.00	594.72	607.23	627.95	581.68
CHCHD6	NC_007301:127444997..127546006	55.00	23.17	22.76	31.87	23.87
CHD1	NC_007305:99580233..99660654	878.00	1,432.92	1,315.90	1,176.62	1,015.24
CHD3	NC_007317:28074680..28096686	2,777.00	2,991.79	2,512.58	3,078.94	3,323.91
CHKA	NC_007330:47541819..47573890	510.00	599.51	896.58	657.69	897.82
CHL1	NC_007320:26645003..26873749	333.00	200.92	177.78	245.00	190.11
CHMP1B	NC_007325:44305884..44307716	510.00	403.47	382.51	399.45	414.80
CHN2	NC_007302:69339757..69380835	50.00	112.39	154.37	85.06	111.89
CHORDC1	NC_007330:5244473..5266436	409.00	784.73	1,007.19	872.86	830.23
CHSY1	NC_007319:3918016..4007728	985.00	797.46	793.98	605.98	734.44
CIB1	NC_007319:21364649..21367821	787.00	672.44	678.36	533.51	621.45
CIRBP	NC_007305:42731969..42736660	1,470.00	1,131.01	1,276.05	1,288.68	1,189.07
CIRH1A	NC_007316:35364239..35394372	273.00	449.41	422.55	532.00	562.66
CISD2	NC_007304:23575297..23588369	221.00	282.61	309.57	331.33	314.99
CISH	NC_007320:50656185..50661604	816.00	1,314.15	1,144.85	1,175.03	1,165.23
CKAP4	NC_007303:74774002..74782784	443.00	307.18	352.30	308.52	284.26
CKAP5	NC_007313:77022343..77129098	731.00	860.17	939.07	908.23	942.86
CKB	NC_007319:68388451..68391652	440.00	227.85	332.22	102.45	212.03
CLDND1	NC_007299:43777452..43784753	652.00	822.85	994.09	889.24	927.16
CLEC12A	NC_007303:107365165..107377681	105.00	56.13	44.22	33.66	29.68
CLEC4G	NC_007305:15012416..15015873	19.00	0.91	0.87	0.77	5.38
CLIC3	NC_007309:109293913..109298356	351.00	190.76	174.11	201.62	175.22
CLINT1	NC_007305:69142027..69199887	906.00	1,430.18	1,325.42	1,190.43	1,209.83
CLIP2	NC_007326:35126314..35178057	131.00	91.92	93.34	49.71	88.64
CLK1	NC_007300:93798513..93803096	601.00	731.62	766.71	925.03	877.48
CLK4	NC_007305:39055033..39076773	414.00	559.12	582.15	609.42	662.98
CLSTN1	NC_007314:40808291..40849437	3,149.00	2,408.78	2,762.28	3,399.99	3,360.91
CLTC	NC_007317:9841655..9906176	3,735.00	4,346.14	4,538.16	4,050.80	4,129.57
CMPK2	NC_007309:92889272..92905102	154.00	245.48	212.06	269.81	239.58
CMTM6	NC_007320:6944421..6966511	2,004.00	2,322.83	2,753.32	2,649.97	2,375.35
CNBP	NC_007320:60874648..60876907	2,272.00	2,107.21	2,534.92	2,992.83	2,541.61
CNNM2	NC_007327:23889074..24082248	159.00	106.14	86.86	102.07	91.96
CNNM4	NC_007309:3004069..3041702	158.00	219.60	260.43	217.02	325.41
CNOT1	NC_007316:26302539..26385842	3,185.00	3,695.94	3,872.17	4,218.43	4,651.54
CNOT6L	NC_007304:95781690..95851414	1,006.00	1,268.84	1,577.76	1,666.61	1,810.73
CNOT7	NC_007328:21710156..21725210	1,084.00	1,385.85	1,363.78	1,207.75	1,542.30
CNPY3	NC_007324:17174924..17183295	623.00	510.79	482.85	496.85	399.43
CNTN4	NC_007320:23867182..24391358	333.00	224.61	165.52	265.42	196.43
CNTNAP1	NC_007317:44076347..44111905	235.00	43.38	43.53	57.21	81.01
COL11A2	NC_007324:7625248..7653478	105.00	53.09	57.51	30.17	41.48
COL18A1	NC_007299:148051968..148104213	91.00	42.86	35.61	33.82	43.84

Continued on next page...

Table 1 – continued from previous page

Gene	Location	TCT1	TCT3	TCT4	TCT5	TCT6
COL6A3	NC_007301:124378979..124428165	25.00	9.83	9.37	8.46	9.03
COLQ	NC_007299:156002897..156040652	58.00	117.43	143.39	108.37	110.83
COMMD4	NC_007319:33477921..33483377	331.00	212.31	184.56	157.43	187.62
COPB1	NC_007313:37016664..37051176	1,022.00	1,359.39	1,548.13	1,522.62	1,433.05
COPB2	NC_007299:132020307..132050596	1,047.00	1,233.42	1,537.81	1,295.05	1,442.43
COPS5	NC_007312:31084644..31103677	375.00	463.90	575.21	557.99	511.74
COQ10B	NC_007300:90211924..90228911	219.00	350.57	403.83	298.40	341.53
COQ2	NC_007304:101821037..101848186	431.00	714.39	630.58	886.86	703.55
CORIN	NC_007304:68797327..69054858	1,020.00	1,277.13	1,200.47	1,276.28	1,320.33
COTL1	NC_007316:10007008..10050587	4,095.00	2,952.66	2,385.71	2,815.36	2,520.70
COX5B	NC_007324:8751222..8751713	696.00	578.23	523.93	430.36	389.20
COX6B2	NC_007316:62514749..62516242	24.00	8.19	9.02	5.67	5.08
COX7A1	NC_007316:46231281..46232845	42.00	103.96	109.02	108.53	106.00
CPNE1	NC_007311:65432558..65468475	1,638.00	1,943.19	2,051.43	2,183.02	2,233.41
CPNE7	NC_007316:13535901..13548047	96.00	13.92	21.05	12.06	34.85
CPNE8	NC_007303:45665007..45944395	254.00	134.25	99.84	174.60	123.99
CPOX	NC_007299:43842874..43855750	115.00	182.57	221.34	181.38	217.52
CPT2	NC_007301:99740894..99765137	207.00	111.65	138.57	159.35	133.56
CREB1	NC_007300:100954834..100992764	418.00	583.21	614.97	558.00	614.01
CREBZF	NC_007330:10233095..10238516	510.00	603.57	641.19	793.59	647.32
CREG1	NC_007301:1711078..1722465	545.00	688.88	684.16	271.23	416.17
CREM	NC_007311:17474894..17541383	359.00	777.64	851.54	748.88	786.11
CRIM2	NC_007302:96081909..96111023	66.00	99.12	117.06	36.65	104.94
CRISP2	NC_007324:22865822..22891276	73.00	19.37	20.86	36.94	34.09
CRISP3	NC_007324:22899522..22924354	22,673.00	18,971.68	17,233.44	20,257.13	19,107.37
CRLS1	NC_007311:48423439..48446513	378.00	511.56	625.60	450.31	475.80
CRTAP	NC_007320:7475142..7508541	51.00	25.32	28.35	26.75	27.36
CSE1L	NC_007311:78200246..78235285	558.00	724.78	848.84	846.78	765.42
CSF1R	NC_007305:60848894..60880399	2,395.00	1,779.52	1,610.43	1,117.52	1,395.96
CSF2RA	NC_007312:19935960..19938069	1,561.00	1,396.75	1,286.31	841.27	1,245.36
CSF2RB	NC_007303:81005400..81026598	2,494.00	4,313.39	2,775.63	1,703.89	2,669.17
CSF3	NC_007317:41596177..41598488	9,130.00	6,431.35	4,810.23	5,635.42	4,125.14
CSK	NC_007319:33944209..33962530	3,933.00	2,974.58	3,102.77	3,668.38	4,257.39
CSMD1	NC_007328:2110278..3158083	539.00	286.82	237.58	357.34	348.75
CSNK2A1	NC_007311:61344322..61401557	623.00	857.37	958.66	944.67	883.18
CSPG4	NC_007319:33139028..33196843	75.00	11.84	29.25	26.86	34.87
CSRP2BP	NC_007311:38300684..38326734	355.00	463.18	499.58	601.23	566.51
CSTF2	NC_007331:29862550..29893509	216.00	278.70	320.13	305.95	288.28
CSTF3	NC_007313:63295095..63363143	361.00	432.98	480.34	443.59	473.42
CTDSP1	NC_007300:110809985..110814953	807.00	484.54	565.20	475.94	583.23
CTDSP2	NC_007303:60067857..60088302	1,253.00	1,045.16	1,026.95	935.39	1,040.10
CTH	NC_007305:49386445..49388340	12.00	33.75	40.72	42.98	31.53
CTNNA1	NC_007305:49391917..51309334	22,991.00	29,450.12	27,430.23	23,977.53	23,907.44
CTNNA2	NC_007309:56353028..57788832	1,077.00	754.91	633.21	963.39	781.69
CTNND2	NC_007318:64883743..66006336	450.00	333.97	244.84	381.45	353.16
CTR9	NC_007313:40917111..40942225	877.00	1,026.58	1,206.02	1,124.36	988.36
CTSB	NC_007306:7457578..7466062	19,571.00	22,857.17	21,877.80	11,647.88	17,065.21
CTSC	NC_007330:7498212..7577365	3,899.00	5,373.66	4,303.05	3,527.15	3,681.95
CTSD	NC_007330:51565953..51575395	7,251.00	6,957.73	6,890.27	8,234.67	8,421.56
CTSF	NC_007330:46509539..46515103	204.00	89.36	90.83	96.69	86.68
CTSH	NC_007319:24945845..24966570	5,532.00	6,120.04	4,049.28	2,313.98	4,548.24
CTSL1	NC_007306:85316468..85322244	404.00	97.39	135.72	115.80	99.85

Continued on next page...

Table 1 – continued from previous page

Gene	Location	TCT1	TCT3	TCT4	TCT5	TCT6
CTSZ	NC_007311:58097662..58107159	8,394.00	11,393.98	10,334.58	7,456.21	9,894.01
CTTN	NC_007330:49361185..49388227	538.00	406.98	302.16	381.55	283.10
CUGBP1	NC_007313:77639335..77717883	622.00	786.49	830.22	841.05	802.35
CUL2	NC_007311:17570692..17642083	303.00	379.86	439.27	571.86	456.71
CUL3	NC_007300:117134164..117250145	823.00	926.56	1,062.96	966.06	950.29
CUX2	NC_007315:57628048..57905960	115.00	60.43	39.22	55.43	56.08
CXCL10	NC_007304:94129095..94131447	378.00	1,051.70	1,919.88	3,041.84	2,180.43
CXCL16	NC_007317:26983347..26986831	2,103.00	5,133.61	3,700.86	3,142.35	3,536.56
CXCL2	NC_007304:91927492..91929555	61,045.00	83,165.69	71,066.17	54,562.53	64,500.81
CXCL2	NC_007304:92071945..92073356	30,240.00	39,138.40	32,225.41	27,246.77	37,713.26
CXCL2	NC_007304:92071948..92073927	45,819.00	62,547.68	57,452.91	48,269.16	64,424.42
CXCL3	NC_007304:92060262..92062287	10,491.00	11,729.50	11,757.59	11,422.11	11,846.16
CXCL9	NC_007304:94104336..94110153	15.00	43.92	122.25	66.27	112.23
CXCR5	NC_007313:27932325..27943201	211.00	365.93	538.22	622.49	515.74
CXCR6	NC_007320:54734258..54739599	252.00	605.21	476.64	858.92	573.85
CXXC5	NC_007305:50191003..50198220	138.00	83.91	88.36	76.75	99.99
CYB5R1	NC_007314:77892495..77897602	355.00	241.75	249.46	250.73	241.17
CYB5R3	NC_007303:120632335..120647393	1,267.00	746.33	1,026.24	917.16	1,150.90
CYBA	NC_007316:12880237..12887205	2,466.00	2,116.87	1,803.00	1,680.42	1,643.68
CYBASC3	NC_007330:41515514..41526841	450.00	335.50	615.11	672.50	570.98
CYGB	NC_007317:56885298..56893238	147.00	39.70	46.25	31.02	54.43
CYLD	NC_007316:18182789..18251354	761.00	989.68	1,041.35	1,040.60	1,028.75
CYP1A1	NC_007319:34019575..34025624	26.00	10.36	7.66	10.00	10.46
CYP20A1	NC_007300:96279266..96323424	389.00	259.92	241.65	233.50	216.18
CYP51	NC_007302:9698100..9714943	514.00	1,276.08	1,427.35	1,409.43	1,417.02
DAPP1	NC_007304:26500034..26556774	206.00	303.33	332.44	398.50	446.08
DARS	NC_007300:64355412..64420348	1,035.00	1,237.82	1,353.59	1,365.17	1,318.09
DAZAP1	NC_007305:42848228..42867177	1,067.00	1,470.32	1,345.35	1,347.01	1,500.56
DAZAP2	NC_007303:31525582..31529790	2,892.00	2,663.73	2,649.40	2,604.22	2,560.35
DBN1	NC_007305:38263907..38277762	236.00	74.79	67.57	50.66	81.53
DC2	NC_007304:18047065..18057479	284.00	413.55	506.57	522.86	410.24
DCBLD2	NC_007299:44085699..44176079	498.00	949.71	710.20	605.64	724.52
DCT	NC_007310:67906694..67989079	2,663.00	5,600.35	6,263.21	3,680.49	5,318.79
DCTN4	NC_007305:61688769..61719844	247.00	367.58	367.31	310.08	346.87
DCUN1D3	NC_007326:19734609..19789060	163.00	285.04	243.95	234.65	230.59
DDB1	NC_007330:41473844..41501698	1,763.00	1,578.39	2,302.15	2,343.31	2,248.93
DDB2	NC_007313:77448663..77468938	1,347.00	1,512.55	1,533.45	1,100.08	1,485.74
DDT	NC_007315:74619374..74622047	300.00	185.77	182.60	153.61	171.89
DDX18	NC_007302:7535589..7539433	435.00	576.77	561.87	525.60	508.71
DDX41	NC_007305:38321346..38326923	812.00	954.98	980.64	1,084.52	1,185.22
DDX46	NC_007305:45458051..45510284	927.00	1,359.95	1,282.98	1,221.90	1,058.31
DDX58	NC_007306:11649082..11694263	438.00	620.89	595.98	762.94	580.43
DEF6	NC_007324:9538448..9565419	726.00	386.68	466.42	568.89	539.20
DEFB7	NC_007328:7372715..7405372	30.00	11.03	12.61	8.48	13.00
DENND1A	NC_007309:97749762..98281513	4,586.00	3,523.92	3,827.51	3,989.21	3,681.73
DENND2D	NC_007301:34801732..34817440	469.00	219.80	275.13	303.10	313.58
DENND3	NC_007312:1997561..2047080	1,228.00	1,437.17	1,489.72	1,390.68	1,908.01
DENND4C	NC_007306:26439673..26523184	590.00	770.78	697.03	856.75	825.39
DGAT2	NC_007313:54717390..54749894	66.00	28.98	29.37	16.19	39.92
DGKB	NC_007302:23369212..24166922	375.00	265.36	184.36	294.61	268.25
DGKI	NC_007302:104679165..104999295	102.00	54.63	53.41	63.63	60.46
DHCR24	NC_007301:97959329..98014759	2,106.00	1,728.18	2,448.10	2,471.60	2,769.34

Continued on next page...

Table 1 – continued from previous page

Gene	Location	TCT1	TCT3	TCT4	TCT5	TCT6
DHCR7	NC_007330:50153773..50167388	374.00	601.14	777.88	1,049.70	860.45
DHPS	NC_007305:11085063..11088973	516.00	612.02	614.20	665.45	658.37
DHRS3	NC_007314:37841737..37891523	239.00	123.65	103.12	69.52	90.00
DHX15	NC_007304:45484486..45538459	1,071.00	1,583.77	1,713.20	2,072.37	2,168.17
DHX36	NC_007299:115693128..115738065	428.00	546.85	647.65	538.77	593.85
DHX40	NC_007317:9776943..9828637	262.00	177.93	201.33	205.40	186.07
DHX9	NC_007314:61435090..61476091	1,946.00	2,337.44	2,327.80	2,678.87	2,375.84
DLGAP1	NC_007325:38970595..39268228	193.00	132.90	104.05	126.03	133.08
DMAP1	NC_007301:108709129..108718299	265.00	172.75	192.80	184.72	194.21
DMBT1	NC_007327:43017754..43062960	685.00	1,983.42	551.61	1,099.01	811.97
DMP1	NC_007304:106577198..106593906	26.00	5.80	8.79	5.45	8.49
DNAI1	NC_007306:80048270..80117111	4,846.00	3,508.47	3,889.34	4,448.66	4,506.95
DNAJA1	NC_007306:78911556..78921312	892.00	1,569.72	1,634.51	1,452.60	1,507.11
DNAJA2	NC_007316:14507531..14519090	728.00	921.73	961.65	925.68	917.67
DNAJA4	NC_007319:30710902..30725154	175.00	381.72	541.16	306.74	514.74
DNAJB1	NC_007305:9502440..9506120	431.00	1,329.85	2,051.38	757.55	1,297.51
DNAJB6	NC_007302:122897893..122931880	537.00	681.36	714.77	652.16	679.67
DNAJC13	NC_007299:139255393..139419812	1,117.00	1,541.99	1,635.96	1,326.56	1,418.45
DNAJC21	NC_007318:41581671..41608636	222.00	369.48	373.23	358.89	345.44
DNM2	NC_007305:13659605..13747930	3,605.00	5,085.00	4,341.85	4,003.42	4,512.45
DNMT1	NC_007305:13150582..13193559	1,032.00	1,417.41	1,268.25	1,452.93	1,426.72
DOK2	NC_007306:72427643..72432271	487.00	345.44	351.83	313.68	369.95
DOK6	NC_007325:7510290..7749029	482.00	242.72	191.63	342.70	245.63
DOK7	NC_007304:120336197..120360940	195.00	44.75	89.71	84.42	132.03
DPF2	NC_007330:45353138..45365799	1,063.00	1,220.14	1,473.02	1,327.58	1,339.18
DPY19L4	NC_007312:67860002..67921974	148.00	219.74	211.75	203.16	224.87
DPYD	NC_007301:48406400..49287303	498.00	344.09	230.95	360.98	315.52
DTNA	NC_007325:23235860..23555006	125.00	70.83	44.16	71.49	57.34
DTX1	NC_007315:64292283..64329942	207.00	132.80	149.46	139.58	103.62
DTX3L	NC_007299:68074820..68083505	856.00	1,243.71	1,600.36	1,196.68	1,341.53
DUSP1	NC_007318:4575773..4578856	1,280.00	2,408.07	2,808.90	1,618.75	2,616.05
DUSP28	NC_007301:125908421..125911374	277.00	195.92	219.57	198.38	221.96
DUSP5	NC_007327:31334903..31347776	760.00	1,156.47	1,272.37	871.16	1,072.17
DUSP6	NC_007303:21812163..21815843	1,631.00	1,401.64	1,374.98	844.23	1,151.56
DYM	NC_007325:50390159..50791554	1,531.00	992.36	785.23	1,288.27	998.08
DYRK1A	NC_007299:152985413..153079821	1,089.00	1,373.13	1,641.74	1,265.85	1,414.31
EBI2	NC_007310:73934020..73948659	549.00	982.72	961.77	679.51	767.34
ECM1	NC_007301:21721706..21727111	2,378.00	1,419.90	1,162.80	1,011.41	1,203.36
EDF1	NC_007309:109384992..109388900	879.00	697.98	759.70	733.91	703.73
EDG1	NC_007301:44986591..44991246	654.00	240.16	318.65	426.03	403.92
EDG6	NC_007305:19281106..19282528	307.00	129.52	182.79	184.97	211.51
EDN1	NC_007324:44797047..44804030	479.00	1,608.41	1,375.87	814.55	1,284.66
EED	NC_007330:9610575..9641117	411.00	618.19	759.89	859.79	880.33
EEPD1	NC_007302:63399086..63520895	69.00	35.90	37.31	33.20	29.88
EFHD2	NC_007314:49324205..49342321	3,968.00	4,382.41	4,361.41	2,989.51	3,486.06
EFR3A	NC_007312:8559689..8601402	889.00	1,106.29	1,189.61	992.93	1,122.84
EGFL8	NC_007324:27044103..27046835	69.00	28.70	41.08	27.09	33.52
EGFLAM	NC_007318:38212516..38406759	190.00	284.86	138.09	84.77	117.25
EGR1	NC_007305:49142552..49145395	209.00	558.21	589.94	468.19	772.97
EHBP1L1	NC_007330:45620537..45635847	2,478.00	2,134.84	1,872.30	1,631.60	1,923.68
EHMT1	NC_007309:109972835..110062937	1,333.00	1,008.52	1,096.02	1,491.28	1,624.88
EIF2A	NC_007299:119352717..119397241	664.00	801.42	1,033.54	792.40	865.96

Continued on next page...

Table 1 – continued from previous page

Gene	Location	TCT1	TCT3	TCT4	TCT5	TCT6
EIF2AK1	NC_007326:39833375..39860281	742.00	926.81	920.89	919.61	964.61
EIF2AK2	NC_007309:20215972..20256666	685.00	1,222.21	1,192.75	1,340.98	1,066.33
EIF2C2	NC_007312:2373025..2409522	467.00	755.21	736.94	669.59	1,021.24
EIF2C4	NC_007301:117160029..117198140	233.00	171.34	157.99	139.84	178.08
EIF2S2	NC_007311:64338649..64357232	808.00	1,224.69	1,144.09	1,005.85	968.32
EIF3A	NC_007327:39704533..39735630	2,644.00	3,460.69	3,011.78	2,982.94	2,928.52
EIF3B	NC_007326:42757181..42775477	2,008.00	2,187.58	2,240.26	2,418.91	2,195.81
EIF3C	NC_007326:27821996..27842171	2,835.00	3,337.87	3,441.97	3,456.94	3,242.82
EIF3E	NC_007312:54054335..54102285	2,248.00	2,047.56	2,485.93	1,937.94	2,050.70
EIF3F	NC_007313:43601629..43612148	2,050.00	1,568.84	2,240.13	2,310.24	2,355.94
EIF3I	NC_007300:125517491..125525248	1,384.00	1,652.56	1,816.02	2,005.98	2,056.71
EIF4A1	NC_007317:27808963..27814974	9,112.00	10,709.85	10,151.68	10,380.31	10,338.16
EIF4A2	NC_007299:82284446..82290755	1,238.00	1,879.70	2,478.00	2,156.63	2,345.61
EIF4E	NC_007304:27476393..27514922	450.00	646.44	789.76	824.23	712.98
EIF4EBP1	NC_007328:35470745..35494090	228.00	130.31	150.65	101.79	174.01
EIF4G2	NC_007313:40885832..40896192	6,350.00	6,972.45	8,745.40	6,998.73	7,337.67
EIF4GI	NC_007299:84728945..84746773	3,963.00	5,109.40	5,746.08	4,728.29	4,711.89
EIF5	NC_007319:68155284..68164644	2,790.00	4,236.61	4,081.32	2,966.19	3,753.81
EIF6	NC_007311:65130218..65136103	838.00	1,072.38	1,314.21	1,659.29	1,584.16
ELAVL1	NC_007305:15165936..15211456	607.00	831.84	877.55	819.72	783.74
ELF1	NC_007310:9791229..9877139	1,693.00	1,845.07	2,062.60	1,965.22	2,275.34
ELMO2	NC_007311:75862818..75889759	799.00	1,001.61	1,155.79	1,001.77	1,197.47
ELOVL5	NC_007324:25933749..26066461	859.00	1,021.58	1,148.98	1,199.35	1,121.02
EMID1	NC_007315:71962582..72002811	276.00	87.29	150.81	104.86	143.85
EMID2	NC_007326:37355127..37519686	53.00	27.83	20.39	21.93	14.54
EMILIN2	NC_007325:38473797..38532872	2,322.00	1,971.98	1,330.92	1,235.21	1,437.66
EML2	NC_007316:52985565..53016640	370.00	646.69	559.40	750.48	784.82
ENO1	NC_007314:41637031..41650708	13,994.00	14,524.81	16,908.81	15,602.83	18,589.76
ENPP1	NC_007307:71855733..71926156	113.00	76.23	74.37	59.02	72.92
ENTHD1	NC_007303:118309422..118401028	402.00	171.41	120.52	305.02	228.86
EPAS1	NC_007309:29813353..29904884	216.00	110.91	69.94	126.84	91.54
EPB41L3	NC_007325:40368289..40461218	85.00	42.18	32.19	43.48	30.44
EPB41L4B	NC_007306:103787533..103981163	62.00	21.30	25.82	25.65	37.35
EPM2AIP1	NC_007320:10594104..10596379	92.00	145.38	140.23	135.17	155.74
EPSTI1	NC_007310:11788832..11888834	970.00	1,406.48	1,595.96	1,267.39	1,171.20
ERAP1	NC_007305:97577908..97613211	1,072.00	1,746.69	1,587.91	1,528.91	1,906.66
ETF1	NC_007305:49173869..49201887	1,175.00	1,868.00	1,885.05	1,315.54	1,515.17
EVI2A	NC_007317:18506803..18510883	709.00	496.30	592.44	422.52	433.53
EVL	NC_007319:65129949..65192725	2,682.00	1,477.30	1,489.75	1,809.20	1,887.37
EXOC6	NC_007327:14710607..14886616	300.00	369.12	364.95	368.02	372.84
EZH2	NC_007302:116300122..116333214	656.00	1,492.27	1,253.96	762.81	1,295.41
F13A1	NC_007324:49713264..49881829	1,136.00	613.38	382.36	631.07	492.05
FABP5	NC_007312:41734584..41741360	1,532.00	2,418.01	1,730.65	1,750.51	1,394.78
FADS3	NC_007330:42033685..42048606	1,143.00	816.41	840.83	927.74	1,015.52
FADS6	NC_007317:58192145..58207795	35.00	7.35	7.48	6.20	8.86
FAM117A	NC_007317:38061713..38104095	645.00	316.90	380.90	374.27	407.58
FAM119A	NC_007300:100999178..101008144	56.00	114.72	134.88	121.60	121.60
FAM128B	NC_007315:75765868..75770111	228.00	136.53	139.99	149.60	130.44
FAM131B	NC_007302:110802273..110810466	54.00	8.59	5.12	6.22	12.51
FAM20B	NC_007314:57981806..58018926	93.00	141.09	142.55	129.43	149.06
FAM3C	NC_007302:88915778..88970013	169.00	257.56	289.71	233.06	237.68
FAM49A	NC_007309:84008634..84064579	572.00	695.76	491.83	355.59	433.40

Continued on next page...

Table 1 – continued from previous page

Gene	Location	TCT1	TCT3	TCT4	TCT5	TCT6
FAM49B	NC_007312:10132033..10233191	1,713.00	2,392.75	2,073.27	1,502.99	1,465.75
FAM62A	NC_007303:61744941..61759003	2,996.00	1,596.57	2,101.69	2,020.13	2,059.09
FARP1	NC_007310:72845801..73085931	205.00	145.92	134.88	137.81	146.30
FARSA	NC_007305:10893382..10903483	465.00	604.73	599.47	627.07	612.22
FAS	NC_007327:11130700..11164554	389.00	639.22	683.42	857.50	639.45
FASLG	NC_007314:36729078..36737098	27.00	87.19	65.97	111.68	56.95
FASN	NC_007317:52171722..52189844	1,553.00	1,363.91	1,412.73	2,355.21	2,087.88
FAT	NC_007328:17768313..17894550	278.00	66.19	167.90	30.22	192.07
FBL	NC_007316:48912327..48919413	954.00	1,195.14	1,275.18	1,300.92	1,310.45
FBXL11	NC_007330:47001544..47095494	2,352.00	2,713.23	3,049.45	2,850.30	3,018.72
FBXL3	NC_007310:52557646..52569964	213.00	400.67	398.78	297.61	293.37
FBXL5	NC_007304:115372993..115410305	1,032.00	1,545.65	1,508.28	1,283.55	1,590.05
FBXO11	NC_007309:31282471..31394959	621.00	759.35	789.46	829.22	808.15
FBXO18	NC_007311:16413979..16452749	1,115.00	1,249.77	1,305.21	1,365.61	1,610.41
FBXO38	NC_007305:59240592..59297287	526.00	632.09	639.32	617.86	690.67
FBXW11	NC_007318:3703836..3836669	2,507.00	2,327.22	2,314.75	2,155.37	2,128.08
FCER1G	NC_007301:9213573..9216806	1,872.00	2,393.89	1,660.85	1,246.21	1,349.83
FCGR1A	NC_007301:22401978..22410919	280.00	136.23	129.87	78.70	81.94
FCGR2B	NC_007301:8648847..8665349	240.00	150.28	160.66	134.64	183.25
FCGR3A	NC_007301:8717462..8726183	863.00	1,088.45	1,219.70	1,162.95	1,356.92
FCGRT	NC_007316:55913155..55919312	629.00	474.78	462.40	441.21	438.51
FCRL6	NC_007301:10838550..10863767	68.00	30.20	22.09	26.67	41.29
FDFT1	NC_007306:7470094..7496546	596.00	1,194.60	1,341.68	1,732.56	1,665.86
FDPS	NC_007301:16650022..16658017	732.00	1,148.84	1,467.39	1,674.84	1,457.58
FES	NC_007319:21513721..21525626	609.00	400.21	313.41	214.91	315.96
FGD3	NC_007306:88561198..88593007	1,389.00	1,017.31	1,059.19	1,077.35	1,159.42
FGFR2	NC_007327:42064048..42171061	45.00	21.39	16.00	23.72	12.12
FGL1	NC_007328:21026116..21051080	96.00	31.72	20.57	58.32	50.54
FGL2	NC_007302:45606835..45610799	247.00	94.44	41.70	64.32	76.45
FHOD3	NC_007325:21442153..21964400	172.00	116.19	78.60	125.29	105.47
FKBP4	NC_007303:113816230..113824044	865.00	1,431.22	1,691.62	1,684.26	1,817.70
FLII	NC_007317:35426301..35438820	2,646.00	3,196.93	3,142.43	3,212.66	3,280.87
FLNB	NC_007320:43982598..44124914	1,930.00	1,628.60	1,538.73	2,287.39	2,564.06
FLOT1	NC_007324:28209124..28219210	1,444.00	1,305.67	1,118.01	1,276.31	1,315.34
FLT1	NC_007310:31580754..31752617	3,881.00	5,813.88	5,648.28	3,135.12	4,151.40
FLT3LG	NC_007316:55881354..55890835	294.00	666.33	621.80	806.52	679.54
FNBP1	NC_007309:103974159..104108282	3,047.00	3,649.62	4,090.17	3,546.37	4,148.02
FOSB	NC_007316:52856391..52863159	26.00	117.06	135.34	55.33	137.94
FOXK2	NC_007317:51493236..51573133	775.00	879.38	946.13	896.73	872.11
FOXRED2	NC_007303:80533388..80546781	48.00	18.96	25.67	25.24	25.06
FRAT1	NC_007327:20731631..20734291	89.00	49.11	51.81	54.63	49.19
FRMD5	NC_007319:55965474..56287647	192.00	123.54	82.18	138.69	131.82
FSCN1	NC_007326:40585186..40594581	649.00	468.89	475.35	487.58	516.58
FSTL4	NC_007305:43086822..43511549	141.00	95.77	82.30	97.48	102.29
FTH1	NC_007330:42118584..42121248	152,566.00	151,185.50	138,935.81	128,262.42	140,789.52
FTO	NC_007316:21434346..21904695	1,979.00	1,092.82	1,037.59	671.03	476.26
FTSJ1	NC_007312:9431605..9438040	206.00	276.15	310.12	301.17	360.83
FTSJ1	NC_007331:55857210..55865097	327.00	443.10	471.69	500.16	488.14
FUBP1	NC_007301:71229645..71253607	799.00	1,047.72	1,090.50	1,188.97	1,116.90
FUNDC1	NC_007331:62426907..62437971	128.00	194.93	218.82	188.81	217.69
FUS	NC_007326:29094108..29104133	3,359.00	4,027.71	3,735.34	4,110.46	3,675.61
FUSIP1	NC_007300:133368685..133380746	959.00	1,176.05	1,383.29	1,288.33	1,231.77

Continued on next page...

Table 1 – continued from previous page

Gene	Location	TCT1	TCT3	TCT4	TCT5	TCT6
FXR1	NC_007299:88106022..88179979	719.00	1,035.42	1,210.61	814.69	1,009.87
FYN	NC_007307:40433662..40585650	3,008.00	2,539.17	2,202.63	2,800.17	2,521.48
G0S2	NC_007314:71804284..71805192	2,058.00	2,289.09	1,743.20	645.68	746.71
G3BP1	NC_007305:62736658..62765451	993.00	1,538.73	1,634.69	1,515.10	1,394.42
G3BP2	NC_007304:93693364..93729034	830.00	1,074.08	1,244.45	1,157.37	1,185.47
GABARAP	NC_007317:27331958..27333889	1,832.00	1,506.55	1,435.86	1,302.02	1,212.61
GABARAPL1	NC_007303:107086029..107090470	517.00	887.40	621.88	719.42	695.16
GADD45B	NC_007305:19835578..19837689	374.00	597.43	659.25	653.93	674.52
GALNT3	NC_007300:31565065..31614847	296.00	360.19	438.35	378.97	434.61
GAPD	NC_007303:10853525..10857808	13,918.00	12,872.66	15,421.41	15,439.50	18,627.48
GARS	NC_007302:68318347..68380882	617.00	859.30	866.21	809.14	748.47
GAT	NC_007313:82840397..82860416	25.00	9.93	5.18	8.90	7.19
GBP4	NC_007301:57654680..57708413	2,122.00	3,772.35	4,467.76	4,314.25	4,512.76
GBP5	NC_007301:57497535..57514016	882.00	1,905.84	2,443.41	1,753.54	2,107.97
GBP6	NC_007301:57264867..57290616	666.00	1,466.25	1,921.26	1,430.28	1,640.83
GCC2	NC_007309:46531790..46565124	270.00	389.66	380.97	342.39	355.44
GCFC	NC_007299:1874808..1907647	760.00	965.14	1,119.19	982.24	1,061.97
GCLM	NC_007301:52892106..52909830	102.00	186.79	189.45	194.48	232.28
GCNT2	NC_007324:46274167..46276346	40.00	118.37	71.42	84.96	68.18
GDA	NC_007306:50707564..50835047	169.00	566.10	312.74	312.21	322.54
GDI1	NC_007331:23762869..23768632	3,384.00	4,430.95	3,777.55	3,686.09	4,150.31
GDI2	NC_007311:43225325..43253976	2,707.00	3,353.33	3,886.83	3,197.85	3,203.32
GFI1B	NC_007309:106750895..106763239	26.00	10.35	4.46	8.80	10.73
GFPT1	NC_007309:69642778..69707857	98.00	147.14	150.30	158.37	180.94
GHDC	NC_007317:43640938..43645383	292.00	190.43	214.26	190.24	193.03
GHSR	NC_007299:97145743..97149387	18.00	60.86	49.08	49.96	69.47
GIMAP1	NC_007302:117597276..117607727	2,284.00	1,560.17	1,517.34	2,062.28	1,642.89
GIMAP6	NC_007302:117308888..117313432	4,152.00	3,388.04	3,553.38	4,431.62	3,764.60
GIMAP7	NC_007302:117204738..117213577	2,271.00	1,795.87	1,763.89	2,679.86	1,796.47
GIMAP8	NC_007302:117129646..117180541	4,886.00	5,328.31	5,508.21	5,352.80	6,022.53
GINS4	NC_007328:38867201..38882692	62.00	31.03	36.50	34.46	32.49
GIT2	NC_007315:66597750..66640414	1,086.00	909.44	891.34	976.42	930.13
GLB1L3	NC_007313:84463110..84496250	245.00	119.22	168.46	137.61	133.33
GLI3	NC_007302:81736971..81949880	69.00	35.90	18.29	43.57	32.66
GLRB	NC_007315:44090800..44180230	52.00	29.10	12.85	27.38	14.63
GLS	NC_007300:83287214..83341352	932.00	1,138.34	1,193.02	1,234.62	1,447.95
GLTSCR2	NC_007316:54505640..54514304	2,030.00	1,128.84	1,366.91	1,105.15	1,152.90
GLUL	NC_007314:61002133..61012683	958.00	2,197.15	2,147.34	1,090.93	1,723.34
GMEB1	NC_007300:128926424..128953181	190.00	312.06	332.38	268.56	313.76
GMPS	NC_007299:114053499..114124656	761.00	971.99	1,358.81	1,082.93	1,049.57
GNAS	NC_007311:58181776..58195302	3,931.00	4,235.79	5,399.98	4,935.45	5,553.70
GNB1	NC_007314:48127959..48205919	4,114.00	4,752.56	4,591.92	4,761.61	4,788.24
GNB2L1	NC_007305:39717488..39722281	14,382.00	12,038.91	15,460.69	17,432.07	16,996.09
GNE	NC_007306:63254285..63288816	520.00	776.11	778.90	744.62	757.14
GNL3	NC_007320:49215979..49222371	532.00	632.98	731.05	819.49	658.73
GNLY	NC_007309:50960680..50968719	527.00	1,485.43	2,103.49	1,134.22	1,130.69
GNLY	NC_007309:51004917..51007329	643.00	1,688.02	2,448.00	1,283.79	1,320.93
GNPTAB	NC_007303:70496784..70592546	986.00	1,535.13	1,509.05	1,173.09	1,468.80
GOLGA7	NC_007328:38831999..38848858	763.00	1,003.88	1,025.32	656.89	1,026.04
GOLPH3L	NC_007301:21576064..21620314	519.00	429.76	405.00	441.64	387.78
GORASP2	NC_007300:26386889..26417067	551.00	666.64	723.36	701.98	659.05
GPBP1	NC_007318:23624161..23688918	946.00	1,261.08	1,321.97	1,112.21	1,135.31

Continued on next page...

Table 1 – continued from previous page

Gene	Location	TCT1	TCT3	TCT4	TCT5	TCT6
GPC5	NC_007310:64733901..65680319	2,943.00	1,809.62	1,543.18	2,723.90	2,145.80
GPC6	NC_007310:66633090..67140504	2,082.00	4,067.74	3,934.19	3,478.17	3,028.20
GPD2	NC_007300:41069964..41217777	842.00	1,559.51	1,237.17	1,102.58	1,415.67
GPI	NC_007316:44521762..44549890	4,181.00	4,655.32	5,267.65	3,391.34	5,539.44
GPR116	NC_007324:20788105..20862995	55.00	22.24	16.43	22.16	27.84
GPR155	NC_007300:22969574..23008483	360.00	665.84	868.67	687.81	931.52
GPR171	NC_007299:118471626..118478167	548.00	1,095.42	1,651.36	1,388.82	1,092.58
GPR35	NC_007301:125943577..125970948	857.00	401.67	615.20	396.76	637.49
GPSM3	NC_007324:27020505..27022199	1,032.00	847.25	774.83	527.02	547.73
GPX1	NC_007320:51684442..51685610	1,773.00	2,157.03	2,131.08	1,275.70	1,961.46
GPX4	NC_007305:42612802..42615223	620.00	886.57	1,080.26	895.70	1,211.37
GRAMD3	NC_007305:26241763..26317422	602.00	747.02	814.31	852.72	839.11
GRAP	NC_007317:35234109..35254981	390.00	252.20	248.27	283.29	243.03
GRIK1	NC_007299:5323513..5809203	3,181.00	2,891.98	2,112.32	2,610.09	2,430.80
GRIK2	NC_007307:49982743..50717016	816.00	677.43	589.72	567.41	510.41
GRINA	NC_007312:1059703..1060406	698.00	1,104.61	1,043.72	974.33	1,310.36
GRIPAP1	NC_007331:55422068..55444123	1,308.00	1,668.26	1,482.33	1,528.23	1,520.89
GRM1	NC_007307:86254049..86709260	325.00	219.26	145.74	261.57	196.52
GRN	NC_007317:45567704..45574694	2,473.00	2,999.87	2,191.54	1,640.45	1,901.82
GRTP1	NC_007310:84771140..84789461	865.00	1,155.85	1,150.27	1,007.17	563.12
GSG1L	NC_007326:27138638..27392535	152.00	73.37	69.03	99.42	66.48
GSN	NC_007306:116103605..116131438	3,381.00	1,506.56	1,570.34	1,328.00	1,882.09
GTDC1	NC_007300:54692760..55038282	163.00	290.40	260.12	271.54	267.22
GTF2H1	NC_007330:27595842..27625341	513.00	753.04	940.65	813.62	748.32
GTPBP1	NC_007303:117410469..117432395	627.00	737.99	743.83	725.73	815.85
GTPBP4	NC_007311:46501837..46516536	990.00	1,505.10	1,550.95	1,771.05	1,649.40
GUCY1A2	NC_007313:14581169..15099044	932.00	509.98	411.22	782.76	631.50
GYG1	NC_007299:121099445..121140175	637.00	804.00	1,109.57	938.43	760.92
GYS1	NC_007316:55363216..55379879	733.00	897.99	1,278.19	437.66	1,172.50
GYS1	NC_007316:55550114..55566768	733.00	899.63	1,278.94	438.37	1,173.96
GZMA	NC_007318:25663856..25672830	18.00	252.37	305.30	224.90	74.42
GZMB	NC_007319:34983258..34986762	21.00	128.72	131.63	104.95	62.08
H11C9ORF142	NC_007309:109296342..109297914	348.00	190.02	174.11	200.89	174.50
H13C10ORF97	NC_007311:30056918..30136656	277.00	381.65	367.26	380.15	347.70
H18C19ORF63	NC_007316:56563951..56570286	934.00	732.64	731.58	673.87	691.89
H1C21ORF91	NC_007299:18914326..18943146	190.00	329.14	361.84	313.58	386.25
H1F0	NC_007303:116686644..116688871	109.00	68.26	47.07	52.59	53.42
H22C3ORF64	NC_007320:33275865..33316225	193.00	266.26	306.52	333.71	289.73
H26C10ORF6	NC_007327:22064802..22104809	777.00	913.29	991.23	886.97	934.59
H2AFV	NC_007302:79456747..79468541	1,321.00	877.25	1,004.49	1,015.93	1,065.37
H2AFX	NC_007313:28183895..28185211	164.00	111.76	99.35	101.18	102.99
H3F3B	NC_007317:57542400..57545886	6,491.00	8,054.08	7,227.59	5,222.55	5,831.39
HACE1	NC_007307:47313184..47433054	147.00	238.12	228.38	232.06	254.86
HAT1	NC_007300:25403525..25443422	301.00	392.28	517.03	459.53	415.09
HAVCR2	NC_007305:68546326..68562055	223.00	286.40	358.64	302.41	374.56
HBB	NC_007313:47825662..47827304	288.00	15.73	55.29	37.24	179.63
HCRTR2	NC_007324:1303204..1436030	82.00	43.04	31.60	41.25	42.05
HDAC5	NC_007317:45331681..45368572	688.00	460.27	508.77	502.47	495.55
HEG1	NC_007299:70606528..70685596	590.00	814.28	961.37	786.25	1,372.96
HERC2	NC_007300:681054..911486	1,276.00	1,577.96	1,498.90	1,918.70	1,959.13
HERC3	NC_007304:36863738..37017394	462.00	571.85	605.95	619.08	679.03
HERC5	NC_007304:37076243..37120870	532.00	784.01	857.48	849.65	912.91

Continued on next page...

Table 1 – continued from previous page

Gene	Location	TCT1	TCT3	TCT4	TCT5	TCT6
HERC6	NC_007304:37128405..37185776	1,358.00	1,951.74	2,381.60	2,757.72	2,424.69
HERPUD2	NC_007302:63854836..63890895	566.00	358.24	431.64	433.62	469.79
HEXB	NC_007318:6974043..7006747	1,401.00	1,900.31	1,547.40	1,188.77	1,185.86
HGSNAT	NC_007328:40173052..40206216	1,275.00	1,108.68	962.27	796.33	1,455.66
HIC1	NC_007317:23165781..23169584	218.00	121.32	101.35	68.24	97.46
HINT1	NC_007305:21867243..21873849	520.00	729.69	720.75	687.70	612.99
HINT1	NC_007331:74396058..74396645	442.00	526.08	582.72	615.76	546.09
HIPK3	NC_007313:63581160..63661649	395.00	513.29	564.14	535.06	629.90
HIPK4	NC_007316:49375178..49389360	130.00	74.49	57.14	47.53	54.45
HISPPD1	NC_007305:106557569..106640253	1,177.00	1,622.64	1,675.41	1,573.43	1,544.40
HISPPD2A	NC_007319:55748917..55783381	61.00	110.96	105.86	133.07	107.41
HIVEP2	NC_007307:82980520..83011380	968.00	1,164.67	1,300.40	1,179.09	1,443.72
HK2	NC_007309:9999797..10122736	462.00	912.40	1,041.70	590.94	1,068.47
HMG2L1	NC_007303:78985408..79013868	173.00	240.43	253.89	294.50	246.67
HMGCS1	NC_007318:33424018..33449753	854.00	2,058.98	2,506.59	2,754.89	2,441.93
HMGN1	NC_007299:141690413..141696789	867.00	1,058.57	1,456.01	1,155.94	1,095.06
HMGN2	NC_007300:130921752..130925318	1,823.00	1,584.37	1,544.66	1,345.78	1,354.12
HMOX1	NC_007303:79089215..79096280	1,538.00	3,925.05	4,939.36	1,173.79	2,398.10
HMOX2	NC_007326:4226017..4254162	1,074.00	1,242.21	927.95	857.47	773.48
HNRNPR	NC_007300:133944328..133973387	1,167.00	1,330.07	1,486.86	1,483.04	1,333.11
HNRNPU	NC_007314:29689118..29698708	3,478.00	3,962.32	3,798.04	3,757.96	3,282.86
HNRPA1	NC_007303:28631261..28635775	3,007.00	2,660.69	2,555.26	2,401.58	2,255.64
HNRPD	NC_007304:100810949..100828074	2,924.00	3,686.34	4,004.21	4,353.53	4,543.81
HNRPDL	NC_007304:100877575..100884146	2,223.00	2,615.58	2,846.83	2,938.36	3,136.72
HNRPH1	NC_007305:1544758..1553758	4,321.00	4,893.46	5,032.28	5,281.35	5,032.38
HNT	NC_007330:36268460..36689135	247.00	346.06	193.34	149.83	162.23
HOOK3	NC_007328:40006013..40085742	382.00	479.88	461.89	295.31	545.87
HPSE2	NC_007327:19026728..19751486	491.00	297.04	233.90	412.58	311.53
HRB	NC_007300:120275199..120349837	673.00	806.23	966.62	793.74	905.25
HS2ST1	NC_007301:60454777..60491522	110.00	71.63	41.50	74.15	54.84
HS3ST5	NC_007307:37891442..38186024	208.00	99.02	99.16	116.03	89.58
HSD17B12	NC_007313:74003959..74068881	276.00	607.67	694.59	514.83	603.56
HSD17B2	NC_007316:7669386..7816100	255.00	122.79	86.47	173.08	112.53
HSD17B4	NC_007305:33415642..33576007	382.00	587.40	609.00	489.68	496.85
HSD17B7	NC_007301:7225074..7253883	249.00	348.30	348.77	434.48	417.47
HSD3B7	NC_007326:28945977..28949457	464.00	1,140.17	1,222.89	713.46	950.76
HSF1	NC_007312:448053..453004	533.00	634.81	681.92	619.51	700.75
HSP90AB1	NC_007324:18387896..18393559	11,358.00	16,353.88	17,497.62	17,064.21	15,107.94
HSPA14	NC_007311:29128108..29152707	591.00	875.29	1,005.46	854.93	895.12
HSPA1A	NC_007324:27311563..27313661	1,145.00	6,986.06	11,281.87	2,910.94	9,461.92
HSPA4	NC_007305:44324534..44378550	1,035.00	1,507.71	1,663.28	1,435.27	1,291.36
HSPA5	NC_007309:99543122..99547281	2,806.00	3,599.34	4,088.68	3,849.17	3,693.17
HSPA6	NC_007301:8748711..8750920	145.00	1,490.82	2,894.61	454.64	2,287.99
HSPA8	NC_007313:32348150..32352578	11,435.00	24,862.21	31,883.59	24,283.47	28,777.47
HSPA9	NC_007305:49209904..49225204	1,627.00	2,516.50	2,715.82	2,282.62	2,304.29
HSPB1	NC_007326:36498025..36499514	1,388.00	2,787.18	2,905.44	1,522.69	2,174.34
HSPBAP1	NC_007299:68150761..68206834	243.00	394.28	361.84	411.96	316.77
HSPCA	NC_007319:67024629..67029960	6,187.00	11,305.03	13,021.90	8,073.31	11,951.94
HSPD1	NC_007300:90231336..90250442	2,007.00	3,386.15	3,853.62	3,270.44	3,007.00
HSPE1	NC_007300:90250595..90252635	603.00	1,001.16	1,121.57	826.56	786.65
HSPG2	NC_007300:135384224..135462898	17.00	48.13	41.75	46.25	39.78
HSPH1	NC_007310:29596281..29619754	798.00	2,793.99	3,785.91	1,587.72	2,409.46

Continued on next page...

Table 1 – continued from previous page

Gene	Location	TCT1	TCT3	TCT4	TCT5	TCT6
HUWE1	NC_007331:58717874..58875199	2,857.00	3,836.77	3,975.30	4,833.61	5,159.28
HYLS1	NC_007330:30791014..30803117	352.00	509.34	495.50	424.23	432.61
HYOU1	NC_007313:28141383..28152265	973.00	1,421.48	1,856.55	1,716.09	1,612.68
IARS	NC_007306:88005764..88087293	665.00	840.02	829.79	926.38	946.79
ICAM1	NC_007305:13276811..13287375	2,885.00	8,504.49	9,601.10	4,677.31	7,113.62
ICAM2	NC_007317:49747503..49759651	859.00	1,319.06	1,238.61	1,752.80	1,258.17
ICAM3	NC_007305:13323018..13330276	485.00	646.68	597.74	729.15	670.68
ICAM4	NC_007305:13286426..13288828	973.00	2,817.50	3,428.78	1,699.30	2,491.19
ICOS	NC_007300:97020752..97044394	365.00	454.05	504.99	804.93	523.93
ID2	NC_007309:91375665..91377971	633.00	1,000.39	1,194.24	1,130.56	1,025.56
IDI1	NC_007311:46495484..46498611	317.00	602.04	793.03	734.20	685.06
IER3	NC_007324:28207551..28208818	2,223.00	2,629.28	2,612.07	1,161.79	1,817.04
IFI30	NC_007305:4987167..4990573	1,616.00	2,908.71	3,000.83	1,990.20	2,512.73
IFI44	NC_007301:70457653..70477784	324.00	411.50	485.34	526.19	400.04
IFIH1	NC_007300:35282039..35337709	889.00	1,517.79	1,429.05	1,471.10	1,337.61
IFIT3	NC_007327:11443452..11450569	120.00	230.50	249.14	256.60	205.31
IFITM3	NC_007324:27747646..27748083	895.00	547.38	674.35	1,131.01	644.89
IFNG	NC_007303:49351209..49356033	106.00	2,659.93	1,379.97	1,713.01	594.25
IGF2R	NC_007307:100048343..100150955	2,565.00	1,959.98	1,926.18	1,700.90	2,195.10
IGFBP6	NC_007303:29836176..29840021	131.00	73.57	55.63	39.97	51.44
IHPK2	NC_007320:52184299..52208059	411.00	269.53	311.12	294.28	340.24
IKBKB	NC_007328:39543845..39596306	1,522.00	2,107.05	1,834.29	2,138.74	2,853.55
IKBKE	NC_007314:3275150..3300297	1,566.00	1,268.99	1,083.53	982.94	1,097.57
IL10RA	NC_007313:27037383..27103012	824.00	981.87	967.58	962.47	1,057.82
IL11RA	NC_007306:80243221..80249712	31.00	14.46	7.38	4.40	3.59
IL12B	NC_007305:70712061..70722455	16.00	51.08	67.57	42.98	35.38
IL12RB1	NC_007305:5057292..5074184	541.00	643.04	663.58	680.03	857.57
IL12RB2	NC_007301:83639585..83713938	269.00	675.54	625.92	759.69	738.79
IL13	NC_007305:20475286..20477286	3.00	23.71	27.72	38.38	30.76
IL16	NC_007319:26923915..26940266	872.00	445.34	573.89	625.97	687.01
IL17A	NC_007324:25118586..25122111	89.00	1,341.54	670.55	991.25	680.68
IL17F	NC_007324:25158307..25165851	425.00	2,316.49	1,648.89	2,702.22	2,066.48
IL18BP	NC_007313:51135674..51137637	95.00	885.01	1,386.58	206.42	684.19
IL18RAP	NC_007309:7421552..7445333	99.00	279.49	183.79	427.44	197.39
IL19	NC_007314:3640908..3650130	14.00	96.70	44.74	86.53	43.59
IL1A	NC_007309:48187707..48198686	3,370.00	6,191.63	7,492.07	3,157.79	4,859.62
IL1B	NC_007309:48249241..48257632	47,663.00	101,563.75	83,914.28	41,598.91	63,820.17
IL1R1	NC_007309:7055423..7134729	1,791.00	1,427.02	1,257.48	1,175.28	1,273.73
IL1R2	NC_007309:6899375..6937954	228.00	91.68	75.15	139.30	102.99
IL1RN	NC_007309:48536452..48543439	3,325.00	4,894.75	6,947.41	1,315.32	2,866.43
IL22	NC_007303:49250420..49255901	17.00	462.66	292.40	559.55	209.73
IL27	NC_007326:27874794..27879832	279.00	802.00	831.47	372.08	620.45
IL27RA	NC_007305:9854150..10065694	6,159.00	5,138.81	5,334.62	4,503.08	5,250.81
IL2RA	NC_007311:16269902..16315258	2,071.00	6,297.24	6,512.56	5,376.79	5,505.16
IL2RB	NC_007303:81426249..81436969	1,101.00	1,602.63	1,496.93	1,522.28	1,336.89
IL4R	NC_007326:26616138..26666094	2,411.00	3,552.08	2,744.64	2,146.97	2,646.61
IL6	NC_007302:32756555..32760923	340.00	728.96	744.04	520.76	246.02
IL6R	NC_007301:17504725..17556956	752.00	537.90	603.15	515.13	654.91
IL7R	NC_007318:40570051..40626245	1,349.00	3,162.14	2,653.64	1,192.87	1,769.31
IL8	NC_007304:91790448..91794218	45,415.00	74,401.50	78,867.91	44,279.63	49,278.38
IL9R	NC_007326:552487..563676	680.00	364.32	493.39	386.70	556.74
ILF3	NC_007305:13596607..13624084	1,182.00	1,504.07	1,669.34	1,426.83	1,434.42

Continued on next page...

Table 1 – continued from previous page

Gene	Location	TCT1	TCT3	TCT4	TCT5	TCT6
INDO	NC_007328:37378202..37391118	4,786.00	16,003.50	14,903.98	8,469.61	15,786.94
INHBA	NC_007302:82135935..82147401	302.00	1,633.54	2,881.77	475.80	2,091.83
INSIG1	NC_007302:121359184..121371896	380.00	712.57	883.77	721.31	893.02
IPO7	NC_007313:42206076..42251721	1,052.00	1,669.84	1,923.94	1,675.62	1,858.57
IQGAP1	NC_007319:21852881..21936373	6,350.00	9,087.23	7,757.29	6,676.43	8,513.38
IRF1	NC_007305:20624219..20631298	8,446.00	13,796.54	15,386.19	13,786.31	15,933.93
IRF4	NC_007324:53251659..53264380	780.00	1,679.44	1,679.23	1,725.75	2,047.16
IRF8	NC_007316:11089559..11111233	838.00	1,191.05	1,703.00	1,155.51	1,781.62
ISG15	NC_007314:48718794..48719821	632.00	1,746.94	1,822.82	1,886.01	1,526.47
ISG20	NC_007319:19234718..19251948	532.00	803.38	667.22	783.40	786.75
ITGA6	NC_007300:24966035..25051812	317.00	257.51	252.09	401.35	397.08
ITGB8	NC_007302:30314718..30405951	639.00	1,705.34	989.85	765.58	1,139.74
ITIH2	NC_007311:14923434..14956118	83.00	52.86	31.51	53.03	44.56
ITK	NC_007305:68633477..68695569	1,281.00	1,881.68	2,224.63	2,017.83	2,245.84
JAG1	NC_007311:3912834..3957827	2,296.00	4,325.37	4,362.11	2,859.30	4,059.45
JARID1A	NC_007303:114135323..114197350	5,289.00	4,294.42	4,395.22	4,848.03	4,734.20
JOSD3	NC_007330:654516..660465	133.00	205.52	214.03	218.65	191.65
JSP.1	NC_007324:28598863..28602504	14,665.00	16,609.28	20,990.06	19,524.47	26,966.46
JUN	NC_007301:93618853..93620900	1,812.00	1,499.25	1,545.04	835.79	1,396.28
KCND2	NC_007302:87712550..88289675	223.00	135.80	99.50	157.68	136.21
KCNIP3	NC_007309:2288163..2416601	231.00	115.34	125.00	130.51	158.77
KCNMB2	NC_007299:90497302..90533714	119.00	228.00	79.87	49.96	78.57
KCTD10	NC_007315:66989274..67018624	840.00	663.24	687.11	727.98	704.61
KCTD12	NC_007310:52443570..52445112	3,443.00	3,034.73	3,780.88	2,100.83	3,117.36
KCTD15	NC_007316:43672614..43687280	64.00	11.46	33.08	20.57	38.48
KDELR3	NC_007303:117224182..117237342	137.00	97.84	94.14	75.73	77.83
KEAP1	NC_007305:13468633..13477969	728.00	932.17	1,117.52	983.97	1,089.85
KHDRBS2	NC_007324:5221303..5747469	903.00	463.44	373.51	653.85	447.16
KIAA0174	NC_007316:38258757..38298043	960.00	1,081.36	1,099.24	1,075.35	1,240.02
KIAA0859	NC_007314:35801862..35818136	295.00	431.98	508.78	488.14	488.67
KIF13B	NC_007306:9480415..9693186	489.00	916.87	815.78	672.80	811.74
KIF3B	NC_007311:62607690..62649539	358.00	516.03	575.19	495.99	658.01
KIF5B	NC_007311:33234958..33281214	1,000.00	1,782.83	1,775.99	1,310.14	1,530.05
KIRREL3	NC_007330:31383190..31979139	218.00	97.48	108.81	145.48	147.12
KIT	NC_007304:72779241..72828532	365.00	260.23	229.54	269.60	438.54
KLF11	NC_007309:90321942..90349079	270.00	130.13	171.88	184.03	157.34
KLF6	NC_007311:44752631..44759731	962.00	1,415.51	1,557.25	1,149.43	1,672.47
KLHL5	NC_007304:60633221..60707436	270.00	477.37	509.21	538.29	580.94
KLHL8	NC_007304:106135407..106194188	434.00	1,046.73	708.85	708.04	980.81
KPNA1	NC_007299:67961696..68030299	735.00	1,006.28	1,234.67	886.17	1,080.17
KPNA3	NC_007310:18628264..18726222	476.00	573.80	653.47	598.14	629.42
KPNA6	NC_007300:125569933..125584600	375.00	515.02	534.69	449.09	509.67
KPNB1	NC_007317:40017871..40040128	1,966.00	2,400.64	2,536.39	2,716.39	2,533.43
KRAS	NC_007303:91172985..91212523	202.00	304.90	288.12	265.75	260.52
KRIT1	NC_007302:9770599..9809812	201.00	281.75	274.35	299.45	280.07
KSR2	NC_007315:60551030..60745671	22.00	5.22	2.03	6.68	7.92
KTELC1	NC_007299:65221796..65244847	84.00	124.28	140.50	124.51	133.99
KYNU	NC_007300:55971967..56156355	258.00	361.00	382.15	375.98	379.06
L1CAM	NC_007331:23379678..23393995	1,723.00	566.79	892.32	566.44	1,026.06
LAMA4	NC_007307:39976214..40136618	304.00	156.39	174.29	214.62	191.66
LAMP2	NC_007331:422236..450542	690.00	813.38	1,086.02	783.77	809.11
LAP3	NC_007304:37961716..37987281	4,621.00	8,901.76	12,092.66	11,041.77	13,042.00

Continued on next page...

Table 1 – continued from previous page

Gene	Location	TCT1	TCT3	TCT4	TCT5	TCT6
LARP1	NC_007305:65732328..65829601	2,111.00	3,451.41	3,183.62	2,970.33	3,319.54
LARP4	NC_007303:32442909..32512473	475.00	678.07	678.22	569.37	598.87
LARP5	NC_007311:46592704..46649218	807.00	1,101.05	1,205.30	1,090.69	1,149.73
LARS	NC_007305:57363516..57445594	622.00	805.39	932.13	891.10	867.57
LASS3	NC_007319:4789730..4957299	973.00	437.20	308.54	469.17	415.70
LAT	NC_007326:27675446..27680366	1,566.00	2,562.86	2,474.80	2,107.50	2,145.95
LBH	NC_007309:71570000..71595738	1,519.00	1,092.08	1,171.91	1,723.70	1,276.59
LBR	NC_007314:25467628..25494083	1,113.00	625.38	925.06	874.22	817.02
LCK	NC_007300:125481821..125502755	977.00	871.21	677.60	1,615.83	1,164.97
LCP2	NC_007318:970108..1017063	1,628.00	1,865.55	1,881.81	1,921.58	2,199.00
LDHA	NC_007331:39935216..39936956	6,827.00	6,461.67	7,996.80	4,311.87	5,660.05
LDLR	NC_007305:13997825..14031492	800.00	1,386.40	1,288.17	1,303.26	1,623.62
LEF1	NC_007304:18483969..18600964	1,035.00	740.09	707.97	2,227.60	1,558.93
LGALS1	NC_007303:116532588..116535544	883.00	1,605.67	1,560.40	1,820.99	1,764.94
LIF	NC_007315:72761168..72765481	32.00	465.54	363.84	627.13	293.18
LIG1	NC_007316:54715278..54742532	290.00	150.62	169.17	171.66	181.50
LIG4	NC_007310:81640254..81644474	105.00	170.66	147.45	148.80	151.64
LILRA2	NC_007316:63252872..63257342	783.00	1,026.51	688.72	562.50	662.12
LIMD2	NC_007317:49517186..49518963	1,797.00	1,014.66	1,254.25	1,575.66	1,089.83
LIPG	NC_007325:50881050..50967976	245.00	582.52	425.18	389.87	480.63
LMAN2	NC_007305:38138806..38159433	1,031.00	1,281.51	1,319.36	1,379.25	1,376.73
LMNA	NC_007301:15980698..15997687	1,235.00	2,233.08	1,606.63	1,491.42	2,238.96
LMNB1	NC_007305:26057876..26107892	639.00	1,127.36	1,075.93	930.24	892.95
LOC100124428	NC_007306:65647411..65684697	522.00	717.52	784.80	834.77	884.29
LOC100125414	NC_007326:28290625..28293318	882.00	653.58	744.40	674.72	668.23
LOC100137734	NC_007326:38236373..38254633	300.00	173.72	185.90	108.19	168.96
LOC100137803	NC_007314:46925117..47162092	44.00	11.10	12.13	22.19	14.77
LOC100137897	NC_007317:19127498..19195454	100.00	47.06	36.73	47.56	41.14
LOC100138139	NC_007311:65456751..65460443	354.00	533.95	559.89	448.68	465.46
LOC100138178	NC_007317:38797498..38817277	1,821.00	2,446.22	2,386.75	2,537.18	2,535.33
LOC100138193	NC_007304:12525501..12612387	223.00	129.29	88.83	149.78	90.54
LOC100138230	NC_007319:55891108..55893382	421.00	587.37	566.93	525.39	517.53
LOC100138254	NC_007324:18463213..18506994	49.00	13.63	17.70	27.71	18.75
LOC100138311	NC_007327:18372508..18481594	6,223.00	7,315.04	7,172.50	5,446.92	7,462.44
LOC100138312	NC_007300:115532213..115608198	6,896.00	5,538.49	5,443.24	4,744.53	4,527.76
LOC100138341	NC_007311:31475938..31499495	5,833.00	9,370.15	8,536.98	7,223.71	9,274.99
LOC100138376	NC_007314:65488376..65519837	2,430.00	3,250.54	6,542.87	2,192.21	4,538.63
LOC100138453	NC_007301:82017186..82132930	121.00	59.69	48.87	82.60	85.17
LOC100138550	NC_007304:71571085..71637427	575.00	705.28	776.57	829.68	785.59
LOC100138555	NC_007300:122453452..122467144	1,893.00	2,527.78	2,678.27	2,707.96	2,504.83
LOC100138615	NC_007314:52467488..52468110	135.00	179.13	193.35	215.23	196.17
LOC100138627	NC_007324:22909117..22910877	22,671.00	18,965.94	17,229.65	20,251.59	19,102.83
LOC100138708	NC_007317:28076068..28104387	2,778.00	2,993.99	2,513.26	3,082.39	3,326.44
LOC100138789	NC_007317:58343974..58374557	112.00	62.50	49.65	32.40	42.40
LOC100138864	NC_007311:14968437..14970206	937.00	1,293.67	1,585.16	1,541.82	1,518.91
LOC100138898	NC_007304:97054451..97060589	316.00	444.22	510.09	386.70	539.45
LOC100138911	NC_007301:93619159..93620427	1,443.00	1,217.66	1,251.16	693.35	1,173.03
LOC100138951	NC_007316:57094461..57120384	428.00	552.55	534.58	303.60	630.18
LOC100139007	NC_007309:42896735..42900556	120.00	72.46	83.34	70.38	80.10
LOC100139009	NC_007306:42064821..42065358	1,033.00	722.85	854.87	910.25	797.37
LOC100139023	NC_007303:21194562..21235154	54.00	17.44	12.12	30.85	18.47
LOC100139030	NC_007320:51684787..51693732	1,533.00	1,912.01	1,863.64	1,109.55	1,734.86

Continued on next page...

Table 1 – continued from previous page

Gene	Location	TCT1	TCT3	TCT4	TCT5	TCT6
LOC100139053	NC_007304:101527853..101593627	245.00	406.09	475.79	461.13	431.94
LOC100139125	NC_007299:85699788..85899479	658.00	756.00	841.08	810.85	816.33
LOC100139189	NC_007301:118882335..119566631	190.00	104.63	77.65	127.48	110.33
LOC100139246	NC_007320:54686695..54766475	696.00	1,142.63	970.90	1,436.22	1,068.42
LOC100139249	NC_007301:21610167..21610668	317.00	242.97	218.23	220.49	178.08
LOC100139388	NC_007305:17173464..17174737	1,264.00	1,073.55	874.95	766.76	583.00
LOC100139419	NC_007305:38419641..38421407	35,979.00	39,014.48	41,463.28	42,582.38	44,206.15
LOC100139422	NC_007320:26749425..27106244	376.00	297.30	272.80	299.21	274.30
LOC100139504	NC_007299:7976286..7978919	145.00	90.00	99.12	50.73	67.82
LOC100139670	NC_007327:11465212..11471247	171.00	255.85	272.76	248.70	282.30
LOC100139700	NC_007307:106559712..106710843	143.00	82.52	75.12	74.45	61.80
LOC100139815	NC_007317:8464869..8751272	2,531.00	2,269.02	2,235.07	2,258.03	2,193.58
LOC100139910	NC_007320:52015195..52084908	4,151.00	3,565.92	3,757.17	3,373.01	2,903.60
LOC100139916	NC_007326:3048911..3065080	1,312.00	446.96	1,029.92	654.25	817.80
LOC100139996	NC_007316:59153435..59233114	127.00	74.49	79.70	85.12	83.49
LOC100140016	NC_007331:83027995..83662139	230.00	311.58	289.33	304.41	338.80
LOC100140040	NC_007315:54745164..54754756	1,373.00	1,648.03	1,773.22	1,519.40	1,252.57
LOC100140050	NC_007317:24491896..24535477	719.00	441.16	409.47	451.47	491.75
LOC100140149	NC_007326:672689..673498	434.00	6.75	47.85	14.62	249.29
LOC100140159	NC_007303:110233847..110292353	394.00	203.27	259.59	207.55	291.52
LOC100140226	NC_007316:59927608..59994397	16,694.00	21,560.85	27,965.31	41,204.67	9,666.80
LOC100140254	NC_007299:83822844..83834236	1,330.00	1,473.42	1,948.72	1,690.87	1,677.32
LOC100140276	NC_007317:28037404..28046871	1,272.00	1,928.60	1,598.42	1,483.98	1,915.74
LOC100140422	NC_007311:17453009..17454791	33.00	81.53	60.31	68.53	69.22
LOC100140540	NC_007324:17153313..17154371	120.00	73.89	84.06	75.11	56.25
LOC100140583	NC_007317:56136529..56244615	4,691.00	3,169.72	3,209.09	3,345.68	3,448.59
LOC100140676	NC_007307:7009528..7011280	8,321.00	7,307.08	7,146.54	9,079.27	9,429.00
LOC100140763	NC_007302:45522262..45651081	388.00	227.90	147.05	204.02	187.64
LOC100140797	NC_007325:34198332..34221061	843.00	1,201.65	1,327.93	1,092.23	1,303.16
LOC100140873	NC_007317:49778082..49789525	218.00	419.10	329.21	574.85	591.75
LOC100140923	NC_007313:50870685..50901755	677.00	917.30	1,197.38	1,236.07	1,263.79
LOC100141095	NC_007301:61329473..61332648	32.00	61.09	66.27	55.29	58.72
LOC100141140	NC_007325:64011760..64217445	345.00	641.41	474.58	706.78	557.08
LOC100141166	NC_007304:84333510..84437220	75.00	35.64	34.30	118.01	116.94
LOC100141199	NC_007324:20677853..20691258	60.00	29.41	30.97	30.17	34.09
LOC100141266	NC_007303:33586246..33593836	4,832.00	3,455.56	3,834.67	4,401.37	4,109.61
LOC100141269	NC_007317:18455926..18662759	2,602.00	2,249.91	2,412.60	2,150.50	2,223.95
LOC100141294	NC_007300:122772609..122797026	217.00	319.28	366.09	296.16	389.57
LOC504406	NC_007316:38964979..39288203	1,159.00	525.54	377.44	700.82	557.57
LOC504800	NC_007326:27870219..27874659	1,676.00	2,183.36	1,025.22	998.56	989.53
LOC504806	NC_007310:52497446..52516156	1,640.00	4,522.00	3,929.17	3,201.09	2,612.91
LOC504861	NC_007316:60944626..60950509	1,318.00	2,663.69	2,656.38	1,950.68	2,265.87
LOC504909	NC_007317:63264847..63314490	131.00	36.76	32.65	32.40	42.40
LOC504927	NC_007301:136079..147630	62.00	14.75	8.70	29.92	21.94
LOC505265	NC_007313:47282278..47301358	514.00	966.97	782.71	1,057.99	774.40
LOC505632	NC_007300:74122038..74162108	169.00	40.91	52.99	52.97	50.29
LOC505766	NC_007304:96943204..97055603	636.00	745.06	844.29	770.67	987.42
LOC505800	NC_007326:3050605..3053334	824.00	242.88	626.74	389.63	459.11
LOC505851	NC_007324:30554448..30559659	972.00	1,173.67	776.73	814.55	817.04
LOC505984	NC_007303:121736828..121752850	425.00	232.85	275.28	296.60	309.99
LOC506005	NC_007306:41116507..41258492	815.00	1,269.93	1,400.69	1,075.15	1,458.26
LOC506185	NC_007317:51632738..51649419	161.00	75.00	82.98	90.98	88.60

Continued on next page...

Table 1 – continued from previous page

Gene	Location	TCT1	TCT3	TCT4	TCT5	TCT6
LOC506315	NC_007314:35874662..36531463	471.00	393.93	295.71	367.59	288.28
LOC506412	NC_007330:27726616..27730059	14,605.00	7,721.34	7,167.25	7,968.98	7,827.52
LOC506431	NC_007314:70689099..71025732	120.00	76.88	51.56	74.70	48.52
LOC506759	NC_007301:11672470..11717039	1,372.00	1,702.19	1,676.25	2,057.28	1,646.63
LOC506812	NC_007330:47979661..48019687	720.00	510.96	571.94	601.94	582.91
LOC506831	NC_007312:819149..836718	83.00	47.99	45.85	34.34	52.29
LOC507126	NC_007300:129671631..129684628	713.00	855.16	550.75	508.02	605.00
LOC507141	NC_007300:131064696..131066742	2,402.00	1,162.41	1,796.75	2,050.74	1,404.42
LOC507340	NC_007313:76926160..76967921	417.00	255.82	331.74	330.27	330.52
LOC507402	NC_007305:5676527..5680537	172.00	476.12	505.91	383.00	326.88
LOC507479	NC_007311:65290227..65330009	551.00	417.49	452.77	429.91	436.82
LOC507708	NC_007320:6145805..6473546	236.00	138.03	147.13	174.60	138.90
LOC507743	NC_007311:52042065..52043251	458.00	682.67	573.39	655.07	627.77
LOC507891	NC_007319:2092647..2144652	65.00	21.45	24.38	37.48	32.78
LOC508015	NC_007315:74638049..74646401	360.00	182.78	192.07	247.11	220.65
LOC508226	NC_007319:67776697..67845256	2,209.00	1,763.59	1,579.59	1,298.18	1,461.91
LOC508347	NC_007301:70489612..70507482	371.00	551.25	588.43	691.40	538.12
LOC508527	NC_007304:113405029..113458478	805.00	1,058.33	926.96	1,078.40	1,201.50
LOC508529	NC_007327:32420223..32440555	1,697.00	1,248.35	1,299.80	1,426.78	1,392.26
LOC508666	NC_007317:13907127..13912306	356.00	207.34	47.61	100.63	99.36
LOC508755	NC_007310:70260816..70301563	174.00	342.96	245.47	385.57	416.05
LOC508858	NC_007319:34880915..34882982	9.00	30.33	23.68	24.36	48.82
LOC509375	NC_007300:103253918..103255064	578.00	443.62	312.30	334.95	327.27
LOC509490	NC_007305:50932884..51044707	1,139.00	1,569.74	1,550.66	1,518.17	1,575.93
LOC509695	NC_007310:20298690..20369514	499.00	730.44	726.35	722.02	657.61
LOC509859	NC_007320:10688873..10729793	2,921.00	3,438.51	3,243.75	3,644.06	3,682.29
LOC510320	NC_007302:111133467..111150640	441.00	1,422.81	778.42	624.49	785.31
LOC510382	NC_007301:57863895..57885800	349.00	101.82	2,667.00	442.94	2,531.25
LOC510442	NC_007305:20061170..20113621	1,162.00	1,965.59	1,824.41	1,411.48	1,964.34
LOC510487	NC_007303:70106434..70192477	367.00	441.43	477.82	563.76	447.83
LOC510604	NC_007330:13078291..13100389	477.00	723.80	802.16	754.39	787.28
LOC510634	NC_007317:57367063..57387843	209.00	100.73	123.11	113.04	84.81
LOC511059	NC_007304:69758873..70028776	930.00	1,388.18	1,481.90	1,752.85	1,823.94
LOC511121	NC_007313:39674232..39780260	904.00	1,128.41	1,219.85	1,130.84	1,168.79
LOC511531	NC_007301:58297947..58310494	2,986.00	4,736.51	7,630.81	4,846.94	6,450.39
LOC511659	NC_007316:7490317..7542429	815.00	952.84	954.13	1,076.77	1,165.97
LOC511901	NC_007320:60977065..60978481	1,138.00	615.83	645.23	561.11	643.30
LOC512010	NC_007304:57711220..57800938	298.00	517.15	508.33	472.03	618.67
LOC512486	NC_007301:57551570..57567793	4,458.00	6,395.17	7,022.30	6,302.59	6,207.14
LOC512529	NC_007303:111223127..111560377	198.00	113.77	103.41	117.10	110.14
LOC512672	NC_007324:28428431..28440307	3,315.00	3,851.73	3,814.77	4,051.80	3,755.11
LOC512863	NC_007316:57557137..57560847	29.00	7.37	4.51	5.67	7.99
LOC512869	NC_007317:53773521..53873714	750.00	935.99	994.42	1,235.16	868.32
LOC513129	NC_007311:24498041..24909553	254.00	147.82	121.52	158.26	116.96
LOC513508	NC_007315:58250614..58289073	179.00	113.40	85.21	116.88	123.73
LOC513555	NC_007306:106018620..106141764	1,212.00	1,880.16	2,012.90	1,576.45	1,806.66
LOC513653	NC_007299:58839784..58946404	640.00	516.00	475.39	543.38	460.68
LOC513842	NC_007304:224147..225123	54,691.00	60,767.50	46,032.15	42,701.98	47,475.19
LOC513955	NC_007310:20266444..20297941	43.00	78.32	78.75	74.03	72.39
LOC513969	NC_007311:16598847..16673358	190.00	114.67	95.42	137.05	113.78
LOC514143	NC_007301:57607852..57631551	796.00	1,660.06	1,810.13	1,780.20	1,330.47
LOC514170	NC_007299:147696997..147701236	4,019.00	5,900.54	5,581.53	3,503.94	4,520.83

Continued on next page...

Table 1 – continued from previous page

Gene	Location	TCT1	TCT3	TCT4	TCT5	TCT6
LOC514182	NC_007304:2487310..2537641	262.00	399.46	386.09	411.21	436.65
LOC514282	NC_007303:79905240..79928822	2,738.00	1,516.17	1,954.78	1,775.41	2,187.70
LOC514296	NC_007320:49469848..49473338	514.00	367.54	278.93	409.75	375.31
LOC514413	NC_007305:42547837..42555564	1,394.00	1,698.34	1,575.78	1,706.98	1,867.43
LOC514455	NC_007310:74111524..74164777	1,571.00	1,967.09	2,064.28	1,906.58	1,863.10
LOC515204	NC_007300:122334973..122386114	1,566.00	2,065.70	2,164.45	1,875.43	1,905.08
LOC515527	NC_007316:62551296..62558607	376.00	916.82	724.81	471.00	713.67
LOC515570	NC_007326:15586442..15654023	903.00	1,076.08	1,088.76	1,382.34	1,132.87
LOC515651	NC_007315:76063455..76067836	191.00	114.89	110.23	139.58	111.54
LOC515679	NC_007306:10815095..10855357	40.00	10.65	20.65	16.86	20.11
LOC515828	NC_007314:2807034..2829922	2,627.00	1,540.86	2,173.12	1,542.83	2,275.29
LOC516469	NC_007311:25350650..25386705	494.00	289.37	205.23	602.04	404.99
LOC516576	NC_007314:25107723..25385000	1,000.00	773.60	672.56	661.21	487.96
LOC516921	NC_007309:38883237..39224485	447.00	282.45	188.99	362.92	282.49
LOC517359	NC_007326:27718803..27725385	568.00	669.60	855.00	747.09	658.54
LOC517756	NC_007316:54831205..54846465	5,766.00	4,459.68	4,615.02	4,802.21	4,446.08
LOC518495	NC_007303:80065516..80079732	1,344.00	719.02	915.70	884.90	962.27
LOC518878	NC_007317:52058195..52110004	210.00	88.97	88.42	111.66	146.83
LOC519047	NC_007319:26539008..26630235	24.00	7.40	6.96	8.12	7.67
LOC519803	NC_007313:41201459..41243385	1,402.00	2,166.57	1,846.18	1,185.07	2,189.89
LOC520387	NC_007303:94052667..94143422	567.00	414.88	439.31	457.18	430.68
LOC520588	NC_007314:36599143..36767142	561.00	837.80	862.12	815.05	741.09
LOC522241	NC_007301:122480002..123141971	729.00	132.72	101.08	433.18	315.52
LOC523257	NC_007315:60032274..60073524	113.00	64.16	67.63	78.81	67.66
LOC523504	NC_007305:109220359..109388676	239.00	330.18	319.66	346.92	296.11
LOC523576	NC_007303:109640129..109856019	828.00	362.55	634.71	473.30	724.84
LOC524507	NC_007307:43904548..43913286	4,435.00	3,071.99	3,430.16	3,043.64	2,858.28
LOC524654	NC_007301:110562932..110623772	88.00	38.62	48.20	59.19	49.04
LOC524939	NC_007312:757109..768372	307.00	239.95	230.09	203.16	418.36
LOC525143	NC_007326:17505175..17604782	2,236.00	2,949.94	3,032.51	2,780.03	3,263.59
LOC525507	NC_007301:35411941..35413994	318.00	406.59	409.02	554.81	476.18
LOC525624	NC_007310:25486367..26165150	1,657.00	1,178.11	1,076.54	1,499.02	1,440.95
LOC525842	NC_007305:17207829..17284564	2,787.00	4,216.67	3,443.51	3,107.72	3,339.53
LOC525919	NC_007328:3777764..4194250	203.00	106.87	113.48	125.12	143.69
LOC525938	NC_007330:47369161..47372355	179.00	69.05	125.00	95.82	107.36
LOC527137	NC_007318:33732224..33732802	470.00	386.86	167.21	185.94	243.63
LOC527664	NC_007313:78224538..78254113	956.00	1,517.51	1,505.80	1,233.64	1,555.19
LOC527744	NC_007315:55476525..55477755	230.00	721.43	569.43	476.86	544.30
LOC528166	NC_007317:26335571..26365252	15.00	2.21	1.36	2.76	4.43
LOC529125	NC_007314:20734336..20742313	169.00	332.90	312.40	269.22	291.09
LOC529196	NC_007320:54559739..54566303	156.00	262.18	242.15	204.17	253.25
LOC529294	NC_007311:55783424..55794065	520.00	661.17	708.41	705.65	647.67
LOC529366	NC_007320:44174159..44320739	4,787.00	4,060.87	4,433.05	3,491.99	2,698.05
LOC529423	NC_007302:96573427..96626909	226.00	136.58	134.61	111.34	128.57
LOC529618	NC_007305:64577779..64946763	257.00	135.90	88.36	199.56	170.75
LOC529930	NC_007314:16476053..16664031	382.00	512.83	200.93	707.81	312.18
LOC530483	NC_007299:2191297..2268376	482.00	566.57	606.27	661.75	598.81
LOC530932	NC_007316:65763868..65764312	431.00	337.26	342.10	348.28	302.75
LOC530961	NC_007300:108022458..108115439	168.00	97.87	70.65	114.88	85.57
LOC531459	NC_007328:41012974..41391938	379.00	186.16	129.41	218.43	217.34
LOC531679	NC_007320:1062518..1063162	495.00	313.63	376.25	320.33	268.68
LOC531964	NC_007304:101430077..101490752	1,206.00	1,554.76	1,751.02	1,709.28	2,054.05

Continued on next page...

Table 1 – continued from previous page

Gene	Location	TCT1	TCT3	TCT4	TCT5	TCT6
LOC532244	NC_007303:116179999..116255175	234.00	106.18	106.97	129.02	148.40
LOC532845	NC_007310:14563907..14623022	352.00	441.07	509.37	423.68	480.82
LOC533126	NC_007302:111168251..111205979	235.00	138.14	149.98	116.18	169.57
LOC533206	NC_007304:61266410..61394604	1,028.00	1,261.38	1,470.47	1,470.54	1,593.82
LOC533324	NC_007300:93898331..93955756	99.00	148.41	158.96	153.68	165.14
LOC533444	NC_007305:65884839..65924483	158.00	239.88	265.95	270.17	237.66
LOC533818	NC_007301:58115305..58135887	570.00	1,070.19	1,192.92	1,326.86	1,088.51
LOC533821	NC_007328:26423677..26618185	300.00	152.37	222.98	164.89	209.40
LOC533894	NC_007303:60740155..60820955	8,534.00	9,537.69	6,476.22	6,784.01	7,667.85
LOC533901	NC_007327:13774815..13837908	805.00	994.88	917.32	930.10	976.65
LOC533925	NC_007307:6683317..7535791	8,546.00	7,506.70	7,265.24	9,270.42	9,582.47
LOC534002	NC_007326:2842196..2845079	1,900.00	2,271.91	2,239.48	2,566.57	2,506.04
LOC534011	NC_007301:92553830..93058818	517.00	242.27	166.69	373.34	307.13
LOC534095	NC_007305:96118279..96207410	443.00	572.80	602.94	613.25	547.62
LOC534358	NC_007307:84470258..85061089	873.00	550.90	613.01	527.84	664.57
LOC534471	NC_007317:10418723..10473196	231.00	343.37	324.45	364.62	361.38
LOC534734	NC_007305:10142412..10148831	212.00	269.98	305.80	422.91	373.02
LOC534778	NC_007327:10335208..10615069	244.00	172.68	129.52	182.57	146.14
LOC534929	NC_007307:72981241..73011195	80.00	28.29	18.78	40.24	52.08
LOC535090	NC_007299:108728347..108789135	611.00	824.57	1,021.03	743.21	905.66
LOC535156	NC_007315:66321181..66414579	406.00	481.20	312.44	510.25	320.00
LOC535225	NC_007326:35312874..35316246	48.00	119.75	109.03	80.41	121.18
LOC535378	NC_007300:98192594..98965618	1,535.00	1,206.53	1,092.66	1,356.97	1,167.22
LOC535434	NC_007328:15765864..15952688	69.00	29.65	24.55	24.92	29.60
LOC535649	NC_007313:77829686..77860299	1,861.00	2,980.39	2,868.19	2,753.03	3,310.77
LOC535754	NC_007300:4461750..4557205	461.00	602.46	585.27	706.46	657.55
LOC535982	NC_007317:10940777..10950195	16.00	1.47	1.36	4.14	1.90
LOC536367	NC_007304:32725475..34072833	984.00	697.82	717.64	1,147.39	768.62
LOC536906	NC_007303:12988011..13456057	580.00	440.67	435.74	474.71	390.45
LOC536993	NC_007301:89898584..90307617	188.00	132.02	89.70	143.09	110.98
LOC537131	NC_007314:62136905..62239602	1,131.00	1,505.98	1,513.45	1,526.56	1,698.74
LOC537528	NC_007319:58390164..58636918	113.00	51.78	50.15	79.34	64.87
LOC537555	NC_007302:43275460..44562865	788.00	541.65	373.12	634.18	508.02
LOC537614	NC_007315:10410496..10838835	426.00	237.24	200.18	324.58	259.66
LOC537748	NC_007319:66935331..66996792	4,773.00	5,731.67	5,578.73	5,454.47	7,935.87
LOC537932	NC_007300:27105777..27310618	653.00	1,016.41	910.42	1,058.57	754.38
LOC538255	NC_007318:30951612..31402729	318.00	178.32	120.18	250.88	193.78
LOC538481	NC_007304:21381359..21426293	533.00	700.31	683.35	816.06	903.48
LOC538580	NC_007299:54993496..55017633	242.00	152.57	130.88	178.31	171.21
LOC539274	NC_007306:108883478..108924565	2,162.00	1,514.17	1,529.77	1,814.64	1,793.01
LOC539421	NC_007300:117416542..117654248	2,771.00	3,484.02	3,661.75	3,422.63	3,756.87
LOC539596	NC_007305:46619714..46703809	629.00	509.87	334.39	310.08	791.43
LOC539789	NC_007318:23538322..23539272	40.00	19.65	10.45	13.67	10.53
LOC540132	NC_007303:113427312..113616848	64.00	27.30	24.96	39.27	31.66
LOC540380	NC_007328:27678826..27683530	254.00	335.78	399.51	177.08	324.20
LOC540778	NC_007301:107726273..107893596	160.00	86.37	69.62	116.43	96.78
LOC540799	NC_007304:20648821..20651599	469.00	653.07	591.00	609.09	641.30
LOC541079	NC_007320:50355792..50399599	889.00	1,002.97	1,047.53	1,135.60	1,159.62
LOC541084	NC_007300:49892798..50157352	2,870.00	2,587.14	2,471.14	2,685.58	2,370.47
LOC613364	NC_007331:62296319..62297635	163.00	104.90	78.61	82.46	72.81
LOC613560	NC_007324:17405932..17408342	115.00	69.59	56.25	68.96	55.11
LOC613595	NC_007299:85679920..85698922	668.00	770.57	989.28	871.57	916.41

Continued on next page...

Table 1 – continued from previous page

Gene	Location	TCT1	TCT3	TCT4	TCT5	TCT6
LOC613607	NC_007317:27547893..27553542	710.00	483.80	503.33	534.18	529.72
LOC613633	NC_007316:47664054..47670555	71.00	22.10	21.80	18.44	20.33
LOC613670	NC_007319:3049577..3828379	1,016.00	619.92	687.42	814.02	684.92
LOC613747	NC_007309:101739696..101792824	3,015.00	2,279.53	2,213.62	2,165.80	2,709.09
LOC613970	NC_007331:72933906..72934801	8,610.00	6,375.56	7,991.13	9,593.25	7,949.93
LOC614048	NC_007305:9148714..9235070	1,627.00	2,286.65	2,004.60	2,042.39	2,267.38
LOC614067	NC_007309:97390372..97397307	1,091.00	952.33	819.97	557.21	436.97
LOC614107	NC_007309:9887265..9902110	559.00	1,049.19	1,290.97	687.72	1,198.64
LOC614129	NC_007299:53542263..53544177	342.00	507.43	738.11	574.13	601.29
LOC614198	NC_007316:46005025..46014151	296.00	360.18	535.34	497.25	582.98
LOC614219	NC_007309:48949467..48950348	5,328.00	4,087.33	5,068.62	4,497.30	3,724.64
LOC614643	NC_007301:22976394..22993592	3,833.00	2,943.73	3,371.24	4,060.58	3,329.40
LOC614724	NC_007300:132733947..132748298	53.00	15.24	16.06	15.67	18.01
LOC614732	NC_007306:90957289..90959217	575.00	741.11	787.01	773.20	765.77
LOC614844	NC_007303:81368586..81410655	29.00	97.08	74.88	94.66	63.32
LOC615030	NC_007311:16333267..16403494	248.00	517.83	629.20	395.65	550.60
LOC615147	NC_007302:45420749..45519688	611.00	1,042.72	760.13	820.21	746.39
LOC615152	NC_007312:32515051..32874775	417.00	282.04	191.74	334.08	293.60
LOC615482	NC_007301:32605079..32625555	407.00	255.61	208.86	189.27	190.99
LOC615557	NC_007327:45082931..45137414	398.00	212.28	271.24	252.29	290.86
LOC615610	NC_007300:123634186..123635532	487.00	397.90	411.86	296.16	319.01
LOC615819	NC_007306:3714583..5345935	906.00	559.25	407.89	801.05	630.71
LOC615883	NC_007318:2012948..2729718	389.00	196.45	167.21	216.02	203.61
LOC615936	NC_007326:31672853..32623031	353.00	255.53	203.16	227.34	207.74
LOC616035	NC_007330:27708473..27711865	8,002.00	4,254.25	3,550.35	3,810.27	3,728.87
LOC616165	NC_007299:41867450..42921818	6,247.00	5,264.54	4,617.27	5,502.25	5,002.19
LOC616222	NC_007317:39649882..39659376	27.00	5.88	7.48	6.20	2.53
LOC616254	NC_007317:49763777..49777779	443.00	975.69	724.39	1,191.05	917.05
LOC616777	NC_007302:103112775..103216626	50.00	20.29	27.80	26.28	27.10
LOC616798	NC_007320:52603425..52626992	124.00	56.36	48.28	68.99	63.84
LOC616999	NC_007299:119302415..119330929	644.00	746.57	1,088.40	942.28	992.50
LOC617566	NC_007324:48404191..48567050	1,754.00	922.58	831.08	1,031.27	994.32
LOC617774	NC_007309:1304680..1598928	875.00	504.26	501.50	681.85	633.65
LOC618094	NC_007301:116751837..116753578	136.00	41.43	66.94	58.54	64.52
LOC618200	NC_007301:12868176..12873599	19,807.00	15,866.78	17,304.71	20,814.15	19,219.54
LOC618220	NC_007301:117643528..117663625	3,116.00	2,283.64	2,268.02	2,459.89	2,279.61
LOC618238	NC_007330:27770020..27773916	5,626.00	4,482.52	2,799.45	2,582.86	3,227.87
LOC618242	NC_007319:1775129..1777086	2,584.00	1,911.54	2,223.83	3,099.88	3,156.77
LOC618268	NC_007316:57405158..57423021	362.00	184.18	176.69	174.50	182.95
LOC618416	NC_007316:63295650..63304387	1,445.00	1,641.27	1,122.55	881.71	1,050.53
LOC618464	NC_007319:13257055..13268447	3,749.00	2,372.41	2,387.50	2,148.43	1,904.81
LOC618541	NC_007326:3071286..3075588	1,211.00	306.97	918.54	602.35	1,008.23
LOC618771	NC_007330:50281723..50318937	65.00	18.68	18.88	38.33	27.04
LOC618809	NC_007316:3885042..4049680	230.00	136.70	57.14	141.16	128.50
LOC618886	NC_007324:11122550..11162337	588.00	494.29	437.98	506.71	443.18
LOC768237	NC_007317:37414770..37450516	448.00	228.67	287.72	354.28	359.48
LOC777786	NC_007303:4355434..4356158	149.00	94.81	102.70	88.35	71.89
LOC781002	NC_007305:12221238..12224068	486.00	618.41	660.98	650.10	676.06
LOC781022	NC_007331:35883140..35884003	91,338.00	99,394.22	77,439.41	69,319.06	80,612.24
LOC781039	NC_007319:52038098..52043163	20,033.00	23,293.60	31,080.68	42,844.20	10,813.66
LOC781068	NC_007301:28067914..28068812	1,202.00	834.95	738.38	795.47	877.52
LOC781070	NC_007307:7003650..7011286	8,322.00	7,307.87	7,148.80	9,081.95	9,429.70

Continued on next page...

Table 1 – continued from previous page

Gene	Location	TCT1	TCT3	TCT4	TCT5	TCT6
LOC781081	NC_007324:27325486..27326188	211.00	1,230.34	2,418.67	684.64	2,061.36
LOC781156	NC_007299:58510412..58511318	931.00	1,505.13	1,970.86	1,308.89	1,257.99
LOC781225	NC_007301:57896817..57916331	560.00	1,045.61	1,199.61	1,289.79	1,096.25
LOC781251	NC_007310:67916934..67919901	2,613.00	5,560.78	6,233.89	3,638.71	5,273.07
LOC781300	NC_007318:3761127..3761980	1,800.00	1,377.44	1,298.08	1,253.03	1,152.86
LOC781339	NC_007310:68019915..68022882	2,481.00	5,363.74	6,003.50	3,491.37	5,076.47
LOC781382	NC_007307:50568933..50569592	613.00	492.74	459.76	336.69	343.74
LOC781512	NC_007304:57540898..57640804	612.00	994.52	1,061.52	862.35	1,226.02
LOC781542	NC_007314:40259851..40263375	1,568.00	997.12	972.83	1,071.70	1,042.02
LOC781555	NC_007306:111012852..112057857	416.00	276.20	233.08	320.27	303.86
LOC781576	NC_007301:70981013..70981474	1,929.00	1,371.45	1,552.40	1,652.72	1,248.52
LOC781608	NC_007319:46142867..46143527	1,129.00	774.53	860.84	665.96	715.61
LOC781612	NC_007301:28136456..28137365	1,202.00	834.95	738.38	795.47	877.52
LOC781675	NC_007301:58148065..58164989	475.00	879.19	974.02	1,060.84	904.62
LOC781710	NC_007301:58226537..58250335	868.00	1,966.93	2,134.81	2,068.34	1,340.79
LOC781728	NC_007302:21973491..21974357	86.00	56.19	25.61	26.28	31.97
LOC781730	NC_007303:67664847..67667245	367.00	860.10	657.54	502.75	459.70
LOC781813	NC_007301:12870212..12871977	19,807.00	15,866.78	17,303.37	20,812.19	19,218.89
LOC781851	NC_007303:21886929..21887777	86.00	52.33	16.40	26.65	15.83
LOC781914	NC_007316:38869243..38872772	137.00	216.93	203.76	195.07	190.94
LOC781963	NC_007307:29585752..29591300	29.00	74.66	65.36	114.02	91.67
LOC781979	NC_007300:75663019..75666072	208.00	337.73	346.02	314.06	315.26
LOC781982	NC_007320:58852175..58853072	2,123.00	1,694.75	1,772.45	1,450.30	1,466.88
LOC781989	NC_007307:53774277..53774921	724.00	492.74	543.90	379.62	513.19
LOC782019	NC_007309:103308973..103311719	197.00	93.90	72.92	70.38	73.66
LOC782021	NC_007320:44238163..44238732	4,454.00	3,660.67	4,023.08	3,150.53	2,339.58
LOC782039	NC_007303:49257260..49272307	379.00	219.95	253.89	166.18	141.14
LOC782052	NC_007312:64878425..64879287	232.00	325.83	377.64	359.12	342.90
LOC782090	NC_007330:20176235..20177709	334.00	508.53	560.25	468.66	458.85
LOC782107	NC_007301:29098195..29139548	40.00	15.45	15.40	17.56	21.29
LOC782171	NC_007302:44751844..44752406	1,052.00	807.01	765.98	778.72	653.96
LOC782233	NC_007301:30967015..30968172	776.00	591.27	651.35	641.32	597.49
LOC782372	NC_007306:33934427..33937197	715.00	550.89	548.77	572.39	507.88
LOC782437	NC_007309:22226526..22227646	3,839.00	3,077.33	3,439.10	3,391.67	3,334.15
LOC782444	NC_007327:23679232..23692964	1,134.00	1,437.66	1,270.08	758.31	1,020.13
LOC782449	NC_007301:35390223..35392188	297.00	383.42	390.95	528.79	462.63
LOC782470	NC_007306:33623166..33624150	561.00	405.55	342.98	335.67	309.61
LOC782479	NC_007309:45366162..45366703	2,039.00	1,571.94	1,596.78	1,645.25	1,463.25
LOC782484	NC_007303:116000342..116006256	1,327.00	778.94	908.57	848.44	831.02
LOC782525	NC_007306:22812452..22813886	5,592.00	3,858.48	4,569.40	5,311.28	5,011.95
LOC782568	NC_007306:73215357..73253475	525.00	805.02	733.17	1,068.56	1,063.88
LOC782688	NC_007330:29235963..29237204	1,533.00	1,799.34	2,045.86	2,033.19	1,767.02
LOC782691	NC_007306:73794155..73838963	733.00	1,060.68	1,151.39	1,429.14	1,435.99
LOC782705	NC_007313:37127008..37127980	92,049.00	100,599.59	85,648.39	68,276.87	74,197.42
LOC782706	NC_007315:30271928..30274021	1,442.00	1,689.06	1,230.16	1,122.68	1,097.75
LOC782779	NC_007300:80851629..80853962	363.00	791.79	794.01	601.27	685.32
LOC782814	NC_007309:22744823..22745402	1,174.00	898.36	1,029.05	975.86	885.39
LOC782831	NC_007315:38190600..38925118	1,065.00	574.46	434.85	884.26	691.81
LOC782849	NC_007314:25312060..25332425	847.00	645.99	582.33	496.28	380.39
LOC782861	NC_007299:139864962..140548441	2,438.00	1,781.99	1,777.43	2,157.40	1,963.49
LOC782921	NC_007299:22882140..22883060	9,935.00	13,090.21	13,469.76	6,055.62	8,321.28
LOC782938	NC_007304:12173212..12275617	1,418.00	1,649.24	1,935.71	1,763.74	2,024.82

Continued on next page...

Table 1 – continued from previous page

Gene	Location	TCT1	TCT3	TCT4	TCT5	TCT6
LOC782951	NC_007313:18562896..18564373	612.00	1,360.21	1,452.24	1,074.17	1,087.36
LOC783052	NC_007325:37655452..37666558	978.00	849.91	550.93	514.42	554.96
LOC783070	NC_007306:80082343..80083405	4,834.00	3,497.82	3,883.44	4,445.00	4,500.49
LOC783144	NC_007316:23522893..23523652	977.00	649.14	646.61	865.39	641.79
LOC783145	NC_007319:27737819..27742815	1,698.00	1,154.03	1,361.60	934.59	936.01
LOC783146	NC_007320:61049462..61050096	578.00	399.39	387.75	320.33	281.31
LOC783184	NC_007318:33462349..33481091	694.00	1,570.12	1,890.76	2,166.31	1,963.79
LOC783188	NC_007304:68832872..68835077	889.00	1,163.59	1,117.80	1,127.41	1,194.90
LOC783220	NC_007319:67683788..67684713	1,724.00	1,938.91	1,401.30	795.90	1,072.72
LOC783229	NC_007312:65052759..65053690	543.00	839.40	692.76	791.20	780.69
LOC783255	NC_007315:55941414..55942254	1,263.00	1,074.31	1,030.66	979.09	828.34
LOC783305	NC_007317:20076684..20080550	207.00	347.04	365.26	472.83	448.71
LOC783417	NC_007301:86323498..86324189	496.00	287.91	321.33	286.84	252.93
LOC783466	NC_007326:18056298..18060100	990.00	1,345.94	1,454.29	1,193.01	1,150.88
LOC783484	NC_007320:36141416..36786359	308.00	203.37	144.83	212.62	154.33
LOC783504	NC_007325:48324598..48325110	488.00	405.86	380.26	402.40	295.18
LOC783540	NC_007305:11164613..11196946	58.00	31.92	25.99	33.77	25.38
LOC783577	NC_007331:75060657..75063062	1,816.00	2,918.48	3,215.09	2,764.38	2,736.41
LOC783641	NC_007317:11837397..11857163	1,708.00	1,082.31	1,179.43	1,472.27	1,240.45
LOC783832	NC_007310:41960565..41961597	431.00	612.55	596.50	554.90	538.74
LOC783910	NC_007319:68475131..68478374	396.00	199.00	289.04	96.21	191.81
LOC783920	NC_007313:45243554..45250049	6,767.00	11,105.25	11,721.58	12,102.47	12,940.52
LOC783943	NC_007309:106834161..106884740	879.00	1,051.41	1,075.18	1,075.57	1,386.73
LOC783965	NC_007301:87333505..87810521	170.00	106.04	78.32	126.83	109.69
LOC784025	NC_007304:118755697..118756651	3,560.00	4,580.58	4,660.29	2,595.23	3,309.31
LOC784054	NC_007318:14646381..14647226	552.00	411.80	262.75	229.69	205.02
LOC784058	NC_007304:97563288..97564499	441.00	620.74	642.01	683.53	662.05
LOC784160	NC_007311:21947111..21947660	2,974.00	2,673.35	3,623.99	3,332.45	2,530.19
LOC784188	NC_007328:7860834..7881545	38.00	16.55	17.25	8.48	13.00
LOC784205	NC_007328:46213281..46217229	6,868.00	3,867.98	5,256.67	4,786.99	5,845.73
LOC784210	NC_007331:33376720..33377110	140.00	98.64	26.75	23.12	12.63
LOC784266	NC_007319:24871018..24894958	963.00	654.69	467.33	502.28	625.64
LOC784289	NC_007311:78191769..78192673	2,046.00	2,893.74	2,485.30	1,370.51	1,516.52
LOC784297	NC_007299:108597124..108598662	1,281.00	1,722.85	1,740.86	923.83	1,004.08
LOC784350	NC_007309:48925440..48926321	5,328.00	4,087.33	5,068.62	4,497.30	3,724.64
LOC784352	NC_007314:36820275..36828295	27.00	86.40	65.97	111.68	56.95
LOC784354	NC_007318:15385462..15386118	1,426.00	986.80	1,174.92	1,288.58	1,154.97
LOC784355	NC_007324:17272589..17286210	196.00	120.52	124.50	129.91	130.68
LOC784388	NC_007314:37587085..37587934	3,216.00	2,710.77	2,911.65	2,854.90	2,772.40
LOC784403	NC_007302:7662257..8520025	681.00	498.72	449.20	571.93	481.61
LOC784483	NC_007299:90728130..90728996	63.00	114.86	138.58	100.68	119.93
LOC784541	NC_007306:73377970..73439894	1,186.00	1,724.18	1,838.82	2,243.39	2,313.10
LOC784572	NC_007331:72896535..72948999	8,622.00	6,383.39	7,996.80	9,604.04	7,957.36
LOC784738	NC_007327:14530055..14530579	2,816.00	2,114.12	2,177.50	2,484.10	1,881.29
LOC784765	NC_007318:22370041..22375148	90.00	47.60	39.56	32.81	32.30
LOC784768	NC_007301:60861425..60894826	82.00	30.90	32.13	38.37	23.87
LOC784903	NC_007319:33539288..33545391	538.00	417.23	323.16	308.61	356.41
LOC785024	NC_007324:21221508..21224751	263.00	157.83	202.87	115.13	128.98
LOC785085	NC_007331:48742148..48804935	563.00	677.95	761.83	873.16	811.34
LOC785168	NC_007303:107391733..107411922	109.00	59.92	14.98	26.65	40.23
LOC785297	NC_007326:32678028..32678981	3,046.00	4,131.44	3,389.42	1,676.20	1,911.21
LOC785366	NC_007318:30447268..30506663	210.00	352.86	144.07	278.22	116.55

Continued on next page...

Table 1 – continued from previous page

Gene	Location	TCT1	TCT3	TCT4	TCT5	TCT6
LOC785375	NC_007307:90288135..90297765	8.00	25.15	40.57	31.52	46.53
LOC785386	NC_007303:119965806..119966378	2,756.00	2,133.56	2,237.90	2,289.39	1,922.57
LOC785387	NC_007305:4805004..4806708	2,524.00	3,851.83	3,835.94	2,737.00	3,123.41
LOC785477	NC_007320:7788268..7789324	436.00	301.38	266.67	297.80	289.03
LOC785516	NC_007301:17832645..17833168	4,916.00	3,494.27	3,883.35	2,946.41	3,349.40
LOC785605	NC_007304:101842538..101844192	167.00	276.81	265.60	234.20	348.94
LOC785666	NC_007311:43256057..43289206	604.00	943.38	966.76	930.80	1,139.38
LOC785760	NC_007324:29737955..29739774	1,454.00	1,120.58	1,049.76	1,057.12	1,052.27
LOC785761	NC_007300:63210945..63212562	2,490.00	2,278.29	2,230.29	2,243.95	2,227.10
LOC785769	NC_007310:34512576..34622443	1,108.00	554.02	708.76	744.01	920.50
LOC785787	NC_007307:20636791..20641512	210.00	396.08	368.11	406.44	353.47
LOC785842	NC_007331:62504921..62505825	700.00	854.09	826.67	446.99	547.58
LOC785953	NC_007326:28032076..28039165	43.00	151.80	152.17	76.76	110.10
LOC786055	NC_007325:37102992..37139863	2,048.00	1,825.55	1,616.87	1,540.33	1,402.26
LOC786090	NC_007303:109436151..109494872	1,768.00	619.67	1,110.39	794.45	1,243.90
LOC786101	NC_007303:110659411..110663744	13,916.00	12,875.69	15,422.12	15,442.31	18,628.80
LOC786143	NC_007306:17891960..17893097	1,301.00	1,442.65	1,127.78	1,017.26	1,082.56
LOC786156	NC_007317:15379879..15383105	71.00	19.85	15.64	40.67	8.23
LOC786337	NC_007320:52049019..52049553	3,573.00	2,878.22	3,160.99	2,789.37	2,338.17
LOC786352	NC_007303:79821766..79845353	2,541.00	1,425.15	1,821.41	1,647.80	2,082.18
LOC786414	NC_007303:9617666..9719969	1,140.00	473.28	895.73	472.60	1,003.82
LOC786460	NC_007301:57588225..57605009	264.00	497.88	554.96	545.05	523.93
LOC786565	NC_007305:52032952..52146639	2,657.00	3,791.63	3,529.27	3,238.96	3,807.93
LOC786695	NC_007324:26329850..26359972	1,605.00	1,931.96	2,061.59	1,062.05	2,027.84
LOC786726	NC_007313:55911074..55913832	2,831.00	3,493.67	3,894.52	3,390.09	3,497.59
LOC786732	NC_007313:53805405..53829143	968.00	1,327.10	1,474.70	1,303.26	1,170.39
LOC786739	NC_007303:110067444..110117549	269.00	85.71	206.82	103.07	209.73
LOC786921	NC_007302:124277858..124283137	939.00	202.14	161.68	458.52	560.14
LOC786978	NC_007303:10694989..10695862	1,459.00	1,040.61	1,096.13	1,323.15	953.04
LOC786982	NC_007302:124284487..124286374	242.00	28.10	24.14	89.90	129.96
LOC786987	NC_007324:28964769..28998461	2,912.00	2,685.95	2,727.72	3,945.28	2,192.61
LOC786990	NC_007309:30819685..30881049	254.00	326.07	327.39	334.33	358.30
LOC787171	NC_007313:5366127..5368783	108.00	197.04	227.21	170.80	192.40
LOC787404	NC_007325:15041415..15041609	3,116.00	2,583.15	2,859.46	2,813.86	2,346.54
LOC787498	NC_007326:28007610..28010204	485.00	301.07	289.45	362.58	321.30
LOC787514	NC_007319:20061926..20089403	184.00	105.05	73.83	86.84	120.66
LOC787596	NC_007311:67967092..68042741	888.00	1,316.07	1,347.52	1,172.27	1,252.37
LOC787677	NC_007309:99264061..99279554	2,075.00	1,350.86	1,645.89	1,806.54	1,411.04
LOC787679	NC_007311:47731474..47732605	294.00	368.21	405.97	363.02	388.28
LOC787697	NC_007320:52125918..52126453	3,573.00	2,878.22	3,160.99	2,789.37	2,338.17
LOC787851	NC_007305:7896294..7905499	120.00	54.73	58.04	77.52	43.07
LOC787914	NC_007306:26433334..26437003	5,431.00	4,011.42	3,508.74	2,933.77	2,911.49
LOC787939	NC_007324:31553547..31555029	751.00	908.23	614.94	537.49	510.80
LOC788054	NC_007303:115929632..115998272	716.00	455.84	492.79	431.93	464.98
LOC788082	NC_007307:87720033..87730027	13.00	55.80	69.87	76.46	88.19
LOC788092	NC_007317:55253845..55366806	641.00	910.99	786.29	775.42	943.00
LOC788116	NC_007307:87738684..87752848	19.00	66.01	47.33	71.76	70.14
LOC788125	NC_007327:14259256..14298532	200.00	301.79	308.57	293.98	269.47
LOC788159	NC_007302:114844791..115223995	201.00	116.29	98.77	142.46	119.53
LOC788405	NC_007316:60834045..60834676	815.00	653.24	618.79	577.40	529.26
LOC788499	NC_007312:15061963..15071034	897.00	1,571.03	1,521.40	731.11	1,316.33
LOC788541	NC_007303:81062339..81063467	4,301.00	3,586.78	3,761.21	3,829.20	3,522.62

Continued on next page...

Table 1 – continued from previous page

Gene	Location	TCT1	TCT3	TCT4	TCT5	TCT6
LOC788563	NC_007317:27002032..27010492	950.00	609.53	491.77	471.46	515.17
LOC788584	NC_007303:81149851..81189141	29.00	100.88	74.17	96.76	63.98
LOC788609	NC_007316:2329963..2332214	54,323.00	51,807.77	53,044.91	59,282.84	62,348.15
LOC788648	NC_007317:29281174..29281993	1,365.00	1,076.43	1,026.39	1,129.70	1,009.45
LOC788668	NC_007316:61583180..61583986	562.00	810.40	763.16	671.03	656.31
LOC788801	NC_007324:34668273..34669179	11,653.00	13,291.30	10,258.66	5,664.88	6,684.65
LOC788914	NC_007317:25722137..25757885	22.00	6.62	8.84	5.51	3.80
LOC788925	NC_007303:122097667..122123709	470.00	292.01	300.24	366.72	293.50
LOC789003	NC_007326:12127..37666	109.00	28.67	19.61	69.45	64.40
LOC789148	NC_007305:20259261..20261303	737.00	1,111.86	1,168.63	983.20	924.49
LOC789163	NC_007317:43992752..44012804	1,632.00	1,120.54	1,114.13	1,323.39	1,196.78
LOC789174	NC_007326:403297..431204	97.00	39.64	21.96	54.83	42.24
LOC789183	NC_007316:12933821..12960763	771.00	875.89	880.45	1,129.26	1,385.95
LOC789264	NC_007305:20968513..20969558	3,581.00	3,203.32	3,919.97	4,830.81	4,547.83
LOC789329	NC_007301:126989921..126991522	174.00	19.66	14.06	65.04	90.33
LOC789369	NC_007316:65509887..65510792	172.00	125.24	118.80	78.74	74.78
LOC789383	NC_007316:65443098..65443672	422.00	280.78	190.22	134.06	121.97
LOC789490	NC_007315:74065881..74069734	1,165.00	531.19	893.37	888.93	807.62
LOC789520	NC_007319:1864852..1907766	46.00	24.41	18.80	19.37	24.41
LOC789587	NC_007316:14574956..14575465	760.00	519.80	396.99	364.60	313.64
LOC789605	NC_007309:14721454..14722846	351.00	255.83	230.66	206.02	189.52
LOC789734	NC_007303:121657299..121703019	14.00	2.28	2.85	2.10	2.64
LOC789845	NC_007299:59197673..59217907	306.00	221.14	179.96	207.52	246.47
LOC789854	NC_007311:55794095..55860207	360.00	524.99	457.27	497.62	465.46
LOC789894	NC_007316:17662313..17679636	295.00	453.50	406.01	390.14	410.19
LOC790266	NC_007324:10585674..10587464	2,604.00	2,063.25	2,122.90	2,191.21	1,925.57
LOC790863	NC_007299:36428570..36433582	424.00	529.71	697.69	507.26	564.90
LOC790883	NC_007317:42538511..42546170	2,097.00	1,424.21	1,560.33	1,729.36	1,459.43
LPCAT1	NC_007318:74992303..75018279	1,161.00	854.57	747.95	648.05	862.19
LPIN2	NC_007325:38536172..38576903	2,698.00	3,118.72	3,259.18	2,912.62	3,571.14
LRCH4	NC_007326:38082422..38093009	1,092.00	762.36	802.45	833.35	792.18
LRP4	NC_007313:77139138..77176561	89.00	31.46	25.05	30.76	36.72
LRP8	NC_007301:99627630..99704481	276.00	537.20	488.68	394.81	614.26
LRRC16A	NC_007324:32376246..32685196	134.00	64.57	48.66	84.35	52.27
LRRC25	NC_007305:4697940..4701071	417.00	267.25	187.12	165.02	136.90
LRRC28	NC_007319:6016907..6207481	178.00	100.61	103.77	121.20	115.08
LRRC8D	NC_007301:56703803..56834619	989.00	664.31	554.29	878.07	725.24
LSM10	NC_007301:116698812..116701270	90.00	52.67	42.17	57.24	48.39
LSM14A	NC_007316:44339714..44392981	881.00	1,139.48	1,130.82	1,032.79	1,086.83
LSP1	NC_007330:51451992..51490105	6,128.00	5,297.30	6,836.31	5,385.25	5,180.97
LSS	NC_007299:148839164..148867577	243.00	463.71	597.61	610.25	671.59
LTA	NC_007324:27535254..27536817	30.00	154.96	105.54	256.12	138.07
LTA4H	NC_007303:64839218..64869766	295.00	180.51	215.37	220.87	210.39
LTBP1	NC_007309:16297282..16780983	429.00	277.27	214.29	354.12	328.98
LTV1	NC_007307:84110515..84127085	308.00	401.58	469.52	376.93	394.44
LY75	NC_007300:37616016..37922391	1,419.00	2,287.92	2,425.37	2,834.04	2,743.53
LY86	NC_007324:49471580..49513514	322.00	167.15	194.66	179.16	136.93
LY9	NC_007301:9691283..9718691	522.00	369.37	444.50	369.44	411.01
LYPLA1	NC_007312:21846406..21864400	384.00	602.82	673.58	648.12	642.48
LZTS2	NC_007327:22137727..22144867	245.00	165.55	124.95	122.19	111.21
MACF1	NC_007301:113619708..113978871	5,280.00	4,055.35	3,916.15	3,606.59	4,583.73
MAFB	NC_007311:70036219..70039597	1,645.00	1,380.57	1,281.81	810.88	998.55

Continued on next page...

Table 1 – continued from previous page

Gene	Location	TCT1	TCT3	TCT4	TCT5	TCT6
MAFF	NC_007303:117033769..117042682	287.00	591.60	561.97	370.93	480.15
MAFK	NC_007326:43316714..43320341	264.00	373.59	372.59	371.35	348.31
MAGI2	NC_007302:44634350..44866386	1,281.00	1,002.91	943.76	1,022.84	864.53
MAML2	NC_007313:12019744..12425874	493.00	812.15	735.19	896.09	724.91
MAML3	NC_007315:18775959..19236295	359.00	209.64	211.00	246.44	215.77
MAN1C1	NC_007300:131554717..131641583	133.00	41.72	87.51	46.25	67.56
MAN2A1	NC_007305:110809035..111017847	826.00	1,036.15	1,010.96	1,099.87	1,076.00
MAN2C1	NC_007319:33448970..33460554	458.00	251.52	288.34	329.85	378.03
MANSC1	NC_007303:104517998..104578571	107.00	53.85	42.08	32.25	25.72
MAP1LC3B	NC_007316:12238730..12252041	914.00	785.85	783.46	700.82	655.59
MAP2K1IP1	NC_007304:26484326..26495727	316.00	469.91	558.46	482.92	477.20
MAP2K3	NC_007317:36296884..36314726	1,040.00	1,274.21	1,169.23	1,232.40	1,199.95
MAP2K6	NC_007317:63037728..63145203	84.00	47.06	28.57	48.94	41.77
MAP3K7IP2	NC_007307:89786916..89827911	1,062.00	1,534.82	1,399.56	1,479.56	1,330.53
MAP3K8	NC_007311:35222366..35248681	564.00	909.33	856.94	749.70	700.98
MAP4	NC_007320:53050292..53142781	1,201.00	1,559.17	1,522.64	1,565.06	1,610.69
MAP7D1	NC_007301:116866326..116892445	821.00	564.59	538.22	457.25	489.73
MAPK14	NC_007324:10256729..10332294	745.00	507.20	526.46	607.68	575.00
MAPKAPK2	NC_007314:3471456..3532063	1,417.00	1,787.36	1,696.95	1,639.72	1,744.44
MAPKAPK3	NC_007320:50617715..50645798	1,696.00	2,388.99	2,112.69	1,513.66	1,915.86
10-Mar	NC_007317:48655314..48746023	52.00	25.00	17.68	26.88	22.15
7-Mar	NC_007300:37956998..38004349	701.00	882.44	965.01	910.86	920.26
MARCKS	NC_007307:38367154..38369954	615.00	389.01	290.73	150.91	168.75
MARS	NC_007303:60419515..60438282	836.00	1,009.52	995.57	1,260.74	1,055.93
MAT2A	NC_007309:51225940..51232519	1,472.00	1,720.55	1,753.78	2,018.43	2,039.68
MATR3	NC_007305:49904271..49931077	2,181.00	3,342.87	3,502.41	2,832.17	2,436.58
MBP	NC_007325:2090555..2123047	763.00	499.76	520.99	630.87	549.30
MCM7	NC_007326:38449418..38456845	1,313.00	944.52	1,082.48	1,186.43	1,025.54
MCOLN1	NC_007305:14841392..14850226	586.00	434.16	373.37	355.36	413.02
MCRS1	NC_007303:33295226..33304419	480.00	353.44	382.25	359.71	345.60
MDM2	NC_007303:48573170..48577713	230.00	449.77	315.93	335.87	319.88
MDM4	NC_007314:1255538..1292772	214.00	420.88	394.29	320.99	367.03
MDN1	NC_007307:63073546..63213059	579.00	862.89	830.87	995.32	1,059.70
MEA	NC_007324:17237096..17238735	345.00	276.92	204.14	174.85	181.25
MED13	NC_007317:10286239..10399981	1,174.00	1,782.28	1,722.22	1,519.83	1,692.33
MED21	NC_007303:88980743..88990520	274.00	169.14	179.00	189.32	163.57
METTL1	NC_007303:60127352..60130957	256.00	367.86	361.57	377.24	329.11
MFGE8	NC_007319:20237369..20252398	214.00	143.51	110.74	93.08	141.59
MFNG	NC_007303:81642453..81658180	1,689.00	812.32	1,034.08	1,127.51	1,075.71
MFSD1	NC_007299:110553883..110581472	515.00	395.14	414.77	342.02	375.50
MGAT4C	NC_007303:17930117..18856284	1,078.00	594.64	395.09	915.05	606.12
MGC128480	NC_007324:33026253..33108031	2,043.00	842.95	1,038.38	1,288.62	1,103.41
MGC133950	NC_007306:42046144..42116449	1,090.00	779.91	913.88	970.35	851.97
MGC134419	NC_007305:13113656..13118004	460.00	695.03	700.83	593.30	691.44
MGC139164	NC_007316:61071083..61080773	1,774.00	1,532.40	1,225.56	2,444.38	2,037.91
MGC139169	NC_007306:73874595..73910278	508.00	622.41	660.88	886.07	921.65
MGC139367	NC_007309:74896415..74912479	614.00	416.28	349.71	377.59	400.50
MGC142711	NC_007309:74684233..74692607	1,210.00	1,337.55	1,447.22	1,430.43	1,438.93
MGC142811	NC_007326:4368943..4373635	125.00	64.09	74.52	55.56	62.32
MGC143170	NC_007304:14383382..14391116	798.00	1,007.78	1,321.84	1,942.57	1,406.15
MGC148449	NC_007303:121579610..121593928	372.00	218.44	271.71	253.13	284.26
MGC148834	NC_007314:63856892..63891604	255.00	332.90	332.11	373.50	337.50

Continued on next page...

Table 1 – continued from previous page

Gene	Location	TCT1	TCT3	TCT4	TCT5	TCT6
MGC148992	NC_007310:10236891..10252167	241.00	485.59	470.83	306.40	486.16
MGC152020	NC_007311:39435994..39543701	746.00	1,256.04	1,066.67	901.43	1,102.78
MGC152033	NC_007312:486680..536443	615.00	724.90	731.11	803.36	1,080.26
MGC152536	NC_007305:8848083..8921317	793.00	514.43	575.22	569.50	549.15
MGC155012	NC_007304:14397324..14426201	294.00	429.30	480.19	600.02	583.77
MGC155285	NC_007310:29810056..29827594	169.00	76.67	116.45	60.11	67.82
MGC157163	NC_007304:74817382..74842314	302.00	394.49	462.60	412.11	454.57
MGC33894	NC_007317:36269116..36276601	501.00	348.51	389.74	366.69	401.25
MGST2	NC_007315:19241929..19280499	156.00	98.48	103.47	115.54	114.59
MIA3	NC_007314:23071864..23134087	1,029.00	1,246.00	1,283.70	1,234.41	1,185.46
MICAL1	NC_007307:42538415..42549621	785.00	562.69	929.28	419.86	949.29
MICAL2	NC_007313:39369033..39485716	1,242.00	1,392.50	1,604.29	853.19	1,464.98
MID1IP1	NC_007331:64950584..64952365	386.00	257.56	210.72	186.50	197.63
MIER1	NC_007301:84032052..84098811	435.00	672.73	643.99	552.21	522.64
MKL1	NC_007303:118891208..119002483	1,420.00	962.49	957.78	976.76	1,004.48
MLL	NC_007313:27575488..27625777	921.00	1,226.10	1,235.40	1,436.82	1,579.14
MLL5	NC_007302:48119790..48211724	1,864.00	2,597.42	2,359.41	2,224.80	2,730.50
MLSTD2	NC_007313:37779625..37856711	599.00	731.02	850.09	859.66	876.59
MMP1	NC_007313:4760544..4792670	46,918.00	22,487.83	11,351.82	18,777.41	15,728.37
MMP12	NC_007313:4707459..4717328	17,569.00	11,131.74	8,383.42	9,199.69	6,551.29
MMP19	NC_007303:61991908..61998019	2,573.00	1,605.67	1,802.87	1,027.24	1,780.77
MMP25	NC_007326:3010436..3046677	2,137.00	2,640.44	1,928.85	1,660.85	1,889.05
MMP3	NC_007313:4728091..4734670	2,062.00	891.63	714.46	965.70	1,000.34
MMP9	NC_007311:75519672..75526983	45,437.00	11,588.38	15,102.65	11,033.39	10,887.79
MON1B	NC_007316:3294735..3305934	509.00	636.86	605.26	662.52	646.15
MORC3	NC_007299:151865450..151907185	487.00	837.42	910.37	709.40	797.31
MORF4L2	NC_007331:34148532..34160671	655.00	927.68	1,112.76	749.86	806.88
MOSPD2	NC_007331:80557540..80574386	214.00	325.67	354.17	301.33	321.71
MOV10	NC_007301:33148608..33175737	2,444.00	3,495.68	3,861.26	3,350.97	3,940.43
MPEG1	NC_007313:83166107..83170210	624.00	970.28	1,333.88	913.90	965.21
MPP7	NC_007311:36244688..36467676	305.00	688.05	678.71	915.30	656.42
MPZL1	NC_007301:1478301..1560160	220.00	56.18	86.36	55.29	104.53
MRM1	NC_007317:12267070..12275953	151.00	106.61	99.31	108.90	92.40
MRPL41	NC_007309:109897288..109897930	362.00	245.48	267.12	261.01	226.00
MRPL51	NC_007303:110691888..110695208	314.00	227.54	256.74	228.59	205.12
MRPS25	NC_007320:58776707..58859071	2,302.00	1,834.42	1,932.61	1,614.34	1,608.59
MRPS34	NC_007326:1804750..1806003	308.00	170.35	234.54	247.08	222.28
MT1E	NC_007316:23606451..23608481	102.00	141.62	169.92	14.90	21.78
MT-2	NC_007316:23619146..23620657	6,918.00	11,330.93	13,584.91	4,495.07	5,379.72
MTDH	NC_007312:64683940..64738109	1,254.00	1,852.24	1,837.35	1,440.75	1,494.14
MTHFD2	NC_007309:10739330..10761919	446.00	641.05	771.60	833.62	814.59
MTMR10	NC_007319:27212395..27250934	630.00	465.31	500.76	456.05	541.94
MTMR2	NC_007313:12506881..12583201	271.00	338.60	353.34	339.98	332.91
MTSS1	NC_007312:14853262..15012900	1,276.00	2,370.02	2,265.85	1,032.99	2,007.37
MVD	NC_007316:12887956..12894619	333.00	532.90	596.99	766.79	863.22
MVP	NC_007326:28217538..28237111	3,144.00	3,595.09	3,514.14	3,984.00	3,733.09
MX1	NC_007299:144440108..144472307	467.00	1,548.85	1,205.80	1,987.54	734.45
MX2	NC_007299:144387774..144423561	768.00	1,349.13	1,654.25	1,144.41	1,023.10
MXD4	NC_007304:119341673..119349171	120.00	55.53	59.80	71.71	64.13
MYBL1	NC_007312:30719638..30753289	26.00	96.82	69.19	85.84	76.95
MYH9	NC_007303:80350411..80403133	11,647.00	8,524.38	8,741.22	9,826.48	10,540.82
MYLIP	NC_007324:41547993..41568655	163.00	80.35	109.34	68.34	90.91

Continued on next page...

Table 1 – continued from previous page

Gene	Location	TCT1	TCT3	TCT4	TCT5	TCT6
Mynn	NC_007299:99947559..99987323	190.00	293.14	331.04	255.94	286.17
MYO10	NC_007318:59682140..59966983	178.00	112.58	106.74	89.55	102.51
MYO18A	NC_007317:20409934..20497771	765.00	447.78	474.77	358.42	472.76
MYO1F	NC_007305:15580048..15622883	3,426.00	1,765.84	1,671.08	1,393.83	1,715.15
N4BP1	NC_007316:16288108..16342099	529.00	850.52	758.64	616.41	800.06
N4BP2L1	NC_007310:28390724..28413344	295.00	185.50	230.39	172.99	215.65
NAB1	NC_007300:5839632..5876300	338.00	519.03	617.38	427.46	570.47
NAGLU	NC_007317:43955756..43962736	290.00	195.58	178.21	178.52	198.73
NAGLU	NC_007327:32383148..32390262	289.00	210.70	199.62	185.45	223.13
NAIP	NC_007318:10733015..10769866	799.00	1,721.23	1,000.99	589.26	1,043.33
NAP1L4	NC_007330:50489836..50542346	1,531.00	1,719.73	1,955.93	2,021.86	1,891.87
NAPB	NC_007311:42194702..42233516	396.00	582.33	650.81	617.54	721.66
NARS	NC_007325:59152119..59168836	966.00	1,223.93	1,205.16	1,426.09	1,269.89
NAT13	NC_007299:59252863..59256761	516.00	1,042.28	1,170.20	849.28	913.93
NCBP2	NC_007299:72496388..72502210	283.00	354.86	457.11	363.54	362.26
NCF4	NC_007303:80921990..80940547	336.00	916.22	835.11	417.91	614.03
NCK1	NC_007299:134312113..134388579	442.00	663.42	573.55	530.32	537.60
NCL	NC_007300:123402544..123412496	3,687.00	4,319.12	4,277.52	4,794.51	3,988.06
NCOA1	NC_007309:76589073..76753904	563.00	715.73	666.69	730.98	746.64
NCOA3	NC_007311:77040016..77068958	1,052.00	1,494.35	1,640.97	1,820.00	1,853.88
NCOA6	NC_007311:64633995..64694889	711.00	874.39	823.63	811.70	866.47
NDFIP2	NC_007310:54998750..55070358	395.00	836.80	764.89	859.83	713.24
NDNL2	NC_007319:28011349..28012868	327.00	201.95	231.92	201.16	227.38
NDUFS2	NC_007301:9217378..9226906	1,079.00	951.52	944.56	951.57	933.65
NEDD4L	NC_007325:59644626..59827205	1,014.00	1,484.95	1,473.15	1,142.35	1,406.51
NEGR1	NC_007301:78293590..79358590	2,303.00	1,683.23	1,564.45	2,122.33	1,754.39
NEK6	NC_007309:98537211..98623433	1,536.00	2,042.19	1,789.49	1,889.39	2,069.72
NEURL	NC_007327:24585230..24657555	139.00	46.73	41.14	35.22	61.31
NFE2L3	NC_007302:72468862..72502759	274.00	379.31	408.96	468.89	407.25
NFKB1	NC_007304:23818259..23914105	2,284.00	3,729.44	4,147.56	4,035.82	4,963.49
NFKB2	NC_007327:23471865..23478382	3,320.00	4,801.71	4,480.72	5,336.94	5,255.36
NFKBIB	NC_007316:48227137..48237772	438.00	571.38	557.14	627.76	561.93
NFKBIZ	NC_007299:47295359..47306371	993.00	2,111.99	2,048.80	1,379.60	1,933.72
NFS1	NC_007311:65471266..65492826	1,042.00	1,290.98	1,335.82	1,742.50	1,424.23
NINJ1	NC_007306:88677284..88688556	772.00	598.06	430.76	462.46	474.83
NKAIN3	NC_007312:27559142..27876365	371.00	244.16	185.07	284.00	235.33
NKG7	NC_007316:57350250..57352981	436.00	644.23	599.25	537.68	551.77
NLGN3	NC_007331:49281616..49304128	37.00	70.46	71.32	66.28	121.85
NLRC3	NC_007326:3500104..3516394	347.00	183.00	251.79	199.57	249.29
NLRC5	NC_007316:24620666..24678470	5,338.00	5,035.15	5,623.29	5,923.67	6,990.74
NMD3	NC_007299:108008927..108045344	298.00	429.43	446.52	449.62	395.35
NMI	NC_007300:46979689..46999428	763.00	1,186.48	1,553.49	1,021.27	991.57
NMI	NC_007300:47179134..47187112	552.00	828.69	1,131.20	725.85	715.34
NOC2L	NC_007314:48771760..48783623	578.00	684.03	728.67	964.45	885.23
NOL5A	NC_007311:53076709..53081953	878.00	1,025.80	1,297.11	1,337.88	1,172.00
NOLA1	NC_007304:16999087..17006701	220.00	278.46	304.30	312.26	314.05
NOLC1	NC_007327:23024285..23033093	781.00	906.95	911.23	942.32	918.90
NOS2A	NC_007317:19241809..19282628	8,413.00	28,921.59	34,293.98	13,547.47	32,604.29
N-PAC	NC_007326:4506939..4538681	1,257.00	1,402.45	1,398.60	1,622.11	1,496.42
NPHP4	NC_007314:44532960..44638901	269.00	128.40	88.71	183.42	157.50
NPL	NC_007314:61645655..61684153	87.00	18.23	21.23	12.57	8.44
NPLOC4	NC_007317:52660811..52709404	981.00	1,190.39	1,176.03	1,256.53	1,319.56

Continued on next page...

Table 1 – continued from previous page

Gene	Location	TCT1	TCT3	TCT4	TCT5	TCT6
NQO2	NC_007324:51580497..51591400	1,846.00	1,088.30	1,020.05	935.22	1,009.66
NR4A1	NC_007303:30707515..30721239	172.00	327.66	416.49	289.59	662.18
NR4A3	NC_007306:67696788..67704316	27.00	71.52	78.92	68.16	158.76
NRAS	NC_007301:31065179..31074983	838.00	1,056.85	1,136.69	937.91	989.79
NRD1	NC_007301:101187698..101297397	1,389.00	1,560.34	1,541.02	1,575.32	1,702.77
NRSN2	NC_007311:60999930..61506814	4,638.00	6,024.88	6,261.42	5,721.86	5,934.02
NRXN1	NC_007309:33595999..34848487	2,791.00	1,496.52	1,279.80	2,543.39	2,132.66
NSDHL	NC_007331:22514048..22540046	354.00	519.81	589.20	652.75	570.61
NTNG2	NC_007309:106008471..106077254	211.00	104.25	117.56	127.57	146.61
NTRK3	NC_007319:18459648..18732139	82.00	43.65	28.56	39.98	38.36
NUB1	NC_007302:118184860..118224078	1,292.00	2,347.67	2,521.82	1,964.08	2,257.23
NUCB1	NC_007316:55308953..55324998	4,338.00	4,841.96	4,587.95	6,019.43	5,559.77
NUDC	NC_007300:130489820..130503258	1,522.00	1,835.47	1,926.81	2,107.44	1,917.09
NUDCD1	NC_007312:52887684..52980985	213.00	331.72	370.97	309.04	357.85
NUDT5	NC_007311:10685580..10705495	255.00	324.31	397.86	399.73	398.62
NUDT9	NC_007304:106391066..106428406	505.00	776.55	970.93	801.54	901.59
NUP188	NC_007309:103128391..103171594	989.00	1,111.30	1,232.18	1,464.88	1,568.38
NUP98	NC_007313:50801308..50864432	766.00	987.67	1,158.51	1,022.37	1,045.84
NXN	NC_007317:22048590..22074003	118.00	66.17	78.90	61.34	83.54
NXT2	NC_007331:35726054..35732631	111.00	167.53	215.58	218.10	190.95
OAS1Z	NC_007315:64606810..64614653	334.00	581.17	482.87	548.99	561.98
OAT	NC_007327:44907846..44927169	716.00	1,016.26	1,070.47	939.45	1,025.84
OBSL1	NC_007300:111880476..111902576	116.00	62.57	35.32	42.52	60.80
OGN	NC_007306:88188034..88203623	30.00	6.09	8.85	9.53	12.21
OPA1	NC_007299:75162500..75253901	506.00	674.57	812.21	641.76	715.43
ORAI3	NC_007326:28914290..28919336	381.00	273.24	280.82	247.08	227.82
OS9	NC_007303:60178607..60212603	2,459.00	2,094.12	1,934.81	1,714.41	1,907.40
OSGIN2	NC_007312:72473994..72538707	235.00	421.80	438.50	369.85	419.85
OSTM1	NC_007307:43895678..43938580	4,969.00	3,681.83	4,060.45	3,486.97	3,299.25
OTUD4	NC_007315:14062823..14105253	591.00	801.26	861.59	826.82	782.02
P2RX4	NC_007315:56660082..56676230	316.00	797.53	704.69	578.37	676.57
P2RX5	NC_007317:24471366..24484352	757.00	590.42	460.48	587.94	615.16
P2RY6	NC_007313:52316331..52321891	89.00	135.77	140.82	135.18	169.25
P4HA2	NC_007305:20831102..20902007	593.00	379.44	446.14	264.03	350.72
P4HB	NC_007317:52519184..52529128	3,923.00	3,433.68	3,412.46	2,903.87	2,701.78
P76	NC_007315:64247311..64269642	2,787.00	3,005.09	2,436.66	1,854.67	2,177.21
PADI4	NC_007300:139921125..139948930	107.00	23.26	19.27	29.84	21.02
PAF1	NC_007316:48588768..48592969	520.00	603.30	669.17	605.77	641.79
PAFAH2	NC_007300:131358171..131387260	368.00	583.21	474.48	452.07	571.22
PAI2	NC_007325:64893041..64910799	12,176.00	14,863.05	18,771.37	9,177.84	16,752.84
PANK3	NC_007318:2849270..2871083	140.00	208.54	204.53	196.88	223.97
PAPOLG	NC_007309:45167331..45197237	159.00	217.38	242.57	229.48	253.17
PARK7	NC_007314:42542500..42559257	826.00	941.64	1,053.20	1,127.91	1,118.66
PARP14	NC_007299:68098485..68145015	1,741.00	3,088.27	3,927.28	3,450.91	3,902.17
PARP9	NC_007299:68044516..68074962	959.00	1,247.99	1,624.42	1,385.75	1,461.45
PATL1	NC_007313:83710234..83738483	897.00	1,265.01	1,216.39	1,115.46	1,354.81
PATZ1	NC_007315:73578358..73595839	319.00	152.94	179.89	210.38	220.04
PBEF1	NC_007302:49240668..49281361	499.00	1,087.98	1,166.90	957.14	1,076.49
PBX1	NC_007301:4935131..5228688	94.00	42.84	37.49	57.89	44.52
PBXIP1	NC_007301:17015320..17026797	1,443.00	860.23	958.62	903.44	1,038.82
PCBP3	NC_007299:148279820..148509214	433.00	277.71	268.49	297.44	338.28
PCDH1	NC_007305:52377662..52392999	0.00	8.21	7.80	29.17	8.46

Continued on next page...

Table 1 – continued from previous page

Gene	Location	TCT1	TCT3	TCT4	TCT5	TCT6
PCDH9	NC_007310:39889208..41029241	2,016.00	1,084.95	958.42	1,617.77	1,319.03
PCDHGC3	NC_007305:51998005..52031018	145.00	79.35	94.43	59.87	100.76
PCGF5	NC_007327:13261506..13313723	374.00	470.51	495.99	515.37	561.75
PCMTD1	NC_007312:20767384..20815604	1,207.00	1,397.60	1,475.55	1,418.58	1,529.99
PCNP	NC_007299:47107115..47121948	585.00	761.14	1,028.73	823.15	740.24
PCP4L1	NC_007301:9159012..9184831	6.00	31.60	30.12	23.42	45.81
PCTK2	NC_007303:65171208..65280648	395.00	508.17	479.96	563.06	470.25
PCYT1B	NC_007331:72600684..72717164	159.00	332.71	55.11	89.40	87.67
PDE1B	NC_007303:28360276..28385481	171.00	342.83	304.52	371.63	316.58
PDE4B	NC_007301:84657115..85109825	2,401.00	3,056.79	2,780.80	2,660.22	2,780.31
PDGFC	NC_007315:44450843..44697387	142.00	92.51	59.51	96.84	91.43
PDIA3	NC_007319:55841289..55863497	5,258.00	6,654.15	6,661.04	5,711.23	5,828.80
PDIA6	NC_007309:89603586..89627269	1,487.00	2,026.66	2,040.25	1,650.38	1,737.88
PDLIM4	NC_007305:20832094..20846508	13.00	0.91	1.73	0.00	3.08
PDPN	NC_007314:51382128..51415168	1,643.00	1,486.17	1,143.43	871.26	1,006.16
PDS5B	NC_007310:28109125..28241057	516.00	862.35	905.64	761.61	693.42
PDXK	NC_007299:147712363..147738298	5,860.00	6,226.25	6,249.38	5,279.36	6,238.68
PELI1	NC_007309:64300160..64356728	466.00	886.53	950.92	1,016.91	940.46
PF4	NC_007304:91905161..91906129	809.00	484.00	665.76	556.45	439.48
PFKP	NC_007311:45272721..45325512	952.00	1,187.06	1,255.70	1,541.00	1,641.44
PFN1	NC_007317:26821480..26824443	7,571.00	6,189.45	6,050.20	7,902.40	7,253.48
PGCP	NC_007312:65173815..65734542	551.00	347.72	295.94	428.51	410.89
PGM1	NC_007301:87921428..87987137	288.00	168.53	186.77	184.72	190.99
PGM2	NC_007304:59361249..59400228	212.00	270.18	268.24	302.28	310.28
PHB	NC_007317:38399485..38410103	512.00	597.77	683.58	947.05	843.63
PHC3	NC_007299:99539655..99616189	330.00	456.00	434.97	434.25	606.25
PHCA	NC_007313:55827655..55977535	3,121.00	3,836.42	4,150.24	3,660.45	3,788.99
PHIP	NC_007307:18429964..18544086	439.00	598.84	590.47	551.99	573.60
PI4KA	NC_007315:75564315..75623909	2,508.00	2,135.94	1,801.63	2,318.17	2,252.79
PICALM	NC_007330:9887076..9965461	4,375.00	6,682.35	6,544.05	5,677.07	5,977.79
PIK3CA	NC_007299:90197931..90226702	296.00	420.85	537.94	423.49	507.00
PIK3CD	NC_007314:40846086..40862364	4,029.00	3,733.25	3,694.92	5,207.60	4,601.91
PIK3IP1	NC_007315:73546901..73557591	566.00	181.29	239.41	345.96	288.91
PIK3R3	NC_007301:106866104..106963631	70.00	28.79	31.46	34.47	31.62
PIK3R5	NC_007317:28906260..28933343	3,356.00	4,327.03	3,786.56	3,088.59	3,857.43
PIM2	NC_007331:55484773..55493445	1,340.00	1,212.64	1,524.47	1,521.29	1,552.09
PIN1	NC_007305:12884703..12896957	592.00	480.68	438.34	383.00	343.03
PIP4K2A	NC_007311:23079645..23265362	1,408.00	1,675.32	1,658.97	2,059.02	1,935.04
PITRM1	NC_007311:45246036..45272024	678.00	920.98	954.15	1,182.88	972.29
PLA2G7	NC_007324:20651152..20689045	527.00	203.02	254.70	229.03	233.52
PLAA	NC_007306:17738295..17778376	630.00	790.57	877.00	763.68	821.08
PLAGL2	NC_007311:62543984..62549019	92.00	163.95	201.63	198.23	258.59
PLAT	NC_007328:39469375..39493390	55.00	146.86	100.87	101.79	158.85
PLCE1	NC_007327:15852044..16220772	1,237.00	552.89	427.42	868.29	660.13
PLD2	NC_007317:26917471..26930177	78.00	39.70	39.45	39.29	47.47
PLD3	NC_007316:49363210..49375631	1,317.00	991.31	684.21	611.45	712.21
PLEK	NC_007309:68697839..68725085	1,669.00	2,575.29	3,116.92	1,812.41	2,197.02
PLEKHA7	NC_007313:34328109..34427201	165.00	27.32	35.42	101.18	75.84
PLEX2	NC_007302:99084496..99526644	101.00	61.66	44.63	62.24	60.46
PLOD1	NC_007314:38460817..38489937	458.00	336.86	346.52	269.22	316.40
PLSCR1	NC_007299:124225839..124253664	492.00	900.85	1,121.12	599.49	701.37
PLXNA2	NC_007314:73453501..73659392	151.00	68.17	72.79	69.52	65.39

Continued on next page...

Table 1 – continued from previous page

Gene	Location	TCT1	TCT3	TCT4	TCT5	TCT6
PMPCB	NC_007302:46175895..46190073	266.00	391.02	436.03	401.11	355.82
PMVK	NC_007301:17042863..17052654	33.00	60.39	68.28	67.64	56.14
PNPT1	NC_007309:39811573..39852248	262.00	365.26	502.99	453.84	480.60
POLQ	NC_007299:67004181..67109588	1,576.00	876.85	670.75	1,411.88	1,124.83
POLR1E	NC_007306:64388315..64404134	191.00	253.38	251.52	321.74	273.69
POLR2B	NC_007304:75005845..75049785	1,059.00	1,383.21	1,558.41	1,865.41	1,818.28
POLR2C	NC_007316:25226318..25234627	733.00	885.71	1,004.51	859.72	993.91
POT1	NC_007302:92220273..92317109	185.00	527.60	453.59	400.42	434.35
PPARD	NC_007324:9571090..9657851	1,636.00	1,500.09	1,238.72	1,035.58	1,129.54
PPBP	NC_007304:91896307..91897903	5,710.00	5,038.06	4,579.38	6,828.03	4,363.68
PPIL2	NC_007315:75441730..75459210	565.00	435.69	409.83	452.81	380.95
PPM1B	NC_007309:27707242..27769443	716.00	902.05	1,046.91	1,016.18	1,082.06
PPP1CB	NC_007309:72987435..73003944	1,484.00	1,626.65	1,902.59	1,815.34	1,704.98
PPP1R13L	NC_007316:52788202..52802991	30.00	81.04	69.17	72.35	108.90
PPP1R15A	NC_007316:55296087..55299492	1,163.00	2,007.18	1,554.88	928.52	1,465.09
PPP1R15B	NC_007314:1154631..1160501	1,148.00	1,681.94	1,483.12	1,014.75	1,366.16
PPP1R16B	NC_007311:68203623..68311207	3,255.00	4,666.71	5,503.49	5,267.48	5,611.78
PPP1R1B	NC_007317:41265259..41273804	169.00	59.56	67.34	67.55	80.38
PPP1R9A	NC_007302:12432899..12788476	1,269.00	519.02	536.26	649.39	374.58
PPP2CA	NC_007305:45026813..45051476	1,788.00	2,246.52	2,381.43	2,331.75	2,207.38
PPP2R2A	NC_007306:77467748..77546291	541.00	687.08	685.23	775.40	708.30
PPP2R3A	NC_007299:135546917..135548959	2.00	16.29	19.25	13.07	13.23
PPP2R5D	NC_007324:17213928..17237452	540.00	408.92	408.91	432.21	396.02
PPRC1	NC_007327:23008804..23022703	432.00	573.48	652.95	719.50	804.84
PPT1	NC_007301:113117613..113133983	494.00	421.34	367.52	286.19	280.68
PRCC	NC_007301:15464528..15492242	729.00	977.50	439.81	425.38	552.32
PRDM1	NC_007307:45918508..45941333	1,716.00	2,545.46	2,373.17	1,403.10	1,910.38
PRDX1	NC_007301:107467592..107481411	3,270.00	5,825.66	5,387.56	3,779.60	3,876.56
PRDX5	NC_007330:44397597..44400705	3,698.00	4,733.53	4,178.05	3,421.75	3,282.74
PREP	NC_007307:46745777..46878477	564.00	679.78	661.84	650.58	704.85
PRICKLE1	NC_007303:41321001..41434189	413.00	221.47	184.71	617.75	237.44
PRKAG1	NC_007303:33837818..33852649	393.00	495.28	492.08	477.51	501.91
PRKX	NC_007331:87847212..87891144	1,039.00	1,398.96	1,580.40	1,271.60	1,578.10
PRMT1	NC_007316:56031246..56041327	2,208.00	2,454.13	2,683.45	3,690.68	3,985.79
PRMT8	NC_007303:113196324..113258759	18.00	4.55	2.85	6.31	3.30
PRNP	NC_007311:47214467..47234369	549.00	1,272.17	1,404.23	835.36	1,257.94
PRPF6	NC_007311:54513734..54548436	1,305.00	1,549.90	1,568.95	1,837.13	1,841.15
PSCDBP	NC_007300:40316361..40345477	2,032.00	3,089.33	3,222.59	3,171.97	2,940.94
PSD4	NC_007309:48564099..48609148	1,478.00	970.82	1,112.39	1,301.39	1,312.35
PSIP1	NC_007306:30535429..30575069	703.00	549.36	608.52	606.10	532.30
PSMA1	NC_007313:36997375..37010261	1,139.00	1,561.39	1,957.63	1,817.27	1,663.77
PSMA2	NC_007302:80899503..80910158	506.00	725.84	870.60	771.80	815.19
PSMA5	NC_007301:36695854..36717083	997.00	1,163.59	1,269.90	1,182.47	1,200.78
PSMA6	NC_007319:46349767..46373408	1,314.00	1,662.98	1,668.74	1,452.48	1,494.69
PSMA7	NC_007311:55776176..55781727	1,871.00	2,590.92	2,790.45	2,716.54	2,799.92
PSMB2	NC_007301:117351945..117389047	1,121.00	1,397.43	1,526.30	1,406.86	1,496.29
PSMC3	NC_007313:77601093..77607209	1,625.00	2,034.94	2,239.26	2,151.59	2,015.05
PSMC4	NC_007316:49126469..49137208	692.00	964.30	1,056.39	975.34	1,038.92
PSMD1	NC_007300:123040871..123122125	1,662.00	2,522.16	2,877.37	2,406.58	2,557.37
PSMD10	NC_007331:34454128..34461430	73.00	106.47	136.16	141.03	132.25
PSMD14	NC_007300:36107441..36217361	797.00	1,059.73	1,191.41	1,067.52	1,044.12
PSMD2	NC_007299:84753924..84763529	2,612.00	3,668.55	4,354.55	3,620.00	4,300.83

Continued on next page...

Table 1 – continued from previous page

Gene	Location	TCT1	TCT3	TCT4	TCT5	TCT6
PSMD4	NC_007301:21068886..21077916	1,255.00	1,415.69	1,413.83	1,632.56	1,660.83
PSMD8	NC_007316:47651063..47657055	1,362.00	1,695.30	1,684.20	1,539.97	1,785.25
PTGES3	NC_007303:61326976..61348216	1,401.00	1,843.07	2,222.21	1,880.59	1,731.30
PTGS2	NC_007314:65512065..65519688	2,422.00	3,243.41	6,539.84	2,181.85	4,534.41
PTK2B	NC_007306:78540637..78678950	2,133.00	2,574.86	2,466.52	2,750.55	2,655.04
PTMA	NC_007300:123579670..123584569	3,744.00	3,131.85	3,358.27	3,433.82	3,214.92
PTP4A2	NC_007300:125784352..125794040	1,008.00	1,147.97	1,242.79	1,190.61	1,155.96
PTPN1	NC_007311:79549390..79610066	2,367.00	4,187.41	3,724.80	3,214.16	3,224.01
PTPN11	NC_007315:64954584..65000560	732.00	949.72	912.31	842.18	997.79
PTPN18	NC_007300:1299899..1319129	501.00	369.02	382.15	400.60	344.54
PTPN2	NC_007325:44695571..44763522	3,460.00	4,438.94	4,433.66	3,870.71	4,325.00
PTPN23	NC_007320:53504856..53525705	824.00	1,050.34	1,022.25	982.12	1,168.74
PTPN6	NC_007303:10486602..10496094	1,619.00	1,313.66	1,339.32	1,157.66	1,150.24
PTPRCAP	NC_007330:47287882..47290040	1,180.00	558.08	946.94	730.00	789.67
PTPRE	NC_007327:48210443..48246781	988.00	1,695.89	1,457.51	1,408.81	1,657.45
PTPRM	NC_007325:41891023..42556214	373.00	222.03	176.66	290.38	210.23
PTX3	NC_007299:112140952..112146838	676.00	1,986.85	895.93	797.01	799.79
PUM1	NC_007300:126500923..126627202	1,080.00	1,346.92	1,342.35	1,596.43	1,707.67
PVR	NC_007316:52197135..52223657	3,082.00	4,277.13	3,448.86	2,426.64	3,500.81
PVRIG	NC_007326:38289664..38293447	727.00	403.11	498.88	411.56	430.71
QSER1	NC_007313:63136671..63232558	235.00	417.25	425.05	428.21	459.85
R3HDM1	NC_007300:64600800..64694489	318.00	387.47	427.11	427.46	440.62
RAB11FIP4	NC_007317:18336299..18442191	297.00	118.38	146.92	153.02	187.97
RAB12	NC_007325:42642288..42662443	363.00	467.13	440.90	505.58	550.00
RAB18	NC_007311:36940212..36968319	238.00	386.13	455.47	357.31	416.13
RAB33B	NC_007315:19536087..19555805	130.00	217.10	215.74	254.46	199.31
RAB35	NC_007315:65745974..65764750	1,236.00	1,115.35	1,014.43	1,108.66	1,048.99
RAB37	NC_007317:58325794..58395177	384.00	208.81	236.70	215.05	230.37
RAB3IL1	NC_007330:42058179..42076697	335.00	149.47	105.22	173.35	205.97
RAB6IP1	NC_007313:42392746..42487185	2,147.00	4,111.28	4,104.45	3,807.77	4,343.05
RAB7B	NC_007314:2744532..2772868	465.00	294.06	224.44	211.53	184.92
RAB8A	NC_007305:7352056..7372234	893.00	1,147.43	1,143.50	1,031.56	1,119.07
RABAC1	NC_007316:51006456..51009339	639.00	513.26	468.42	529.87	537.97
RABGGTB	NC_007301:73856039..73867367	402.00	492.96	530.86	530.74	523.28
RAC1	NC_007326:40118446..40136233	3,063.00	3,352.21	2,811.31	2,731.05	2,835.65
RAC2	NC_007303:81296826..81313652	2,284.00	2,105.50	2,628.00	3,315.93	2,800.42
RAD21	NC_007312:44689347..44718908	1,256.00	1,626.60	1,632.28	1,519.44	1,553.90
RAD23A	NC_007305:10875536..10881571	799.00	1,100.00	1,030.89	983.20	972.17
RAD54B	NC_007312:68168222..68286312	231.00	177.65	159.23	182.42	144.18
RALGDS	NC_007309:106974578..106994434	809.00	995.22	1,023.10	1,019.85	1,334.52
RALGPS1	NC_007309:101124553..101422683	422.00	283.92	235.87	341.66	272.48
RALYL	NC_007312:76464451..76648147	1,046.00	490.84	481.85	774.03	576.74
RANBP2	NC_007309:46334446..46396240	2,095.00	2,902.10	2,837.89	2,736.21	2,948.67
RANBP3	NC_007305:16980288..17031103	953.00	1,098.18	1,123.58	1,198.88	1,299.82
RAP1A	NC_007301:34160841..34179483	1,300.00	1,818.76	1,782.69	1,495.32	1,546.62
RAP1B	NC_007303:48808645..48852515	1,244.00	1,599.60	1,787.90	1,877.79	1,498.48
RAP1GDS1	NC_007304:27933905..28084377	752.00	952.25	1,109.01	1,108.35	1,116.62
RAPGEF1	NC_007309:105460231..105570369	1,631.00	1,892.09	1,873.57	1,978.11	1,936.70
RARG	NC_007303:29720423..29741044	615.00	275.32	375.12	390.56	448.49
RARS	NC_007305:81383859..81413130	486.00	622.97	699.96	673.12	570.69
RASA1	NC_007305:88162496..88272314	398.00	615.67	634.12	522.69	484.55
RASA4	NC_007326:36688532..36710959	70.00	201.55	181.20	111.84	153.73

Continued on next page...

Table 1 – continued from previous page

Gene	Location	TCT1	TCT3	TCT4	TCT5	TCT6
RASD2	NC_007303:79214651..79225952	81.00	18.20	22.82	18.93	22.42
RASGEF1B	NC_007304:99642884..99681165	1,065.00	2,593.20	2,971.72	2,334.71	2,684.04
RASGRP2	NC_007330:44753739..44766045	881.00	454.10	599.82	748.29	772.97
RB1	NC_007310:17508776..17628174	530.00	702.41	777.46	631.86	691.90
RBBP6	NC_007326:23383756..23418614	876.00	1,207.64	1,175.04	1,035.11	990.23
RBM14	NC_007330:46566510..46575712	693.00	917.14	1,020.68	1,035.76	967.01
RBM26	NC_007310:54850938..54926655	382.00	636.46	596.50	584.95	603.51
RBM39	NC_007311:65491308..65518204	2,363.00	3,425.00	3,398.95	3,595.13	3,397.46
RBM47	NC_007304:61766604..62052774	614.00	1,306.13	1,063.27	1,030.29	1,213.76
RBM5	NC_007320:51124697..51148827	1,598.00	2,101.49	1,869.77	2,293.03	2,578.09
RBM7	NC_007313:23219537..23226835	184.00	250.85	292.00	248.51	245.89
RBMS1	NC_007300:37102031..37320739	914.00	1,281.94	1,281.33	1,132.42	1,348.87
RBP4	NC_007327:15393347..15398956	452.00	213.87	153.90	163.16	260.20
RBPJ	NC_007304:47098412..47209172	554.00	761.63	817.02	685.34	1,009.11
RBPMS	NC_007328:28580528..28759524	249.00	151.69	120.78	125.12	127.80
RCHY1	NC_007304:93553552..93569055	287.00	359.68	415.99	456.59	514.93
RCSD1	NC_007301:1573807..1645214	1,169.00	653.07	939.21	790.91	838.80
RELA	NC_007330:45692931..45701575	1,453.00	1,643.37	1,807.55	1,632.47	1,693.06
RELB	NC_007316:52480272..52506805	1,116.00	1,497.20	1,374.43	1,658.43	1,684.34
RETN	NC_007305:14964453..14965840	3,619.00	6,299.93	4,198.05	7,435.80	5,437.70
REXO1	NC_007305:43800126..43819290	682.00	802.65	838.57	834.30	881.42
RFNG	NC_007317:52300659..52304235	461.00	269.84	327.17	356.35	313.91
RFX2	NC_007305:16919256..16963002	80.00	153.23	135.14	138.92	183.05
RGL1	NC_007314:62449947..62629779	755.00	625.38	369.26	481.49	293.90
RGL2	NC_007324:7759149..7766260	653.00	515.81	460.73	491.31	496.02
RGS1	NC_007314:11360134..11363749	2,642.00	6,274.40	6,545.91	4,465.03	4,895.11
RGS14	NC_007305:38167260..38181039	416.00	178.77	301.47	242.54	289.96
RHOG	NC_007313:50765437..50778902	1,910.00	2,124.35	2,325.66	2,228.49	2,654.53
RHOH	NC_007304:61579571..61627674	850.00	1,558.08	1,748.38	1,555.87	1,774.90
RING1	NC_007324:7689796..7692784	543.00	454.83	414.59	423.59	377.84
RIOK2	NC_007305:98021562..98043307	314.00	439.64	435.74	393.74	414.56
RIOK3	NC_007325:34236704..34258951	730.00	1,031.35	973.86	1,003.05	964.10
RIP5	NC_007311:66290634..66298058	793.00	926.35	995.56	1,030.33	997.76
RIPK2	NC_007312:72556691..72595294	406.00	534.62	624.40	482.16	523.69
RND2	NC_007317:44461487..44465279	196.00	108.82	98.63	88.91	140.50
RNF13	NC_007299:120038781..120199685	1,792.00	3,445.69	2,549.22	2,120.51	2,312.52
RNF139	NC_007312:15052229..15061031	245.00	309.83	325.96	330.50	305.55
RNF182	NC_007324:43372581..43374949	41.00	11.48	10.11	19.70	15.91
RNF19A	NC_007312:62125690..62198945	1,131.00	2,115.76	1,875.70	1,708.30	2,122.42
RNF19B	NC_007300:124905344..124929130	1,901.00	4,701.78	4,334.52	3,890.36	4,938.35
RNF213	NC_007317:53632185..53773500	3,282.00	4,971.12	4,685.08	5,838.06	4,909.28
RNPEPL1	NC_007301:125910666..125923697	1,273.00	756.30	849.50	950.27	1,006.56
ROBO2	NC_007299:24298584..24914408	2,075.00	1,823.99	1,464.67	1,767.73	1,690.56
ROCK1	NC_007325:36192006..36262780	1,355.00	2,213.10	1,982.91	1,683.30	1,709.47
RPL10	NC_007299:54125718..54126513	6,761.00	5,417.11	8,612.87	8,263.74	8,186.46
RPL10	NC_007331:23716778..23719136	9,844.00	8,113.49	9,322.72	10,453.31	8,536.14
RPL10A	NC_007324:9693949..9696424	4,206.00	2,675.19	3,215.00	3,430.57	2,887.50
RPL11	NC_007300:133635315..133639206	3,272.00	2,679.40	2,915.11	2,839.26	2,683.48
RPL13	NC_007316:13525607..13527996	9,158.00	5,778.43	6,346.59	8,197.81	6,915.96
RPL13A	NC_007316:55891981..55895506	13,178.00	9,938.51	12,130.03	10,477.62	10,289.72
RPL14	NC_007320:13583450..13586864	9,783.00	8,930.33	8,947.33	7,796.43	7,322.49
RPL17	NC_007309:32331287..32332048	1,287.00	865.08	849.73	721.44	570.71

Continued on next page...

Table 1 – continued from previous page

Gene	Location	TCT1	TCT3	TCT4	TCT5	TCT6
RPL17	NC_007325:50818284..50822849	3,630.00	2,818.70	3,037.61	3,198.57	2,916.37
RPL19	NC_007317:40954204..40957602	6,926.00	4,849.80	5,585.64	5,946.96	4,827.64
RPL21	NC_007310:32614624..32621079	3,734.00	3,317.51	3,353.62	3,028.10	2,644.92
RPL23	NC_007317:40681636..40685998	7,636.00	6,208.56	6,716.77	6,845.07	5,554.82
RPL23A	NC_007304:95706482..95707055	7,590.00	6,924.33	8,403.30	10,502.57	9,624.24
RPL27A	NC_007303:114155440..114155998	4,499.00	3,458.60	3,651.39	3,986.27	3,816.12
RPL28	NC_007316:62490995..62493749	3,517.00	2,764.38	3,205.25	3,914.83	3,234.37
RPL29	NC_007320:49935242..49937417	4,801.00	3,317.63	4,070.59	4,048.88	4,185.28
Rpl3	NC_007303:117900269..117906529	8,906.00	6,753.37	7,258.56	9,485.70	8,498.87
RPL34	NC_007304:18084060..18087942	1,925.00	1,301.99	2,189.87	2,195.83	1,630.60
RPL35	NC_007309:99277869..99282485	3,502.00	2,168.62	2,572.26	2,717.15	2,069.72
RPL36	NC_007303:31211973..31212398	1,580.00	1,026.96	1,055.48	1,138.03	955.68
RPL38	NC_007317:58931225..58935089	1,468.00	1,299.95	1,322.27	1,740.39	1,299.94
RPL5	NC_007301:53996664..54005273	4,504.00	3,132.63	3,482.36	3,797.16	3,793.97
RPL6	NC_007315:65070597..65074530	4,806.00	3,692.20	3,869.03	4,145.45	3,528.52
RPL7A	NC_007304:62152798..62153930	1,494.00	1,205.85	1,656.91	2,695.08	2,712.33
RPLP0	NC_007315:65830793..65834816	20,937.00	14,280.16	17,501.60	25,690.18	21,696.54
RPN1	NC_007320:61085929..61099909	2,417.00	2,688.74	2,642.97	2,888.63	2,879.75
RPN2	NC_007311:66729568..66783875	2,332.00	2,647.37	3,207.22	2,821.77	2,786.40
RPS10	NC_007324:8758804..8765761	8,931.00	6,360.48	6,010.34	7,274.27	5,847.15
RPS11	NC_007316:55901691..55904123	12,249.00	9,318.83	11,139.06	11,749.46	11,739.56
RPS12	NC_007307:73072166..73075507	12,855.00	10,735.08	14,136.83	14,365.06	11,628.27
RPS14	NC_007305:61226043..61246507	7,489.00	6,872.73	7,815.68	10,451.41	10,603.91
RPS17	NC_007319:22579780..22583228	7,508.00	5,366.97	6,571.89	5,429.48	6,463.50
RPS18	NC_007324:7740088..7744748	10,469.00	6,882.03	8,365.82	11,175.22	9,111.92
RPS19	NC_007316:51086619..51094150	2,298.00	1,895.04	1,747.36	1,749.93	1,534.06
RPS2	NC_007326:2002371..2004548	17,443.00	13,133.07	13,957.71	15,957.18	14,689.29
RPS20	NC_007309:98056775..98072398	4,064.00	2,983.43	3,301.45	3,430.53	3,111.73
RPS20	NC_007312:23167503..23168751	7,301.00	5,693.94	6,810.88	6,539.19	5,712.83
RPS3	NC_007313:54160921..54165866	14,438.00	10,833.70	13,328.46	16,956.09	15,948.72
RPS3A	NC_007315:7220419..7225105	8,997.00	6,254.89	6,962.36	8,257.51	6,685.24
RPS5	NC_007316:65984011..65989603	7,625.00	5,188.23	6,630.80	6,511.71	6,823.75
RPS6	NC_007300:115539331..115540147	6,746.00	5,342.75	5,273.85	4,584.89	4,366.37
RPS6KA2	NC_007307:105161107..105316237	405.00	192.54	216.36	234.75	299.30
RPS6KC1	NC_007314:68226177..68430916	270.00	420.88	344.24	335.78	360.00
RPS7	NC_007306:116399822..116404987	8,484.00	5,387.11	6,796.93	6,473.65	5,542.10
RPS9	NC_007316:63610157..63617470	8,378.00	7,262.53	6,649.60	6,543.63	6,255.29
RRN3	NC_007326:14768449..14795270	292.00	403.95	372.59	396.21	382.24
RRP12	NC_007327:20666494..20695696	1,013.00	1,217.46	1,340.94	1,294.52	1,420.77
RSAD2	NC_007309:92860125..92877429	757.00	1,308.72	1,189.03	905.47	1,130.69
RSF1	NC_007330:19136934..19280164	418.00	589.76	622.30	621.98	510.54
RSPRY1	NC_007316:24976669..25013895	972.00	1,607.71	1,250.37	1,117.92	1,172.50
RSRC1	NC_007299:110825172..111295551	2,534.00	2,353.70	2,354.83	2,297.28	2,001.54
RUFY3	NC_007304:88965086..89063839	270.00	438.42	372.01	356.74	343.29
RUNX3	NC_007300:132433807..132463009	1,970.00	2,273.48	2,372.39	2,597.56	3,174.38
RUSC1	NC_007301:16639667..16645613	113.00	70.22	55.56	69.60	67.10
S100A11	NC_007301:20241003..20242476	2,006.00	1,783.65	1,692.98	1,274.83	1,143.35
S100A12	NC_007301:18499228..18500355	7,242.00	13,315.60	5,813.31	8,960.86	6,190.36
S100A13	NC_007301:18155117..18161772	102.00	168.53	249.70	219.84	186.47
S100A4	NC_007301:18222399..18224692	1,036.00	1,176.23	1,756.58	1,196.78	1,220.78
S100A8	NC_007301:18481731..18483650	3,335.00	5,708.39	2,684.41	4,140.58	2,060.87
S100A9	NC_007301:18511305..18514006	6,740.00	9,363.47	3,939.58	9,567.70	4,932.16

Continued on next page...

Table 1 – continued from previous page

Gene	Location	TCT1	TCT3	TCT4	TCT5	TCT6
SAA3	NC_007330:27684332..27688065	35,985.00	29,786.25	29,252.63	27,097.84	26,075.02
SAFB	NC_007305:17186130..17235189	3,412.00	5,050.34	4,122.68	3,750.90	3,934.84
SAFB2	NC_007305:17239629..17286896	1,591.00	2,336.82	1,959.55	1,929.56	1,964.34
SAMD9	NC_007302:10705119..10725602	1,445.00	2,506.11	2,375.50	2,315.40	2,371.90
SAMHD1	NC_007311:66500426..66567426	9,044.00	10,952.30	10,398.49	16,832.75	11,501.24
SAP30BP	NC_007317:57603078..57637078	668.00	532.33	557.07	568.64	525.29
SAPS3	NC_007330:47749966..47874834	1,222.00	1,348.49	1,583.63	1,850.25	1,709.76
SASH1	NC_007307:88830702..89019930	472.00	198.83	61.60	135.48	90.28
SASH3	NC_007331:6447533..6461899	1,637.00	1,092.08	1,421.55	1,371.78	1,342.57
SAT1	NC_007331:73468721..73471768	3,258.00	6,803.00	7,576.18	4,564.65	6,001.82
SATB1	NC_007299:158985352..159078764	1,797.00	2,513.13	2,829.26	2,796.08	2,916.29
SBDS	NC_007326:30146136..30151619	511.00	862.72	793.82	612.59	672.38
SBNO2	NC_007305:42616534..42655194	3,944.00	5,234.58	4,493.45	5,440.23	5,572.30
SC4MOL	NC_007315:493451..510407	443.00	922.86	1,132.78	1,073.93	913.06
SC5DL	NC_007313:30429014..30440961	451.00	778.21	951.17	1,024.80	1,045.05
SCML4	NC_007307:44206910..44237860	150.00	66.80	94.66	98.59	100.69
SCRN1	NC_007302:69060572..69127115	103.00	47.61	49.75	45.64	66.72
SCYE1	NC_007304:20330334..20357791	293.00	375.43	460.84	393.05	432.88
SDAD1	NC_007304:94049795..94086408	307.00	400.29	430.06	463.86	406.47
SDC1	NC_007309:80982714..81006666	75.00	11.09	5.95	5.13	9.30
SDCBP	NC_007312:24561204..24579915	2,592.00	3,080.60	3,205.36	2,241.97	3,014.42
SEC16A	NC_007309:107792880..107818149	1,869.00	2,159.75	2,222.55	2,255.98	2,674.04
SEC23B	NC_007311:38530806..38560892	932.00	1,379.68	1,557.25	1,474.93	1,468.79
SEC24A	NC_007305:45391066..45438129	347.00	488.89	557.03	499.66	455.32
SEC24B	NC_007304:17229536..17310380	547.00	890.09	1,006.99	1,081.12	1,117.56
SEC24D	NC_007304:7308054..7477330	782.00	1,178.50	1,228.61	1,228.17	1,222.25
SEC61A2	NC_007311:10708311..10731008	275.00	377.17	387.06	357.31	420.90
SEC63	NC_007307:43995190..44062844	522.00	716.72	729.45	618.39	663.88
SEH1L	NC_007325:44792224..44812071	1,568.00	2,012.56	2,296.55	2,275.85	2,336.63
SELK	NC_007320:48067560..48074786	468.00	782.45	707.30	566.74	631.37
SELL	NC_007314:34766862..34792450	2,834.00	1,158.02	1,290.53	1,567.97	1,305.69
SELPLG	NC_007315:67589061..67605272	1,183.00	667.72	640.44	787.42	710.09
SELT	NC_007299:119192254..119222577	691.00	812.57	1,183.67	1,005.30	1,055.36
SEMA3E	NC_007302:38292890..38562208	83.00	35.90	21.95	52.56	50.04
SEMA4A	NC_007301:15943413..15962532	668.00	1,446.58	1,188.23	1,430.93	1,633.08
SEMA4B	NC_007319:21335558..21363556	136.00	65.10	89.84	94.33	89.97
SEMA4D	NC_007306:93387904..93520532	1,935.00	2,279.63	2,393.49	2,800.39	2,583.92
SENP6	NC_007307:15034150..15166880	582.00	779.59	755.00	786.06	684.02
SEPHS1	NC_007311:27592332..27617605	310.00	452.43	392.46	459.28	470.23
SEPN1	NC_007300:131518119..131535897	437.00	325.70	286.61	229.02	246.95
11-Sep	NC_007304:95019677..95120079	778.00	1,524.10	1,954.17	1,753.76	1,999.35
5-Sep	NC_007315:76321856..76325985	189.00	142.50	108.21	56.77	126.78
SEPX1	NC_007326:1985436..1990036	449.00	334.80	328.67	288.02	255.52
SERBP1	NC_007301:83613674..83628102	2,906.00	3,485.14	3,877.33	4,037.82	3,888.17
SERF2	NC_007319:55883355..55885782	2,224.00	1,641.53	1,755.80	1,675.51	1,740.20
SERINC3	NC_007311:73678562..73695041	875.00	1,032.97	1,274.61	1,212.25	1,339.09
SERINC4	NC_007319:55884571..55891096	1,790.00	1,405.55	1,390.15	1,431.24	1,470.98
SERP1	NC_007299:119397558..119400228	490.00	641.14	833.38	643.30	653.40
SETD2	NC_007320:53788535..53847895	1,176.00	1,610.63	1,472.83	1,312.31	1,447.24
SETD5	NC_007320:17601166..17680325	1,225.00	1,684.95	1,500.42	1,429.88	1,682.25
SF3A1	NC_007315:72843420..72862727	1,971.00	1,768.89	1,574.39	1,707.74	1,617.67
SF3B1	NC_007300:90148985..90186732	5,035.00	5,756.69	6,232.43	6,596.09	7,060.36

Continued on next page...

Table 1 – continued from previous page

Gene	Location	TCT1	TCT3	TCT4	TCT5	TCT6
SF3B3	NC_007316:929690..968567	2,031.00	2,367.36	2,603.00	2,922.47	2,914.92
SFMBT2	NC_007311:15192125..15349630	379.00	481.09	500.48	637.12	638.91
SFRS10	NC_007299:83160607..83180305	1,910.00	2,538.84	3,094.86	2,490.96	2,658.24
SFRS12	NC_007318:14188886..14233454	441.00	598.43	562.83	557.81	548.35
SFRS15	NC_007299:2848762..2910241	809.00	1,156.28	1,248.14	980.70	1,081.82
SFRS2IP	NC_007303:37467843..37517435	848.00	1,073.99	994.15	1,009.71	1,007.78
SFXN3	NC_007327:22165941..22175399	218.00	49.11	115.81	65.41	124.75
SGK1	NC_007307:74523692..74529249	1,658.00	3,798.14	5,932.54	1,831.01	4,201.32
SGSH	NC_007317:53941223..53948364	488.00	302.19	253.71	298.45	275.30
SGTB	NC_007318:14628294..14672485	566.00	425.40	276.93	246.09	223.97
SH3PXD2B	NC_007318:4128765..4224699	863.00	760.12	496.39	447.75	542.03
SIDT2	NC_007313:26303801..26317900	435.00	550.54	666.94	750.38	783.19
SIGLEC10	NC_007316:57372513..57380066	167.00	95.78	108.27	79.45	114.71
SIGLEC8	NC_007316:57399989..57404683	61.00	16.37	10.53	14.19	12.34
SIPA1	NC_007330:45680859..45690125	2,420.00	1,445.16	1,916.36	1,928.65	1,964.23
SIRPA	NC_007311:53757203..53799071	3,807.00	4,722.26	4,057.86	3,277.79	4,292.58
SKIL	NC_007299:99373071..99394240	461.00	741.42	795.85	657.90	655.05
SKIP	NC_007317:22713030..22731935	870.00	572.77	665.22	716.14	755.03
SLA	NC_007312:7732247..7750788	672.00	532.10	515.19	487.17	549.84
SLAMF7	NC_007301:9779556..9795548	357.00	1,216.96	1,145.39	669.28	959.46
SLAMF8	NC_007301:10823211..10833454	28.00	224.01	232.96	111.87	210.99
SLC12A7	NC_007318:75293424..75332510	620.00	1,009.47	1,041.30	741.70	1,675.23
SLC15A3	NC_007330:39015643..39030560	336.00	943.94	881.29	597.59	792.85
SLC16A3	NC_007317:52030853..52042271	974.00	619.09	703.31	431.48	666.43
SLC23A2	NC_007311:47384043..47533580	521.00	805.41	762.42	603.68	743.14
SLC25A20	NC_007320:51974892..52009954	408.00	309.55	310.35	330.89	289.03
SLC25A21	NC_007319:47725795..48259795	378.00	198.26	162.28	307.99	272.71
SLC25A46	NC_007305:111775135..111801178	476.00	565.51	639.32	571.81	587.61
SLC28A3	NC_007306:81707157..81780079	328.00	978.51	708.83	561.40	673.82
SLC29A1	NC_007324:18366257..18376490	97.00	152.09	161.79	181.01	252.84
SLC2A1	NC_007301:110215435..110249235	1,108.00	1,316.67	1,417.18	799.37	1,371.76
SLC2A13	NC_007303:44269752..44789228	344.00	260.15	236.77	276.27	242.71
SLC2A3	NC_007303:108852130..108865009	16,195.00	25,055.11	28,504.36	12,584.96	22,907.25
SLC30A9	NC_007304:63264058..63324708	527.00	618.26	637.61	675.36	696.94
SLC33A1	NC_007299:114149353..114170122	278.00	359.14	464.81	358.93	360.61
SLC35A2	NC_007331:55495250..55502599	347.00	475.19	516.26	507.87	581.76
SLC35A5	NC_007299:58077026..58103194	413.00	618.00	709.24	539.54	545.87
SLC38A10	NC_007317:52925411..52964047	1,131.00	827.91	671.34	956.01	985.40
SLC39A11	NC_007317:59921273..60213511	268.00	175.73	154.40	199.89	160.75
SLC39A8	NC_007304:24030933..24113079	1,874.00	2,635.47	4,397.33	1,684.77	2,910.38
SLC3A2	NC_007330:42846790..42887614	1,014.00	1,199.83	1,452.33	1,201.27	1,266.02
SLC43A2	NC_007317:22781005..22820455	1,994.00	1,480.82	1,020.95	1,215.86	1,230.96
SLC44A1	NC_007306:99817919..100014491	2,529.00	2,126.69	2,230.49	1,616.76	2,070.30
SLC44A2	NC_007305:13575568..13590254	3,267.00	2,969.82	3,601.17	4,163.06	3,830.24
SLC46A1	NC_007317:19768862..19776335	130.00	82.35	75.50	75.13	77.84
SLC46A2	NC_007306:107373181..107416931	105.00	219.90	653.51	164.90	395.81
SLC4A8	NC_007303:31157600..31230515	1,606.00	1,063.37	1,075.45	1,171.69	978.76
SLC5A6	NC_007309:74510383..74521704	59.00	99.08	183.79	211.89	194.53
SLC6A6	NC_007320:59482131..59526797	371.00	455.75	461.31	298.51	490.36
SLC7A1	NC_007310:30854805..30880859	340.00	434.47	495.96	447.87	585.98
SLC7A5	NC_007316:12563370..12592282	524.00	954.48	1,199.24	1,078.90	1,311.17
SLC8A1	NC_007309:23633272..24078448	308.00	226.99	159.98	248.55	193.81

Continued on next page...

Table 1 – continued from previous page

Gene	Location	TCT1	TCT3	TCT4	TCT5	TCT6
SLC9A2	NC_007309:7541324..7635886	73.00	38.45	35.72	45.46	42.91
SLC9A3R1	NC_007317:58307570..58324730	748.00	546.30	648.21	581.74	598.71
SLCO3A1	NC_007319:14175630..14609052	609.00	780.45	697.17	918.34	916.48
SLCO4A1	NC_007311:55386967..55409966	649.00	1,099.26	1,318.71	755.41	1,260.32
SLIC1	NC_007316:18116817..18125517	162.00	88.41	95.49	106.40	98.74
SLMO2	NC_007311:58063925..58072590	319.00	572.48	575.19	494.36	424.09
SMAP	NC_007313:34441469..34467994	801.00	937.99	1,106.67	994.85	1,021.09
SMC6	NC_007309:83025707..83088173	426.00	881.35	784.25	922.33	730.91
SMCHD1	NC_007325:38325272..38451555	940.00	1,149.92	1,306.97	1,524.11	1,371.12
SMPDL3A	NC_007307:29634231..29651620	51.00	107.67	93.15	169.02	132.64
SNAPIN	NC_007301:18120789..18122474	241.00	176.26	151.96	159.35	156.79
SND1	NC_007302:95098992..95535712	3,627.00	3,338.09	3,300.98	4,002.15	4,001.58
SNRPB	NC_007311:53224367..53233386	1,151.00	1,314.28	1,679.67	1,436.58	1,297.72
SNX10	NC_007302:72320292..72350997	657.00	2,869.81	2,938.10	1,206.11	1,787.44
SNX11	NC_007317:39582311..39590833	383.00	303.66	309.48	201.95	208.85
SNX17	NC_007309:74377260..74383003	1,010.00	678.76	876.52	902.54	881.10
SNX4	NC_007299:71078833..71131742	304.00	463.71	550.45	481.13	470.61
SOAT1	NC_007314:58253102..58313014	1,658.00	2,851.86	3,339.30	1,894.14	2,540.37
SOD2	NC_007307:99818144..99828056	10,741.00	17,241.36	12,570.49	11,391.84	11,772.71
SON	NC_007299:1042883..1073741	3,622.00	4,865.97	5,766.29	4,709.84	5,589.42
SORCS1	NC_007327:27568170..28146386	242.00	167.93	140.95	188.32	146.85
SORCS3	NC_007327:25510215..26142251	280.00	168.72	115.05	221.38	187.49
SOX13	NC_007314:897405..911101	2,797.00	1,200.82	1,637.80	1,136.04	1,930.06
SOX4	NC_007324:36890805..36891751	113.00	25.11	36.66	25.24	43.18
SP1	NC_007303:29556631..29591567	858.00	1,061.09	1,069.74	1,143.64	1,188.50
SP140	NC_007300:122390759..122435787	1,578.00	2,298.35	2,736.88	2,125.34	2,113.00
SP3	NC_007300:23539746..23597463	593.00	698.73	786.78	763.15	700.33
SPECC1L	NC_007315:74844112..74907397	708.00	467.77	430.79	498.90	484.57
SPEN	NC_007314:48900285..48991988	967.00	1,292.77	1,187.41	1,154.53	1,272.65
SPHK1	NC_007317:57056577..57059538	368.00	280.87	273.43	193.68	223.41
SPHKAP	NC_007300:120732876..120921577	81.00	46.53	39.34	51.47	51.79
SPIB	NC_007316:56526617..56532794	271.00	200.55	341.35	183.72	131.41
SPN	NC_007326:28335424..28339134	1,883.00	1,439.55	1,496.65	1,699.59	1,603.06
SPTA1	NC_007301:11969130..12067445	441.00	180.47	145.94	267.97	219.38
SPTAN1	NC_007309:102762819..102810747	6,148.00	3,785.66	4,224.84	5,767.16	5,846.56
SPTY2D1	NC_007330:27332863..27349518	214.00	350.93	392.09	332.77	338.77
SQLE	NC_007312:14605537..14627772	523.00	1,144.18	1,480.55	1,392.82	1,366.39
SR140	NC_007299:128014902..128064841	656.00	980.57	1,041.24	846.97	881.67
SRBD1	NC_007309:28885678..29128090	940.00	765.27	636.93	825.56	630.79
SRC	NC_007311:66916242..66935164	144.00	63.61	76.51	94.63	85.93
SRD5A2	NC_007309:14966442..15012791	57.00	26.62	17.86	30.06	31.47
SREBF2	NC_007303:119985622..120043210	2,380.00	2,939.81	3,306.22	4,015.72	3,930.22
SRPK1	NC_007324:10071107..10142827	517.00	734.62	627.58	650.77	600.57
SRPK2	NC_007302:48211707..48354987	1,732.00	1,419.69	1,531.97	1,401.13	1,358.65
SRPR	NC_007330:31200613..31205736	1,527.00	1,930.13	2,392.08	2,356.37	2,329.25
SRRM1	NC_007300:132666100..132695465	1,297.00	1,684.65	1,677.93	1,609.11	1,617.59
SRXN1	NC_007311:61247139..61254004	910.00	1,990.68	1,822.80	1,027.06	1,397.18
SS18	NC_007325:31765744..31843124	816.00	1,041.69	1,086.89	1,092.97	1,131.86
SSBP3	NC_007301:98388723..98553864	472.00	191.01	199.49	183.42	268.42
SSBP4	NC_007305:4667316..4683260	407.00	249.01	249.49	275.54	323.03
SSPO	NC_007302:116930838..116983338	121.00	23.41	44.63	23.51	82.70
SSR2	NC_007301:16090974..16097552	1,686.00	1,050.53	1,226.39	1,207.83	1,185.94

Continued on next page...

Table 1 – continued from previous page

Gene	Location	TCT1	TCT3	TCT4	TCT5	TCT6
SSR3	NC_007299:113265277..113276591	727.00	831.42	1,124.00	852.35	857.68
SSR4	NC_007331:23315193..23318947	2,517.00	1,760.64	2,754.75	2,877.67	2,159.11
ST13	NC_007303:119245129..119269984	1,807.00	2,360.34	2,313.50	2,047.47	2,108.56
ST3GAL6	NC_007299:44021590..44085223	530.00	651.42	692.88	660.98	646.78
ST6GALNAC2	NC_007317:56841794..56860613	587.00	420.57	500.61	478.35	434.79
ST6GALNAC5	NC_007301:72308057..72520937	144.00	98.31	51.55	93.01	70.33
STAM2	NC_007300:46138987..46186571	187.00	292.01	281.80	243.94	262.72
STAMBP	NC_007309:11201334..11225342	314.00	394.83	430.82	447.24	379.76
STARD13	NC_007310:27588005..27814506	136.00	200.34	246.31	219.17	223.27
STARD8	NC_007331:50801011..50834218	241.00	482.24	508.97	379.94	475.51
STAT1	NC_007300:83383381..83415778	8,158.00	9,840.77	10,338.95	8,890.02	9,749.84
STAT3	NC_007317:43752931..43784155	2,370.00	2,648.42	2,581.28	2,784.62	2,942.91
STAT4	NC_007300:83519326..83588177	213.00	301.63	273.77	352.11	315.26
STAT5B	NC_007317:43655234..43691654	1,879.00	1,500.67	1,462.39	2,349.70	2,291.04
STAT6	NC_007303:60839803..60849623	2,034.00	2,236.71	2,381.25	2,401.58	2,687.64
STCH	NC_007299:22551444..22561557	176.00	291.43	341.63	298.98	319.25
STIP1	NC_007300:120481515..120483627	792.00	1,266.70	1,543.86	1,175.69	1,235.53
STIP1	NC_007330:44280794..44293344	946.00	1,454.10	1,880.39	1,552.33	1,516.52
STK24-LIKE	NC_007310:76824126..76860443	2,346.00	1,246.54	1,072.35	1,570.13	1,269.50
STK25	NC_007301:126567483..126576992	492.00	292.13	307.94	351.23	360.68
STK38	NC_007324:10699037..10740337	3,194.00	1,995.81	1,732.32	1,874.75	1,719.32
STMN1	NC_007300:131440356..131445861	522.00	226.22	272.16	183.51	237.20
STON1-GTF2A1L	NC_007309:32131791..32182142	293.00	203.33	184.53	236.82	185.95
STRA8	NC_007302:102561055..102583818	53.00	18.73	16.83	17.29	5.56
STT3A	NC_007330:30553480..30574692	1,642.00	1,841.59	2,382.19	2,372.92	2,262.45
STT3B	NC_007320:6018764..6121718	845.00	967.03	1,052.13	999.72	1,082.45
STX11	NC_007307:84355469..84386427	452.00	906.12	785.05	789.41	862.49
STX16	NC_007311:58444483..58469795	532.00	631.61	697.61	750.52	833.05
STX8	NC_007317:29210782..29431892	1,727.00	1,376.41	1,296.42	1,450.90	1,278.43
STXBP1	NC_007309:101896168..101964559	547.00	883.57	978.46	782.30	770.24
STXBP5L	NC_007299:66666617..66990464	1,188.00	1,015.71	909.40	938.43	828.74
STXBP6	NC_007319:35152665..35393526	227.00	153.87	161.58	151.81	161.12
SUCLA2	NC_007310:16970720..17015390	397.00	568.86	552.09	477.93	511.30
SULT1A1	NC_007326:27936857..27939268	729.00	1,080.30	834.61	501.47	598.98
SUPT3H	NC_007324:18888191..19284659	342.00	214.50	201.61	283.21	209.66
SV2B	NC_007319:15234630..15490908	133.00	88.77	78.00	89.34	95.55
SVIL	NC_007311:34485831..34606490	1,930.00	1,492.56	1,170.19	1,715.58	1,439.35
SWAP70	NC_007313:41961141..42014558	357.00	510.80	581.41	463.02	507.75
SYMPK	NC_007316:53136454..53169678	1,058.00	1,371.96	1,329.32	1,459.11	1,690.15
SYT1	NC_007303:8968052..10990081	42,487.00	34,800.61	40,137.45	39,186.71	45,844.84
TACC3	NC_007304:118773830..118783048	615.00	809.70	758.98	786.10	798.80
TADA3L	NC_007320:17349029..17359259	512.00	361.82	396.94	397.78	361.28
TAF1	NC_007331:49011983..49086146	670.00	773.46	872.05	979.52	939.13
TAF11	NC_007324:9153922..9161567	198.00	142.76	139.67	126.83	114.20
TAF2	NC_007312:80208724..80296937	805.00	1,006.94	1,068.73	1,017.97	1,024.23
TAF8	NC_007324:16418772..16438534	304.00	237.46	209.82	205.64	227.84
TAGLN2	NC_007301:10734007..10741816	2,125.00	2,321.56	2,330.28	1,571.42	2,419.62
TANK	NC_007300:36278083..36383812	539.00	717.18	785.98	881.77	685.32
TAPBP	NC_007324:7767150..7776826	4,551.00	3,906.25	4,181.33	4,258.05	3,861.93
TARDBP	NC_007314:39343803..39355279	778.00	932.92	972.07	1,001.43	985.07
TARS	NC_007318:42787904..42811345	2,740.00	6,118.01	8,419.99	4,122.76	5,910.33
TAX1BP1	NC_007302:71057613..71140358	894.00	1,493.83	1,310.29	1,087.16	1,027.15

Continued on next page...

Table 1 – continued from previous page

Gene	Location	TCT1	TCT3	TCT4	TCT5	TCT6
TAX1BP3	NC_007317:24459963..24465096	742.00	562.48	536.66	397.02	489.85
TBC1D17	NC_007316:56177643..56186878	615.00	487.88	508.27	452.56	527.08
TBC1D9	NC_007315:18101881..18217559	2,277.00	3,948.85	3,762.85	2,748.94	3,529.74
TBCC	NC_007324:17018708..17021357	500.00	357.98	385.52	333.70	275.57
TBKBP1	NC_007317:39991449..40009033	405.00	544.83	508.09	499.72	722.75
TBL1XR1	NC_007299:92125015..92245028	1,118.00	1,345.71	1,666.76	1,348.85	1,350.63
TBL3	NC_007326:2011625..2017683	378.00	303.60	494.96	579.69	467.41
TBX21	NC_007317:39957707..39969532	448.00	1,038.19	1,151.54	1,166.23	1,179.06
TBX5	NC_007315:63478895..63526860	21.00	5.97	2.71	6.01	3.05
TBXA2R	NC_007305:18931628..18941102	371.00	201.58	266.82	252.52	241.50
TBXAS1	NC_007302:106978167..107151560	230.00	155.31	144.86	120.33	134.82
TCEA1	NC_007312:21814089..21833010	643.00	825.09	864.49	840.56	827.00
TCERG1	NC_007305:57694173..57746429	767.00	1,004.23	995.37	991.64	1,020.63
TCF19	NC_007324:27911488..27915229	54.00	21.52	20.22	27.71	28.41
TCF7	NC_007305:44951991..44983913	3,578.00	1,598.92	1,836.54	2,716.28	2,656.55
TCF7L2	NC_007327:34080102..34121635	186.00	320.80	332.95	135.13	240.95
TCIRG1	NC_007330:47576297..47587755	4,743.00	6,246.12	6,342.61	6,099.56	7,080.79
TCP11L2	NC_007303:74816546..74857243	668.00	314.00	361.57	437.54	380.56
TCTEX1D2	NC_007299:72061141..72089668	195.00	509.14	510.04	285.91	357.30
TCTN1	NC_007315:57242058..57269528	202.00	137.27	125.11	103.52	82.29
TDRD7	NC_007306:65419381..65522660	467.00	618.60	565.74	609.77	584.74
TEX10	NC_007306:68154030..68201657	238.00	321.86	306.10	385.50	358.46
TFRC	NC_007299:71808501..71836056	2,537.00	5,609.97	5,127.30	3,340.24	5,125.43
TG	NC_007312:7658632..7894999	2,418.00	1,935.59	2,113.29	2,140.39	2,070.87
TGFA	NC_007309:14501299..14616110	161.00	75.42	69.94	91.65	67.23
TGFB1	NC_007316:50175911..50190726	1,954.00	2,289.60	2,225.56	1,747.81	2,270.23
TGM3	NC_007311:53351411..53393993	60,080.00	38,981.23	32,583.49	30,847.00	42,081.58
TGS1	NC_007312:22927623..22953424	384.00	488.32	544.37	467.14	475.88
THAP4	NC_007301:126628403..126672717	373.00	224.71	208.86	259.52	309.71
THAP7	NC_007315:75694621..75695192	72.00	42.52	43.96	45.41	32.30
THOC2	NC_007331:2706551..2763931	689.00	904.20	966.88	937.13	922.79
THRA	NC_007317:41631766..41656523	377.00	211.76	200.65	162.67	211.38
TIAL1	NC_007327:40201143..40220585	859.00	991.71	1,049.89	1,076.73	1,082.15
TIE1	NC_007301:109614377..109633909	135.00	38.62	89.70	55.94	85.17
TIMP2	NC_007317:55010104..55061862	672.00	791.88	975.38	272.26	806.93
TK1	NC_007317:55555562..55568949	378.00	238.23	295.20	292.25	284.80
TKT	NC_007320:48754897..48778394	2,302.00	2,872.51	2,543.35	2,512.68	2,476.37
TLL1	NC_007315:1568412..1924045	931.00	461.81	323.94	689.24	526.02
TLR4	NC_007306:112426793..112437806	933.00	1,361.23	1,332.10	824.51	1,112.01
TM2D3	NC_007319:28728494..28773369	890.00	539.29	407.44	589.74	503.58
TM4SF19	NC_007299:72093751..72109873	562.00	1,788.85	2,013.20	1,013.75	1,369.65
TM7SF4	NC_007312:58325569..58332561	102.00	527.89	606.06	162.39	337.67
TM9SF3	NC_007327:18304036..18365208	1,285.00	1,627.77	1,736.36	1,653.19	1,574.76
TMBIM1	NC_007300:110703038..110720587	1,259.00	3,559.43	2,746.51	2,329.74	3,066.29
TMC7	NC_007326:18052226..18112782	1,011.00	1,360.28	1,466.84	1,206.16	1,163.34
TMCO1	NC_007301:3832234..3888168	492.00	370.07	342.75	380.50	328.42
TMED4	NC_007302:79607021..79610652	465.00	551.80	629.91	759.35	746.39
TMED5	NC_007301:53640805..53659323	175.00	247.18	265.76	247.16	263.25
TMEM1	NC_007299:147402628..147477421	681.00	851.14	991.20	802.39	1,054.53
TMEM117	NC_007303:39234967..39833025	297.00	194.93	160.46	229.29	194.57
TMEM129	NC_007304:118768789..118773179	232.00	302.50	399.28	390.33	437.59
TMEM149	NC_007316:45878837..45881461	231.00	121.97	112.78	153.93	150.28

Continued on next page...

Table 1 – continued from previous page

Gene	Location	TCT1	TCT3	TCT4	TCT5	TCT6
TMEM156	NC_007304:60484331..60527484	608.00	938.16	937.51	995.79	1,148.68
TMEM16D	NC_007303:69597248..69945353	216.00	127.42	97.70	167.58	117.40
TMEM188	NC_007316:17487759..17502425	146.00	198.92	208.27	197.20	191.67
TMEM192	NC_007315:150413..179106	257.00	170.85	152.84	198.36	159.09
TMEM199	NC_007317:19725710..19730538	246.00	182.35	195.89	171.63	194.93
TMEM24	NC_007313:28195476..28205370	759.00	659.82	636.70	471.92	625.11
TMEM25	NC_007313:27633290..27637638	55.00	16.56	24.19	29.95	28.74
TMEM33	NC_007304:63221347..63235542	174.00	233.71	270.00	300.46	305.56
TMEM33	NC_007304:63405391..63442994	195.00	267.69	286.71	355.83	338.57
TMEM34	NC_007315:11355470..11395669	267.00	423.76	361.81	373.34	378.51
TMEM41B	NC_007313:42328955..42356710	199.00	305.49	419.00	294.65	371.23
TMEM79	NC_007301:15845808..15851855	151.00	110.95	104.43	100.16	87.11
TMEM97	NC_007317:19691284..19699298	325.00	416.89	441.44	419.76	418.34
TMSB10	NC_007309:51854940..51855951	10,194.00	7,085.55	7,720.50	7,561.24	7,766.81
TMSB4	NC_007299:51153470..51154107	8,313.00	8,765.95	8,993.95	7,445.21	6,537.26
TNF	NC_007324:27531248..27534021	234.00	690.14	531.51	660.63	474.43
TNFAIP8	NC_007305:33597193..33643085	347.00	547.26	508.51	513.48	478.39
TNFAIP8L2	NC_007301:21166314..21169131	267.00	132.72	133.89	118.38	114.85
TNFRSF18	NC_007314:48565346..48568099	211.00	634.89	843.92	684.14	1,143.97
TNFRSF1B	NC_007314:38242240..38275820	6,603.00	5,579.27	4,298.48	5,069.29	5,099.72
TNFRSF21	NC_007324:21146275..21194661	595.00	375.92	480.32	320.77	443.18
TNFRSF25	NC_007314:44110971..44115290	392.00	516.79	536.84	840.20	719.29
TNFRSF4	NC_007314:48557748..48560592	145.00	267.11	236.57	381.64	402.18
TNFRSF6B	NC_007311:54730841..54732350	173.00	101.24	117.92	77.50	74.00
TNFRSF9	NC_007314:42581703..42600709	154.00	634.10	1,101.73	699.67	1,499.75
TNFSF10	NC_007299:97101994..97118087	536.00	1,377.42	1,334.75	1,406.50	1,058.67
TNFSF13B	NC_007310:81686381..81716159	142.00	460.86	366.11	200.85	373.38
TNFSF14	NC_007305:16382783..16387011	259.00	696.85	722.49	438.26	873.72
TNFSF9	NC_007305:16505560..16524677	97.00	346.60	391.56	165.79	316.11
TNIK	NC_007299:98270087..98676213	7,162.00	6,815.10	8,108.61	7,505.16	6,799.44
TNIP1	NC_007305:61911982..61956302	7,139.00	8,524.56	6,148.07	5,613.69	5,314.64
TNPO2	NC_007305:11057906..11074828	971.00	1,253.24	1,298.57	1,425.30	1,511.33
TNRC6C	NC_007317:55626989..55669028	617.00	431.60	433.27	454.22	474.03
TNS4	NC_007317:41961260..41980523	12.00	0.74	0.68	2.07	1.27
TOMM34	NC_007311:74113802..74133797	295.00	417.49	443.77	481.31	459.89
TOP1	NC_007311:70393933..70489300	1,405.00	2,228.09	2,180.15	1,797.97	1,732.94
TOP2A	NC_007317:41892308..41917595	158.00	83.08	87.74	105.46	80.38
TOR1AIP2	NC_007314:58672055..58679824	94.00	210.84	191.08	150.88	171.56
TPCN2	NC_007330:48183992..48227747	372.00	601.95	511.69	500.02	683.11
TPPP	NC_007318:75497764..75516628	79.00	38.54	37.32	19.14	41.42
TPR	NC_007314:65154622..65217958	1,661.00	2,045.76	2,124.60	2,184.81	2,055.22
TPT1	NC_007310:14460212..14463752	21,739.00	20,711.32	22,826.87	24,818.56	23,991.70
TRA1	NC_007303:72625306..72644045	3,611.00	5,724.89	5,197.52	4,627.85	4,345.07
TRA2A	NC_007302:33442274..33460830	1,003.00	1,198.81	1,166.90	1,214.41	1,239.81
TRAF1	NC_007306:115684465..115704716	811.00	1,281.34	1,237.68	1,159.44	1,405.10
TRAF2	NC_007309:109360198..109384668	815.00	1,016.66	1,024.59	1,214.87	1,266.58
TRAF3IP1	NC_007301:125297340..125356965	320.00	204.35	141.92	247.81	243.25
TRAF3IP3	NC_007314:71690226..71710792	1,246.00	855.24	1,001.64	990.34	904.91
TRAF4	NC_007317:20053389..20059468	238.00	387.48	421.71	528.66	506.94
TRAF7	NC_007326:2211073..2220050	1,204.00	1,465.70	1,411.93	1,682.78	1,653.61
TREM1	NC_007324:15763070..15778191	1,683.00	1,468.52	987.19	1,018.34	1,035.79
TRIB2	NC_007309:87937658..87965774	206.00	126.44	125.00	150.30	128.73

Continued on next page...

Table 1 – continued from previous page

Gene	Location	TCT1	TCT3	TCT4	TCT5	TCT6
TRIM21	NC_007313:50068098..50077066	349.00	446.23	475.15	475.97	461.45
TRIM6	NC_007313:47231143..47248397	402.00	767.45	555.50	770.62	546.07
TRIP12	NC_007300:121961854..122069022	2,511.00	3,299.51	3,124.65	2,838.51	3,186.39
TRMT6	NC_007311:48346637..48357172	181.00	344.92	325.85	265.94	276.09
trp	NC_007331:38036838..38244940	303.00	124.47	133.73	231.97	167.91
TRPV1	NC_007317:24379195..24402066	76.00	40.44	44.89	33.77	33.54
TRPV2	NC_007317:34102769..34121101	1,172.00	1,050.69	835.26	938.09	1,329.69
TRRAP	NC_007326:39046246..39133912	1,304.00	1,457.26	1,462.13	1,842.87	1,913.98
TSKU	NC_007313:55760378..55773771	44.00	11.59	12.09	11.33	6.39
TSPAN11	NC_007303:113355974..113418216	64.00	20.48	9.27	13.32	13.19
TSR1	NC_007317:23387832..23396855	470.00	599.97	640.05	687.20	663.90
TTC29	NC_007315:12236411..12506561	254.00	152.94	122.41	163.63	124.95
TTC7A	NC_007309:30552508..30673544	683.00	435.50	505.97	526.42	541.39
TTF2	NC_007301:28476314..28529876	286.00	222.61	218.90	224.40	204.54
TUBA1	NC_007303:33598568..33610436	5,243.00	3,547.34	3,900.28	4,460.97	4,430.81
TUBA1B	NC_007303:33737403..33741020	8,295.00	7,233.47	8,836.79	9,967.42	8,942.09
TUBA3D	NC_007299:41676636..41716671	2,125.00	1,934.56	2,708.00	2,455.61	2,487.86
TUBA4A	NC_007300:111601055..111604852	689.00	590.43	882.32	1,067.52	992.32
TUBB	NC_007324:28220824..28224415	4,753.00	4,188.91	4,487.22	6,075.54	5,507.38
TUBB1	NC_007311:58082032..58086997	50.00	90.49	108.92	94.63	97.87
TUBB2A	NC_007324:51484247..51488127	1,331.00	1,187.30	1,470.04	1,813.18	1,676.70
TULP3	NC_007303:113714896..113745381	106.00	65.99	55.63	67.31	69.91
TWISTNB	NC_007302:29683182..29694034	151.00	414.43	285.32	197.79	280.76
TXN	NC_007306:104842172..104852693	1,194.00	2,081.04	2,467.25	1,330.20	1,588.28
TXNDC5	NC_007324:48380034..48405070	1,590.00	958.45	1,071.24	946.30	1,009.09
TXNIP	NC_007301:23256754..23259469	1,904.00	1,108.11	1,188.90	1,202.63	1,098.83
TXNRD1	NC_007303:72925128..72988224	5,899.00	12,402.41	10,483.48	6,876.57	8,306.95
TYK2	NC_007305:13340395..13368736	1,744.00	3,893.79	3,217.40	2,608.06	3,000.35
UAP1	NC_007301:7498066..7529834	594.00	888.32	994.77	710.91	905.26
UBA1	NC_007331:56602559..56621182	3,688.00	4,225.06	4,746.05	5,028.59	5,113.96
UBA2	NC_007316:44579229..44609887	866.00	1,102.64	1,156.39	1,123.59	1,131.12
UBA5	NC_007299:139078773..139111360	475.00	625.71	759.28	641.76	703.85
UBA52	NC_007305:4530705..4533091	7,570.00	6,451.34	8,466.26	9,675.44	8,436.52
UBA6	NC_007304:86000163..86086979	400.00	517.15	589.24	674.45	764.85
UBA7	NC_007320:51327194..51336258	1,539.00	2,122.73	2,042.96	2,356.39	2,290.47
UBC	NC_007315:53717897..53721455	50,182.00	73,439.73	88,695.56	64,489.26	84,857.63
UBD	NC_007324:29074282..29076743	11,666.00	10,386.54	13,176.62	15,267.65	9,932.38
UBE2D3	NC_007304:23627693..23655008	2,076.00	2,757.30	3,011.29	3,117.18	3,062.22
UBE2J1	NC_007307:63475497..63501983	540.00	764.66	797.07	721.00	699.99
UBE2K	NC_007304:61155886..61230741	551.00	661.35	684.22	732.55	746.93
UBE2V1	NC_007311:79125938..79153877	954.00	1,120.76	1,304.31	1,110.27	1,094.03
UBE4A	NC_007313:27471849..27514842	700.00	925.58	895.01	841.05	931.68
UBQLN1	NC_007306:81071414..81120935	756.00	999.05	1,121.88	980.61	1,037.30
UBR4	NC_007300:138076948..138214084	3,596.00	5,390.08	5,339.68	5,830.70	6,941.76
UBR5	NC_007312:60168917..60306581	1,920.00	2,375.91	2,545.95	2,237.68	2,628.93
UBTD1	NC_007327:20535406..20586025	233.00	163.17	141.71	98.47	116.91
UBXD7	NC_007299:72113620..72188908	199.00	353.14	367.61	285.91	354.82
UCKL1	NC_007311:54567620..54578855	325.00	465.86	546.39	622.44	660.40
UFC1	NC_007301:9264776..9268472	649.00	488.75	490.02	474.16	426.50
UGCG	NC_007306:106465910..106502775	360.00	525.78	477.96	485.18	533.74
UGP2	NC_007309:64076656..64132064	598.00	866.56	1,000.03	817.49	733.77
UHRF1BP1L	NC_007303:68995840..69089832	246.00	406.54	407.93	362.52	399.68

Continued on next page...

Table 1 – continued from previous page

Gene	Location	TCT1	TCT3	TCT4	TCT5	TCT6
ULBP4	NC_007307:87754055..87759251	15.00	64.44	70.62	82.50	79.86
ULK4	NC_007320:14183692..14707594	823.00	499.85	378.55	642.78	506.50
UNC119	NC_007317:19898765..19904329	822.00	604.39	563.19	587.25	560.74
UNC84B	NC_007303:117434638..117452728	1,838.00	1,092.95	1,263.01	1,220.07	1,281.49
UNC93B1	NC_007330:47476548..47486602	2,564.00	2,748.97	3,242.80	3,442.65	3,635.03
UNG	NC_007315:67256479..67266659	176.00	120.11	114.97	120.22	107.28
UPF2	NC_007311:10797334..10892842	697.00	996.23	967.66	898.17	825.89
UPP1	NC_007302:8814998..8843152	1,339.00	1,644.46	1,501.97	1,172.91	1,179.35
UQCRH	NC_007301:106693271..106702634	820.00	618.66	707.59	664.08	705.88
USO1	NC_007304:93784670..93865781	884.00	1,224.91	1,419.46	1,145.57	1,185.47
USP14	NC_007325:36272455..36307654	389.00	643.00	632.52	577.81	602.39
USP16	NC_007299:6408561..6434422	470.00	642.85	702.50	566.44	578.13
USP18	NC_007303:81760098..81779866	394.00	520.31	586.93	573.57	614.69
USP25	NC_007299:20900545..21062522	1,868.00	2,417.13	2,452.98	2,059.79	2,289.36
USP34	NC_007309:61847482..62102275	2,342.00	2,625.57	2,782.83	2,867.45	3,052.37
USP4	NC_007320:51694636..51734082	1,429.00	2,081.89	1,946.40	1,766.41	2,058.27
USP40	NC_007301:120474367..120553752	544.00	440.30	436.47	455.95	452.95
USP7	NC_007326:8617200..8649832	2,398.00	2,775.38	2,786.99	2,771.25	2,852.27
USPL1	NC_007310:29994779..30018019	356.00	466.63	555.45	436.15	530.35
UTP14A	NC_007319:56780290..56782967	268.00	209.35	212.42	155.56	191.11
UTP15	NC_007318:8415650..8434419	184.00	293.17	337.40	264.55	295.59
VAMP7	NC_007331:24189598..24366903	367.00	609.06	672.68	547.17	604.79
VANGL1	NC_007301:30109639..30161651	35.00	15.45	12.05	8.46	16.13
VAV1	NC_007305:16104150..16244285	1,080.00	1,452.08	1,346.22	1,204.25	1,239.06
VAV3	NC_007301:38123434..38579022	1,404.00	1,256.28	1,172.17	1,078.40	1,003.98
VCAN	NC_007305:84420951..84537508	1,431.00	2,051.33	2,277.48	1,036.93	1,895.89
VCP	NC_007306:61976874..61991606	6,750.00	9,317.11	9,231.73	8,800.59	9,385.29
VCPIP1	NC_007312:30764507..30790163	809.00	1,507.89	1,532.24	1,161.76	1,440.35
VDAC1	NC_007305:44846725..44872440	543.00	661.28	741.54	700.75	842.19
VIM	NC_007311:31476151..31484080	5,810.00	9,329.84	8,519.88	7,188.63	9,232.82
VPRBP	NC_007320:50281998..50344884	655.00	750.59	826.84	908.90	900.05
VPS25	NC_007317:44165168..44169926	667.00	539.68	531.90	579.67	488.59
VPS33A	NC_007315:55801198..55830768	2,684.00	2,373.93	2,278.41	2,131.16	1,831.61
VPS37B	NC_007315:55300058..55336877	882.00	579.68	557.94	575.03	566.86
VPS54	NC_007309:64133430..64209402	691.00	843.64	879.49	985.39	909.70
VSTM1	NC_007316:62072238..62091975	329.00	238.21	230.83	83.70	228.69
VSTM2L	NC_007311:67394034..67432571	654.00	237.41	289.85	353.23	390.67
VWF	NC_007303:111087625..111209708	515.00	2,145.70	1,974.03	858.26	1,924.55
WAC	NC_007311:35997232..36076317	1,385.00	1,825.83	1,906.51	1,940.73	1,827.63
WARS	NC_007319:65341132..65363589	10,703.00	14,817.42	17,797.60	16,871.30	21,990.00
WASL	NC_007302:91021741..91101028	209.00	412.87	115.59	150.76	153.59
WC1.2	NC_007303:9769175..9830368	2,540.00	1,124.80	1,696.61	1,311.93	2,091.41
WC1.3	NC_007303:109996449..110056386	2,304.00	1,177.14	1,526.88	1,406.59	1,890.25
WDFY1	NC_007300:116551435..116603551	414.00	775.74	555.56	728.84	679.31
WDR1	NC_007304:109677057..109718082	5,737.00	5,135.02	5,240.74	6,789.00	7,222.19
WDR26	NC_007314:24581751..24615167	896.00	1,139.79	1,260.20	1,298.02	1,342.96
WDR3	NC_007301:27529412..27568528	1,613.00	788.60	767.83	1,028.97	861.39
WDR33	NC_007300:4729973..4853218	1,245.00	1,484.10	1,541.45	1,486.02	1,615.34
WDR37	NC_007311:46457974..46492700	831.00	1,135.10	1,177.39	1,420.27	1,463.22
WDR40A	NC_007306:79715552..79752274	653.00	792.09	847.50	752.68	821.80
WDR42A	NC_007301:10429315..10431176	184.00	120.78	126.52	131.39	140.66
WDR43	NC_007309:72863134..72902239	490.00	570.81	631.72	739.77	578.58

Continued on next page...

Table 1 – continued from previous page

Gene	Location	TCT1	TCT3	TCT4	TCT5	TCT6
WDR67	NC_007312:16185133..16251964	1,616.00	1,954.95	2,114.12	2,749.88	769.48
WDR7	NC_007325:58237350..58597393	608.00	909.59	771.01	790.80	817.57
WDR75	NC_007300:7273030..7317660	446.00	545.51	643.88	677.36	570.47
WDR81	NC_007317:22913052..22922277	537.00	386.75	399.27	437.68	405.68
WHSC1	NC_007304:118987537..119029770	434.00	543.67	607.71	687.16	931.77
WNK1	NC_007303:114543686..114618252	1,901.00	2,335.31	2,056.05	2,514.47	2,522.75
WNT5A	NC_007320:46576092..46592148	181.00	103.73	70.50	83.78	105.93
WWOX	NC_007316:4120567..5143648	636.00	417.48	400.75	429.15	404.39
XDH	NC_007309:14805151..14865238	4,415.00	3,990.47	3,355.77	2,654.83	3,092.42
XPNPEP1	NC_007327:30647424..30698893	587.00	693.09	719.23	856.07	794.86
XPO1	NC_007309:62128385..62170957	1,023.00	1,478.78	1,638.45	1,597.59	1,668.51
XPO4	NC_007310:36043103..36113615	188.00	245.68	258.87	277.08	316.99
XPO6	NC_007326:27422001..27493645	2,421.00	2,925.49	2,739.93	3,024.91	3,055.85
XRCC1	NC_007316:51481727..51500622	211.00	125.24	130.07	126.26	163.35
XRN2	NC_007311:40741380..40804316	1,612.00	2,310.51	2,578.92	2,222.99	2,161.01
YIPF1	NC_007301:98965588..98991260	133.00	60.39	77.65	72.85	71.62
YIPF3	NC_007324:17670945..17675216	561.00	429.01	436.08	448.22	396.59
YIPF5	NC_007305:54889552..54905312	357.00	462.44	518.91	449.00	474.55
YIPF6	NC_007331:50956764..50982150	194.00	263.82	291.77	257.40	248.16
YME1L1	NC_007311:16969149..16997943	933.00	1,301.73	1,497.84	1,304.43	1,311.25
YPEL1	NC_007315:75437808..75441571	71.00	35.81	22.32	24.04	18.29
YTHDC1	NC_007304:86793136..86831235	805.00	1,134.58	1,195.19	1,405.18	1,438.21
YTHDF1	NC_007311:55015239..55028008	267.00	333.27	418.57	416.05	379.53
YTHDF3	NC_007312:28026852..28060347	576.00	750.16	812.80	688.90	755.29
YWHAQ	NC_007309:90637000..90668462	851.00	1,165.27	1,226.98	1,308.72	1,054.88
ZAP70	NC_007309:3331029..3370822	5,954.00	3,612.65	6,274.76	5,309.66	8,635.04
ZBP1	NC_007311:59325694..59337686	538.00	957.71	904.65	670.57	786.91
ZBTB38	NC_007299:129739602..129746475	405.00	568.28	560.08	511.10	508.66
ZBTB40	NC_007300:134779088..134816314	481.00	583.21	610.16	748.98	863.22
ZBTB44	NC_007330:38110184..38131679	211.00	354.99	398.38	386.78	455.67
ZC3H7A	NC_007326:11502606..11529156	788.00	936.93	964.03	951.77	961.84
ZCCHC6	NC_007306:83595910..83653946	736.00	993.72	928.63	924.91	1,001.39
ZEB1	NC_007311:33678105..33744568	209.00	318.94	295.25	318.97	376.35
ZEB2	NC_007300:54402583..54492543	184.00	413.14	325.15	242.45	307.00
ZFP36L2	NC_007309:26868770..26873023	2,514.00	2,037.75	2,234.45	1,902.59	1,870.90
ZFP91	NC_007313:82761334..82789866	878.00	1,188.01	1,119.63	1,052.32	1,018.70
ZFPM1	NC_007316:12755595..12772268	54.00	99.05	112.78	89.38	137.22
ZFR	NC_007318:43705858..43782555	573.00	732.17	748.69	663.77	714.04
ZMYM4	NC_007301:117543168..117706789	3,390.00	2,516.07	2,525.08	2,705.75	2,524.15
ZMYM5	NC_007310:36575036..36601266	272.00	365.22	397.11	406.83	396.24
ZMYND11	NC_007311:47012264..47072584	821.00	1,010.57	1,013.56	1,103.75	1,009.69
ZNF292	NC_007307:64480034..64575552	475.00	702.57	620.52	704.91	700.68
ZNF295	NC_007299:145073844..145092111	94.00	163.71	178.99	138.34	182.79
ZNF414	NC_007305:15572600..15576266	284.00	194.28	198.38	183.44	175.36
ZNF629	NC_007326:28734580..28759760	163.00	118.91	110.60	83.33	94.18
ZNF639	NC_007299:90054206..90059557	88.00	127.71	155.90	125.28	168.72
ZNF773	NC_007316:65004521..65011856	108.00	69.58	74.44	48.94	72.60
ZNF774	NC_007319:22006014..22015357	43.00	76.94	79.40	92.46	78.81
ZNF777	NC_007302:116739320..116761873	272.00	199.80	199.73	199.17	217.52
ZNF821	NC_007316:38310297..38327376	55.00	31.11	24.81	18.44	22.51
ZNFX1	NC_007311:78362167..78386046	2,162.00	4,136.34	4,158.67	4,293.44	4,002.96
ZSWIM4	NC_007305:10106531..10128019	180.00	302.82	272.88	246.38	313.03

Continued on next page...

Table 1 – continued from previous page

Gene	Location	TCT1	TCT3	TCT4	TCT5	TCT6
ZYX	NC_007302:110832093..110841072	2,546.00	2,230.60	2,222.60	1,728.94	2,345.49
