# Supplementary material for: Accurate Diagnostics for Bovine tuberculosis Based on High-Throughput Sequencing
Source: PLoS One. 2012 Nov 30;7(11):e50147. doi: 10.1371/journal.pone.0050147 (PMC3511461; doi:10.1371/journal.pone.0050147)
Supplement: Supporting Information S5 — Exons changing inclusion. (PDF) [file pone.0050147.s005.pdf]

Supporting Information S5

Accurate diagnostics for *Bovine tuberculosis* based on high-throughput sequencing

Alexander Churbanov and Brook Milligan

Exons changing inclusion

Heatmap for exons changing inclusion at the significance level of 0.01 could be seen in Figure 1.

Table 1: List of exons changing inclusion to the final transcript with statistical significance equal or less than 0.01 with the Fischer test comparing inclusion levels of TCT1 with TCT3, TCT4, TCT5 and TCT6. Number of Illumina reads mapped to the exon is normalized by the number of reads mapped against the containing gene locus. In red bold color we highlight the control counts that were changed from 0 to 1 to avoid division by zero when calculating mean ratio value.

Location	TCT1	TCT3	TCT4	TCT5	TCT6	Mean ratio	STD
NC_007305:53436461..53436562	**1**	18.44	32.10	13.11	18.41	20.52	8.12
NC_007327:15158492..15158653	1	15.20	17.90	11.17	9.70	13.49	3.75
NC_007303:79821766..79822101	**1**	10.82	11.94	10.81	19.32	13.22	4.10
NC_007303:79905240..79905575	**1**	12.33	12.99	8.65	18.16	13.03	3.92
NC_007303:61590385..61590444	**1**	13.23	6.84	7.13	14.43	10.41	3.98
NC_007309:101947636..101947774	**1**	10.53	11.23	9.23	9.14	10.03	1.02
NC_007301:97801290..97801351	1	11.94	10.03	8.64	9.50	10.03	1.40
NC_007309:101948641..101948750	**1**	6.87	6.66	12.30	12.19	9.51	3.17
NC_007316:21501886..21502029	3	14.82	17.21	25.10	42.23	8.28	4.13
NC_007299:146164843..146164927	**1**	7.80	5.90	6.84	9.04	7.40	1.34
NC_007306:107820632..107820704	2.00	11.21	14.70	13.40	19.37	7.34	1.72
NC_007327:16228446..16228574	2.00	10.75	20.66	10.96	10.77	6.64	2.46
NC_007324:7738320..7738372	2.00	11.96	13.56	12.02	13.41	6.37	0.43
NC_007305:53878499..53878609	5.00	29.96	26.26	29.14	22.50	5.39	0.67
NC_007303:48550655..48553746	24.00	153.48	104.59	93.66	71.56	4.41	1.44
NC_007305:53451542..53451677	7.00	32.27	32.10	27.68	30.68	4.38	0.30
NC_007317:37726921..37727010	5.00	19.39	21.52	22.41	21.79	4.26	0.26

Continued on next page...

Table 1 – continued from previous page

Location	TCT1	TCT3	TCT4	TCT5	TCT6	Mean ratio	STD
NC_007301:117928936..117929108	5.00	21.45	18.66	17.07	23.83	4.05	0.60
NC_007310:69855307..69855556	24.00	76.70	75.60	87.66	96.85	3.51	0.42
NC_007299:156214950..156215067	11.00	29.74	38.65	37.52	47.40	3.48	0.66
NC_007310:69859427..69859516	23.00	53.31	58.92	95.98	112.10	3.48	1.24
NC_007302:9883530..9883830	10.00	33.36	39.46	26.95	31.87	3.29	0.52
NC_007316:21902308..21904695	44.00	94.87	101.82	196.64	174.97	3.23	1.17
NC_007301:27554366..27554463	9.00	28.73	26.72	24.47	31.41	3.09	0.33
NC_007305:53893516..53895494	73.00	184.38	338.49	154.42	212.71	3.05	1.11
NC_007299:156215307..156215394	9.00	26.52	24.27	27.42	26.40	2.91	0.15
NC_007302:103031916..103032044	12.00	29.19	31.06	38.05	40.96	2.90	0.47
NC_007324:28964769..28965899	12.00	28.78	29.69	34.08	44.52	2.86	0.60
NC_007310:69858213..69858376	48.00	91.66	104.50	149.72	179.20	2.73	0.84
NC_007314:48270036..48270172	32.00	89.14	69.93	85.36	94.52	2.65	0.33
NC_007305:19869306..19870088	15.00	37.78	41.92	40.09	36.63	2.61	0.16
NC_007301:27552286..27552516	30.00	61.76	70.31	70.35	109.95	2.60	0.72
NC_007317:43767938..43767997	12.00	28.29	29.35	33.44	33.13	2.59	0.22
NC_007310:69882568..69882678	35.00	98.21	90.05	74.86	73.21	2.40	0.35
NC_007314:48261864..48262002	25.00	56.13	53.48	63.57	65.97	2.39	0.24
NC_007310:69881897..69882017	50.00	163.69	118.95	99.18	89.22	2.36	0.66
NC_007314:48273527..48273648	52.00	118.85	87.42	130.77	137.84	2.28	0.43
NC_007315:47670853..47670990	15.00	31.96	32.79	32.51	36.38	2.23	0.13
NC_007320:45225884..45228124	201.00	542.09	578.69	311.23	349.66	2.22	0.67
NC_007309:101963145..101964559	21.00	48.52	42.43	49.73	43.17	2.19	0.18
NC_007316:63321859..63322161	29.00	60.76	67.93	66.70	51.90	2.13	0.25
NC_007324:27464520..27464605	24.00	49.57	47.57	53.38	46.19	2.05	0.13
NC_007324:27254608..27255972	26.00	52.49	52.44	53.11	52.37	2.02	0.01
NC_007310:76857976..76860443	279.00	564.78	740.46	421.67	513.98	2.01	0.48
NC_007306:72635292..72635444	24.00	44.19	49.03	54.14	45.26	2.01	0.19
NC_007305:4266409..4266490	28.00	50.87	50.83	57.80	64.52	2.00	0.23
NC_007310:69863709..69863933	60.00	133.75	97.83	120.29	124.30	1.98	0.25
NC_007299:59197673..59204103	108.00	176.72	255.27	192.67	226.93	1.97	0.33
NC_007324:25335210..25338847	133.00	288.82	239.57	204.51	300.39	1.94	0.33
NC_007316:63622916..63623221	60.00	91.97	91.85	144.93	137.19	1.94	0.48
NC_007309:21378811..21379396	21.00	41.11	40.83	40.32	39.11	1.92	0.04
NC_007324:27846487..27846534	70.00	135.76	112.47	184.27	102.86	1.91	0.52
NC_007316:63368196..63368382	45.00	96.43	89.45	78.52	76.87	1.90	0.21
NC_007305:17039019..17039168	43.00	68.93	77.17	82.70	87.71	1.84	0.19
NC_007309:21378036..21379396	36.00	57.10	72.87	66.41	57.73	1.76	0.21
NC_007316:1156998..1157111	49.00	77.04	76.16	99.57	88.91	1.74	0.23
NC_007301:27529412..27530145	93.00	186.72	188.44	136.63	135.32	1.74	0.32
NC_007320:22198272..22199611	122.00	198.44	210.05	200.65	203.58	1.67	0.04
NC_007302:9964510..9967689	196.00	327.17	316.38	324.57	309.51	1.63	0.04
NC_007311:66525129..66525224	94.00	135.38	154.23	156.91	158.92	1.61	0.12
NC_007320:44174159..44176064	74.00	111.68	120.81	114.85	119.49	1.58	0.06
NC_007299:147220851..147221016	101.00	168.74	141.71	139.67	163.56	1.52	0.15
NC_007310:11794482..11794581	77.00	110.87	113.04	113.33	128.11	1.51	0.10
NC_007318:3703836..3706287	293.00	428.96	482.64	411.87	433.41	1.50	0.10
NC_007316:62246449..62246698	224.00	280.30	283.82	377.84	378.40	1.47	0.25
NC_007305:15537067..15537258	144.00	217.88	192.24	193.58	224.09	1.44	0.11
NC_007307:29712480..29713731	95.00	131.26	137.90	132.62	131.56	1.40	0.03
NC_007326:2840499..2840506	150.00	217.74	196.68	197.13	219.84	1.39	0.08
NC_007324:7740088..7740133	87.00	43.65	163.71	129.77	139.05	1.37	0.60

Continued on next page...

Table 1 – continued from previous page

Location	TCT1	TCT3	TCT4	TCT5	TCT6	Mean ratio	STD
NC_007303:33602017..33602239	930.00	1,041.43	1,198.35	1,393.57	1,434.45	1.36	0.20
NC_007313:42438871..42439528	197.00	252.49	268.88	256.96	276.66	1.34	0.06
NC_007328:46213721..46213903	974.00	763.93	1,381.23	1,531.97	1,504.92	1.33	0.37
NC_007330:27773627..27773916	3,674.00	3,988.57	5,244.67	5,244.61	4,903.86	1.32	0.16
NC_007303:60745307..60745485	198.00	249.06	267.83	256.68	256.18	1.30	0.04
NC_007310:18628264..18630838	141.00	176.45	202.61	175.58	177.49	1.30	0.09
NC_007305:10885613..10885707	761.00	867.61	880.86	1,062.40	1,093.52	1.28	0.16
NC_007301:17718555..17718624	255.00	327.75	307.24	321.06	335.05	1.27	0.05
NC_007305:18627940..18628121	1,241.00	1,458.52	1,380.11	1,651.56	1,774.48	1.26	0.14
NC_007305:18626887..18627101	925.00	1,050.58	1,105.11	1,220.10	1,282.66	1.26	0.11
NC_007312:64683940..64685663	429.00	493.62	545.07	589.64	524.80	1.25	0.09
NC_007305:20628204..20628336	824.00	923.55	964.39	1,120.52	1,124.39	1.25	0.13
NC_007306:5598746..5599299	351.00	417.43	441.44	446.84	450.00	1.25	0.04
NC_007310:14463318..14463391	1,301.00	1,098.98	1,548.63	1,714.31	2,140.41	1.25	0.33
NC_007300:133636935..133637066	1,237.00	1,468.48	1,492.27	1,544.87	1,630.05	1.24	0.06
NC_007316:48626367..48626467	284.00	186.84	396.10	423.83	400.21	1.24	0.39
NC_007330:27711117..27711208	946.00	822.05	1,366.10	1,331.84	1,165.57	1.24	0.26
NC_007306:81414522..81414829	448.00	365.12	545.08	667.88	638.81	1.24	0.30
NC_007330:42118584..42118904	58,325.00	62,960.22	70,760.40	75,148.90	77,941.45	1.23	0.11
NC_007301:21681057..21681921	316.00	371.35	394.44	398.98	380.29	1.22	0.04
NC_007326:40637512..40637693	6,210.00	5,418.19	6,923.30	9,052.21	8,643.00	1.21	0.27
NC_007316:55361285..55361410	1,120.00	1,289.89	998.08	1,397.41	1,725.39	1.21	0.27
NC_007311:23079645..23080313	368.00	444.24	427.82	445.16	453.32	1.20	0.03
NC_007309:2654129..2656737	193.00	223.24	236.54	228.17	229.35	1.19	0.03
NC_007316:55547408..55547554	889.00	792.86	1,107.90	1,127.04	1,196.59	1.19	0.20
NC_007317:15403977..15404537	235.00	280.88	271.15	278.69	285.05	1.19	0.02
NC_007317:15608869..15609431	232.00	275.59	266.89	274.69	281.42	1.18	0.03
NC_007316:55360511..55360657	887.00	781.75	1,120.80	1,097.58	1,197.95	1.18	0.21
NC_007303:110692689..110695208	195.00	229.22	225.03	229.24	239.29	1.18	0.03
NC_007301:80469832..80470943	457.00	548.48	527.51	548.02	523.25	1.17	0.03
NC_007326:27948994..27949139	1,077.00	1,230.60	1,262.99	1,229.54	1,328.83	1.17	0.04
NC_007326:2003555..2003662	3,841.00	4,304.47	4,690.61	4,478.81	4,511.84	1.17	0.04
NC_007309:108186390..108186469	883.00	1,009.97	1,020.02	1,092.80	1,008.68	1.17	0.05
NC_007317:26824096..26824443	3,354.00	3,797.19	3,836.14	4,069.78	3,983.35	1.17	0.04
NC_007317:57543515..57545886	3,590.00	3,939.99	4,098.26	4,333.90	4,309.96	1.16	0.05
NC_007304:23654140..23655008	724.00	801.82	871.27	879.01	802.40	1.16	0.06
NC_007304:92061614..92062287	5,376.00	6,138.34	6,552.46	6,408.16	5,791.31	1.16	0.06
NC_007311:75522838..75522993	4,255.00	4,724.60	4,666.11	4,884.69	5,418.97	1.16	0.08
NC_007305:75279352..75280574	416.00	472.18	505.41	469.87	477.00	1.16	0.04
NC_007311:55653079..55654320	223.00	257.77	263.54	255.54	254.51	1.16	0.02
NC_007330:27727321..27727412	1,618.00	1,419.78	2,013.91	2,006.83	2,004.61	1.15	0.18
NC_007316:55894736..55894795	1,434.00	1,275.36	1,694.93	1,853.00	1,773.12	1.15	0.18
NC_007304:92073329..92073927	22,080.00	24,355.33	25,857.05	25,542.32	24,828.46	1.14	0.03
NC_007328:21723592..21725210	499.00	560.88	575.50	578.17	555.19	1.14	0.02
NC_007306:7458358..7458443	957.00	817.63	1,148.75	1,240.04	1,134.42	1.13	0.19
NC_007304:91878905..91880020	69,060.00	75,894.67	76,786.93	79,360.74	79,564.70	1.13	0.03
NC_007324:28555393..28555668	3,000.00	3,172.56	3,276.82	3,215.12	3,737.24	1.12	0.09
NC_007319:67024629..67025429	1,474.00	1,366.39	1,648.26	1,837.00	1,656.87	1.10	0.13
NC_007301:84657115..84659396	1,170.00	1,279.65	1,283.73	1,309.69	1,278.79	1.10	0.01
NC_007301:21668314..21671073	2,432.00	2,561.94	2,655.20	2,659.55	2,653.99	1.08	0.02
NC_007314:71804284..71804974	1,765.00	1,873.44	1,879.86	1,966.06	1,870.03	1.07	0.03
NC_007304:91792160..91792295	9,305.00	8,448.22	8,839.21	10,364.08	12,104.70	1.07	0.18

Continued on next page...

Table 1 – continued from previous page

Location	TCT1	TCT3	TCT4	TCT5	TCT6	Mean ratio	STD
NC_007324:29074282..29074459	312.00	232.87	402.31	435.63	249.59	1.06	0.33
NC_007331:81984917..81985358	8,387.00	8,026.65	8,592.03	9,332.68	9,528.55	1.06	0.08
NC_007309:48254158..48254356	8,082.00	7,648.66	8,707.46	8,889.45	8,937.38	1.06	0.08
NC_007330:27684332..27684612	15,232.00	15,761.29	15,905.57	15,792.82	16,831.92	1.06	0.03
NC_007304:91928804..91929555	30,630.00	33,150.17	34,478.04	32,857.34	28,558.42	1.05	0.08
NC_007304:92072400..92072483	7,309.00	7,699.43	6,790.05	7,779.11	8,502.80	1.05	0.10
NC_007324:8764478..8764627	2,794.00	3,391.68	3,186.42	2,562.51	2,613.95	1.05	0.15
NC_007309:26868770..26872138	2,114.00	2,191.08	2,215.97	2,273.74	2,200.71	1.05	0.02
NC_007324:28656436..28656468	733.00	634.86	859.02	494.45	1,077.22	1.05	0.35
NC_007303:108852130..108854809	9,521.00	9,998.70	9,757.76	10,008.63	9,982.69	1.04	0.01
NC_007324:27599840..27599943	215.00	308.47	159.79	267.62	156.46	1.04	0.36
NC_007307:12614772..12615006	6,454.00	5,797.94	6,813.73	7,042.91	7,043.94	1.03	0.09
NC_007307:99827403..99827605	2,712.00	2,149.28	2,387.89	3,304.19	3,303.46	1.03	0.22
NC_007317:13822062..13822206	6,021.00	6,315.98	5,594.59	6,229.22	6,339.82	1.02	0.06
NC_007314:16621533..16622248	120.00	66.72	164.33	73.45	183.26	1.02	0.50
NC_007304:91793092..91794218	23,118.00	24,835.24	24,521.78	23,998.82	20,287.78	1.01	0.09
NC_007307:12613786..12614669	9,449.00	9,862.09	10,592.93	9,119.92	8,487.73	1.01	0.10
NC_007317:34140348..34141411	22,019.00	22,085.70	22,132.66	22,151.39	22,130.64	1.00	0.00
NC_007299:129199748..129200420	339.00	445.16	408.13	284.74	221.98	1.00	0.31
NC_007315:53718892..53721455	50,055.00	49,980.13	50,017.09	50,109.24	50,104.86	-1.00	0.00
NC_007324:29075711..29076743	11,165.00	11,041.52	10,980.55	10,944.34	11,001.32	-1.02	0.00
NC_007307:99818144..99818944	5,935.00	6,611.35	6,330.47	5,327.81	5,222.56	-1.02	0.12
NC_007330:45143038..45143182	1,236.00	1,030.44	1,082.43	1,382.68	1,447.30	-1.02	0.17
NC_007301:18499228..18499640	3,614.00	3,843.66	3,450.04	3,393.13	3,467.77	-1.02	0.06
NC_007330:42119001..42119126	31,231.00	36,436.80	27,272.81	29,007.84	29,098.89	-1.04	0.13
NC_007304:91927728..91927851	15,046.00	13,756.71	13,292.92	14,195.90	16,987.24	-1.04	0.11
NC_007330:27708473..27708766	5,459.00	5,745.18	4,977.94	4,930.36	5,223.74	-1.05	0.07
NC_007309:48256895..48256956	2,933.00	3,830.06	2,629.18	2,621.81	2,435.57	-1.05	0.19
NC_007328:46215381..46217229	5,337.00	5,621.72	4,925.78	4,733.59	4,832.03	-1.07	0.08
NC_007301:18513454..18513673	2,010.00	2,179.10	2,186.35	1,698.52	1,596.83	-1.07	0.18
NC_007319:67027722..67028042	943.00	1,242.50	822.31	752.61	829.70	-1.07	0.22
NC_007304:92073329..92073356	6,501.00	7,206.97	5,737.98	5,587.03	5,830.67	-1.08	0.12
NC_007320:12896775..12897020	3,051.00	2,700.52	2,830.71	2,850.71	2,880.45	-1.08	0.03
NC_007330:51573714..51573836	868.00	979.50	754.25	759.39	746.33	-1.09	0.13
NC_007307:7010672..7011280	3,435.00	3,257.52	3,274.87	3,157.64	2,891.33	-1.09	0.06
NC_007311:53351411..53352060	12,364.00	11,511.73	11,727.87	10,866.29	11,058.61	-1.10	0.04
NC_007307:73074291..73074392	4,053.00	4,438.08	3,854.86	3,340.09	3,304.91	-1.10	0.15
NC_007313:32348864..32349096	1,732.00	1,871.88	1,582.05	1,395.50	1,486.24	-1.11	0.13
NC_007309:6114394..6114500	1,143.00	1,323.46	1,023.17	887.69	949.26	-1.12	0.18
NC_007306:7462523..7462666	1,654.00	1,506.91	1,524.85	1,421.06	1,424.41	-1.13	0.04
NC_007331:23718929..23719136	3,832.00	4,365.40	3,566.02	3,258.59	2,767.53	-1.13	0.21
NC_007305:83147266..83147386	550.00	468.12	379.22	461.01	784.30	-1.13	0.31
NC_007316:63036964..63037263	1,029.00	1,129.78	920.82	778.78	873.43	-1.13	0.17
NC_007311:75427928..75428373	1,009.00	1,113.27	879.37	783.18	808.92	-1.15	0.17
NC_007303:33142379..33144389	1,971.00	1,789.25	1,767.13	1,677.05	1,620.28	-1.15	0.05
NC_007317:56136529..56138381	1,476.00	1,249.19	1,342.27	1,302.73	1,226.77	-1.15	0.05
NC_007299:147712363..147712852	1,209.00	1,070.51	1,095.48	1,006.67	992.85	-1.16	0.06
NC_007317:19239739..19240424	1,697.00	1,493.56	1,472.99	1,505.63	1,371.39	-1.16	0.05
NC_007317:19239739..19240425	1,697.00	1,493.56	1,472.99	1,505.63	1,371.39	-1.16	0.05
NC_007316:51088141..51088324	1,206.00	1,031.37	953.22	1,084.26	1,067.98	-1.17	0.07
NC_007304:92072164..92072287	13,885.00	12,677.62	11,540.49	11,234.38	12,042.98	-1.17	0.06
NC_007306:7464281..7464409	1,744.00	1,608.55	1,502.42	1,377.96	1,449.12	-1.18	0.08

Continued on next page...

Table 1 – continued from previous page

Location	TCT1	TCT3	TCT4	TCT5	TCT6	Mean ratio	STD
NC_007309:101739696..101741734	1,179.00	1,306.53	1,074.25	908.38	827.77	-1.18	0.23
NC_007330:27687384..27687475	8,053.00	7,231.94	7,679.54	7,131.76	5,613.57	-1.18	0.17
NC_007326:40637825..40638584	17,690.00	19,319.16	17,176.46	13,114.28	12,312.40	-1.18	0.25
NC_007324:28601855..28602124	868.00	600.48	986.88	515.62	1,191.78	-1.18	0.45
NC_007303:110659411..110659676	1,665.00	1,432.10	1,489.09	1,303.58	1,409.09	-1.18	0.07
NC_007303:10853525..10853783	1,666.00	1,432.64	1,489.38	1,304.00	1,409.39	-1.19	0.07
NC_007316:13527593..13527996	2,097.00	2,272.96	1,937.71	1,546.01	1,515.12	-1.19	0.22
NC_007317:13823479..13823579	2,859.00	2,673.03	3,196.83	2,255.51	1,851.41	-1.19	0.28
NC_007302:117606726..117607727	1,180.00	973.47	1,029.67	1,050.85	903.36	-1.20	0.08
NC_007301:34816976..34820789	455.00	373.00	394.12	379.38	359.83	-1.21	0.05
NC_007304:91878197..91878329	11,025.00	9,369.69	9,188.00	8,931.05	8,825.84	-1.22	0.03
NC_007300:133635815..133635925	911.00	816.04	789.39	724.72	682.77	-1.22	0.10
NC_007305:61220933..61221105	1,230.00	974.49	1,122.70	1,068.22	893.13	-1.22	0.12
NC_007306:116404519..116404590	972.00	814.85	792.70	812.57	757.68	-1.22	0.04
NC_007318:35788421..35788535	481.00	628.27	339.38	415.16	306.96	-1.23	0.35
NC_007314:3530462..3532063	355.00	302.25	296.95	278.67	278.15	-1.23	0.05
NC_007301:58304390..58304586	441.00	369.65	337.40	373.45	349.47	-1.24	0.06
NC_007316:55360156..55360344	3,020.00	3,202.18	2,391.83	2,122.88	2,205.82	-1.25	0.21
NC_007319:67026331..67026599	732.00	625.91	636.01	532.86	554.22	-1.25	0.11
NC_007300:123583822..123584569	1,761.00	1,911.32	1,966.43	1,164.76	1,042.87	-1.25	0.41
NC_007317:40681636..40681763	1,522.00	1,733.56	1,326.15	916.54	1,131.00	-1.26	0.33
NC_007300:133638103..133638253	675.00	570.15	573.12	497.76	506.13	-1.26	0.10
NC_007324:8762045..8762122	1,205.00	884.44	990.77	1,002.33	946.82	-1.26	0.07
NC_007301:115599274..115600894	342.00	295.37	287.34	283.19	226.58	-1.27	0.16
NC_007320:22084619..22084924	394.00	307.86	333.90	305.89	293.21	-1.27	0.07
NC_007330:51464681..51464818	539.00	454.83	409.50	449.04	386.59	-1.27	0.10
NC_007306:7457578..7457703	975.00	804.60	712.61	772.10	766.17	-1.28	0.07
NC_007324:28486796..28487065	1,053.00	1,174.24	760.55	700.02	781.01	-1.28	0.27
NC_007305:7352056..7353048	363.00	296.01	298.34	258.47	281.71	-1.28	0.09
NC_007301:21666437..21667183	736.00	597.35	511.78	571.14	592.08	-1.30	0.09
NC_007324:7392287..7392884	615.00	484.15	530.24	421.93	465.60	-1.30	0.12
NC_007324:26284567..26284848	569.00	387.39	440.80	429.48	499.58	-1.31	0.14
NC_007317:57543008..57543145	1,190.00	965.89	943.78	873.80	858.05	-1.31	0.08
NC_007309:108287579..108289153	124.00	94.21	99.34	97.25	87.08	-1.32	0.08
NC_007319:21864371..21864583	315.00	262.08	234.89	239.45	222.14	-1.32	0.09
NC_007331:23698693..23699179	1,044.00	909.07	762.65	786.85	728.78	-1.32	0.12
NC_007326:27949549..27949668	460.00	374.38	347.27	348.83	321.25	-1.33	0.08
NC_007304:91878469..91878552	17,086.00	15,303.76	13,503.46	11,299.99	11,661.91	-1.34	0.18
NC_007316:50190162..50190726	532.00	620.36	438.33	306.11	342.43	-1.34	0.39
NC_007311:16214633..16216017	156.00	123.35	124.40	116.91	102.31	-1.34	0.13
NC_007317:63660341..63660511	230.00	171.57	169.99	180.82	160.59	-1.35	0.07
NC_007330:10233095..10234693	248.00	205.24	170.95	172.43	190.47	-1.35	0.12
NC_007317:26821480..26821753	2,590.00	2,233.17	1,757.63	1,907.11	1,797.46	-1.36	0.14
NC_007304:60945227..60945342	657.00	514.67	565.05	435.47	437.27	-1.36	0.17
NC_007306:63234571..63234874	220.00	169.56	155.83	163.90	156.67	-1.36	0.05
NC_007300:110796158..110796277	282.00	229.47	208.05	199.53	192.91	-1.36	0.10
NC_007311:75421837..75422027	309.00	244.97	204.75	238.89	215.93	-1.37	0.12
NC_007317:49747503..49748240	296.00	241.32	220.76	198.62	202.22	-1.38	0.12
NC_007309:26872631..26873023	374.00	298.29	277.94	225.73	295.03	-1.38	0.19
NC_007311:75524163..75524302	4,146.00	3,544.33	3,785.97	2,563.29	2,500.30	-1.39	0.29
NC_007324:28599996..28600103	1,754.00	1,375.16	1,449.63	1,032.17	1,291.58	-1.39	0.22
NC_007325:3917275..3917437	377.00	297.56	306.61	243.40	250.46	-1.39	0.16

Continued on next page...

Table 1 – continued from previous page

Location	TCT1	TCT3	TCT4	TCT5	TCT6	Mean ratio	STD
NC_007313:77468560..77468938	196.00	150.40	136.60	139.75	136.80	-1.39	0.06
NC_007317:41858676..41860232	461.00	371.15	346.49	295.22	318.53	-1.40	0.14
NC_007324:7393442..7393534	141.00	96.55	206.63	92.95	73.26	-1.40	0.52
NC_007304:24031473..24031956	213.00	156.17	158.17	144.39	150.60	-1.40	0.06
NC_007330:42120918..42121248	41,170.00	29,854.93	32,682.35	28,900.08	26,673.05	-1.40	0.12
NC_007314:49098789..49099628	297.00	244.45	222.23	194.92	186.53	-1.42	0.17
NC_007330:27564013..27564186	457.00	380.76	325.40	299.56	296.45	-1.42	0.16
NC_007305:15487342..15488216	288.00	231.40	240.77	185.41	171.62	-1.42	0.23
NC_007324:28658889..28659158	1,168.00	1,002.06	948.24	465.79	1,478.05	-1.42	0.75
NC_007299:118457405..118458919	156.00	111.11	93.20	129.82	109.68	-1.43	0.19
NC_007324:7744634..7744748	1,101.00	880.69	842.30	707.12	688.05	-1.43	0.18
NC_007330:47476548..47477325	766.00	607.66	546.79	494.37	498.66	-1.44	0.14
NC_007305:20628047..20628096	332.00	274.72	210.66	216.30	231.97	-1.44	0.16
NC_007315:72053944..72054988	240.00	165.86	190.07	166.88	149.01	-1.44	0.14
NC_007300:68846474..68846566	178.00	123.71	131.13	114.02	126.50	-1.44	0.09
NC_007304:109691754..109691834	198.00	146.30	144.41	140.38	121.36	-1.44	0.13
NC_007309:75503085..75503705	236.00	158.54	188.17	162.55	146.98	-1.45	0.15
NC_007324:18406791..18407373	123.00	83.42	89.00	84.98	82.00	-1.45	0.05
NC_007330:18926857..18927176	105.00	72.97	80.54	67.08	69.82	-1.45	0.11
NC_007318:970108..970351	143.00	104.18	106.55	84.56	97.72	-1.47	0.16
NC_007330:38909972..38910729	96.00	69.28	71.02	58.83	62.88	-1.47	0.13
NC_007306:62532329..62532475	215.00	161.14	159.53	140.24	127.86	-1.47	0.17
NC_007307:33351466..33352333	143.00	105.65	93.60	93.23	95.42	-1.48	0.08
NC_007326:28030704..28030968	345.00	261.12	246.06	220.90	211.76	-1.48	0.14
NC_007316:58025516..58025748	179.00	126.82	119.86	118.75	118.37	-1.48	0.05
NC_007311:55580745..55580800	931.00	1,081.11	706.78	535.25	452.91	-1.49	0.52
NC_007326:40127549..40127620	351.00	416.11	243.57	204.97	177.27	-1.49	0.49
NC_007307:64047265..64048032	133.00	94.24	93.19	72.93	100.17	-1.50	0.22
NC_007306:65965508..65965797	114.00	80.60	80.44	67.54	76.29	-1.50	0.13
NC_007303:104172406..104173395	246.00	193.83	174.92	141.00	154.12	-1.50	0.21
NC_007317:27333757..27333889	172.00	108.19	126.70	105.71	118.56	-1.51	0.12
NC_007314:26417920..26418134	181.00	128.11	113.86	119.08	117.96	-1.51	0.07
NC_007309:74324865..74325412	244.00	174.77	163.44	155.49	152.50	-1.51	0.09
NC_007300:132462131..132463009	584.00	485.20	474.00	311.17	332.14	-1.52	0.35
NC_007304:91927492..91927644	4,358.00	2,757.55	2,669.01	2,877.16	3,187.34	-1.52	0.11
NC_007331:30709743..30709848	170.00	119.25	125.31	108.65	96.48	-1.53	0.18
NC_007313:27675902..27676107	189.00	145.36	139.43	113.88	104.75	-1.53	0.24
NC_007317:41575965..41576329	131.00	92.68	93.55	79.99	76.81	-1.54	0.16
NC_007301:83613674..83614055	384.00	301.55	270.43	234.99	209.78	-1.54	0.24
NC_007303:51565883..51566044	383.00	311.88	214.17	279.75	214.23	-1.54	0.29
NC_007300:135874077..135874437	132.00	93.02	88.40	73.41	88.74	-1.55	0.17
NC_007304:91877902..91878056	7,770.00	4,307.65	5,473.95	5,367.89	4,858.02	-1.57	0.18
NC_007301:33193248..33193454	267.00	184.33	205.34	137.62	165.72	-1.58	0.27
NC_007307:29721888..29722067	93.00	61.04	56.21	55.74	63.37	-1.58	0.10
NC_007311:58097662..58097865	538.00	348.48	359.71	263.58	420.54	-1.59	0.32
NC_007316:62648658..62648776	115.00	80.04	72.82	66.69	70.60	-1.59	0.12
NC_007317:15607468..15607543	113.00	75.83	78.52	68.07	62.79	-1.60	0.16
NC_007305:10886973..10887133	190.00	135.27	133.54	107.93	105.38	-1.60	0.21
NC_007316:63351125..63351422	165.00	124.77	86.01	96.80	113.85	-1.60	0.27
NC_007305:1551817..1551909	207.00	151.42	120.50	129.36	119.53	-1.60	0.17
NC_007317:15405863..15406012	113.00	74.94	78.04	67.60	62.54	-1.61	0.16
NC_007311:66567161..66567426	377.00	261.89	289.67	192.85	210.22	-1.62	0.30

Continued on next page...

Table 1 – continued from previous page

Location	TCT1	TCT3	TCT4	TCT5	TCT6	Mean ratio	STD
NC_007300:132456936..132457094	290.00	237.04	222.67	130.69	165.84	-1.62	0.46
NC_007320:54713489..54715855	94.00	59.20	58.78	55.61	58.04	-1.62	0.05
NC_007309:102777022..102777265	116.00	74.45	77.96	63.31	71.44	-1.63	0.15
NC_007324:49880061..49881829	391.00	301.60	214.06	210.58	254.48	-1.63	0.26
NC_007309:101677328..101677426	279.00	189.74	153.15	163.90	179.44	-1.64	0.16
NC_007305:13746108..13746359	138.00	84.06	94.94	72.57	86.64	-1.65	0.19
NC_007304:119887661..119888303	324.00	245.07	221.75	159.02	182.79	-1.65	0.32
NC_007324:7382870..7383039	463.00	325.34	334.93	254.05	235.26	-1.65	0.29
NC_007305:18906401..18907160	108.00	66.05	70.84	55.11	72.04	-1.65	0.21
NC_007317:19248053..19248216	247.00	196.56	171.53	129.27	119.05	-1.67	0.39
NC_007314:29689118..29689484	357.00	214.98	176.36	213.57	261.46	-1.68	0.27
NC_007304:101901856..101902116	62.00	38.13	37.43	35.89	36.01	-1.68	0.05
NC_007300:120326690..120326852	76.00	44.87	44.16	48.70	42.97	-1.69	0.09
NC_007311:61964452..61964732	121.00	79.15	62.55	88.13	62.48	-1.69	0.29
NC_007316:56018540..56018712	70.00	44.45	44.89	43.42	32.77	-1.72	0.28
NC_007303:61326976..61327172	94.00	50.74	54.40	62.68	51.77	-1.72	0.16
NC_007304:95164618..95165159	123.00	70.20	77.17	77.71	62.32	-1.73	0.18
NC_007299:2920763..2920913	142.00	85.45	79.12	88.54	76.95	-1.73	0.11
NC_007317:35415437..35416125	67.00	40.38	37.25	38.79	38.91	-1.73	0.06
NC_007303:60756403..60756593	86.00	47.51	49.81	52.04	49.92	-1.73	0.06
NC_007316:49435584..49435748	57.00	35.22	29.42	33.21	34.38	-1.73	0.14
NC_007325:44763377..44763522	76.00	45.28	48.49	47.43	36.24	-1.74	0.24
NC_007306:102114798..102115230	108.00	68.97	63.17	61.22	56.33	-1.74	0.15
NC_007303:29751051..29751252	268.00	177.51	166.93	139.33	134.07	-1.76	0.24
NC_007316:62241751..62242352	231.00	178.52	176.69	100.95	95.76	-1.83	0.61
NC_007314:24108039..24108402	87.00	44.34	54.94	41.64	50.58	-1.84	0.23
NC_007305:13235263..13235413	47.00	25.12	25.91	23.86	27.04	-1.85	0.10
NC_007303:94565462..94565752	70.00	28.61	40.18	41.29	45.26	-1.86	0.40
NC_007310:73978232..73978477	74.00	32.47	45.06	46.41	38.41	-1.86	0.32
NC_007306:79382442..79382839	65.00	38.51	35.19	29.47	37.67	-1.87	0.24
NC_007303:22139021..22139203	47.00	26.64	23.00	25.69	25.69	-1.87	0.12
NC_007305:11050059..11050439	172.00	121.22	109.44	77.74	74.66	-1.88	0.45
NC_007303:113823888..113824044	50.00	26.13	29.54	24.13	26.99	-1.88	0.16
NC_007316:62648851..62649001	73.00	38.83	38.25	33.01	47.07	-1.89	0.27
NC_007330:42628346..42628488	100.00	66.09	56.82	39.15	55.72	-1.91	0.45
NC_007306:79035541..79036147	84.00	55.27	54.38	36.42	36.00	-1.93	0.45
NC_007305:16017612..16017814	117.00	79.56	79.74	53.36	45.21	-1.93	0.56
NC_007328:39688996..39689563	48.00	24.26	25.16	24.21	25.83	-1.93	0.06
NC_007315:66089655..66090233	103.00	67.46	56.69	37.43	60.55	-1.95	0.55
NC_007304:92071945..92072071	2,337.00	1,248.82	1,336.83	1,045.20	1,195.91	-1.95	0.21
NC_007304:92071948..92072071	2,337.00	1,248.82	1,336.83	1,045.20	1,195.91	-1.95	0.21
NC_007301:110481878..110482183	250.00	170.19	186.66	117.07	86.83	-1.96	0.71
NC_007302:117339475..117339528	130.00	91.25	77.18	59.59	50.48	-1.97	0.51
NC_007316:50122827..50123147	105.00	68.20	49.42	45.59	55.01	-1.97	0.33
NC_007304:92060262..92060421	1,204.00	680.47	632.49	473.57	723.29	-1.97	0.39
NC_007305:17042479..17042601	67.00	38.52	38.28	34.21	26.13	-2.00	0.39
NC_007302:79534027..79534155	65.00	35.67	30.96	34.32	29.11	-2.01	0.19
NC_007303:61393646..61393792	44.00	22.89	23.88	21.26	19.87	-2.01	0.16
NC_007301:62881085..62881730	217.00	160.67	125.78	82.60	89.43	-2.03	0.60
NC_007306:83588959..83589119	60.00	32.09	35.42	27.74	24.50	-2.04	0.33
NC_007299:147738036..147738298	214.00	156.50	120.92	68.25	111.09	-2.05	0.76
NC_007300:122840977..122841164	155.00	102.96	109.19	58.47	58.12	-2.06	0.69

Continued on next page...

Table 1 – continued from previous page

Location	TCT1	TCT3	TCT4	TCT5	TCT6	Mean ratio	STD
NC_007306:61991330..61991606	81.00	44.65	44.76	34.29	35.13	-2.07	0.30
NC_007320:51026105..51026364	326.00	246.68	190.89	108.74	133.76	-2.12	0.75
NC_007319:56731170..56731399	45.00	16.10	23.00	23.01	24.91	-2.13	0.45
NC_007317:27313343..27313418	56.00	32.60	29.48	20.75	24.04	-2.16	0.44
NC_007300:68805338..68805502	175.00	90.35	66.52	87.61	68.22	-2.28	0.37
NC_007330:37963995..37964186	75.00	30.46	42.78	24.13	41.18	-2.29	0.63
NC_007324:40257493..40258285	114.00	65.70	54.26	42.39	40.97	-2.33	0.50
NC_007331:81985779..81985892	1,516.00	1,852.30	1,271.54	533.80	328.28	-2.37	1.74
NC_007301:117813983..117814902	126.00	58.42	61.90	47.23	48.26	-2.37	0.32
NC_007326:39519693..39519962	56.00	30.32	23.53	17.35	27.40	-2.37	0.61
NC_007317:39994002..39994729	38.00	18.58	15.72	11.73	19.51	-2.41	0.59
NC_007317:55010104..55010534	153.00	91.10	90.44	39.13	63.25	-2.43	1.05
NC_007313:81452899..81453175	67.00	37.46	33.18	18.20	29.69	-2.44	0.85
NC_007301:21518293..21518356	128.00	95.84	61.69	70.05	28.18	-2.44	1.43
NC_007302:110832307..110832520	95.00	59.69	46.09	25.46	37.72	-2.48	0.92
NC_007319:68155488..68155688	95.00	38.97	39.99	30.56	47.69	-2.48	0.46
NC_007299:141696679..141696789	58.00	23.87	28.65	23.06	19.65	-2.48	0.38
NC_007326:2004184..2004363	1,416.00	782.94	714.62	388.35	494.19	-2.58	0.85
NC_007313:4475855..4476238	44.00	21.98	23.98	12.86	13.77	-2.61	0.81
NC_007317:34142166..34142245	190.00	112.69	70.16	54.83	72.02	-2.62	0.73
NC_007318:14681093..14681193	24.00	9.74	7.77	9.03	10.35	-2.63	0.33
NC_007326:40635163..40635246	257.00	199.33	114.96	92.14	56.70	-2.71	1.36
NC_007306:41258116..41258492	27.00	9.28	8.58	8.89	10.04	-2.95	0.20
NC_007309:99280747..99280828	129.00	77.61	51.66	37.80	26.62	-3.10	1.36
NC_007314:48127959..48128171	87.00	56.95	50.27	15.34	20.54	-3.29	2.01
NC_007313:28099681..28099742	46.00	20.48	11.03	15.28	11.00	-3.40	0.95
NC_007306:116000559..116000837	36.00	14.79	18.06	6.65	8.62	-3.50	1.58
NC_007316:52305545..52305888	112.00	78.19	57.87	17.26	25.50	-3.56	2.34
NC_007319:24588317..24588737	21.00	7.87	6.15	4.81	4.87	-3.69	0.81
NC_007330:27770633..27770724	849.00	740.21	177.11	159.39	219.00	-3.79	1.86
NC_007330:27773099..27773237	791.00	634.17	112.05	144.21	385.32	-3.96	2.76
NC_007304:118134893..118135229	34.00	11.41	12.18	5.07	9.37	-4.03	1.82
NC_007326:40118446..40118653	28.00	7.71	11.96	4.10	6.73	-4.24	1.89
NC_007311:74138461..74138524	23.00	9.27	9.20	3.05	4.93	-4.30	2.39
NC_007326:27879802..27879832	13.00	4.11	1.84	2.19	2.18	-5.53	1.66
NC_007311:75437204..75437332	12.00	1.13	2.64	3.28	2.77	-5.79	3.24
NC_007299:157629052..157629320	52.00	23.86	12.88	2.69	10.68	-7.60	7.90
NC_007316:55891981..55892017	181.00	103.11	27.77	32.12	5.58	-11.59	14.05
NC_007326:1034735..1034967	13.00	1.19	2.04	0.51	1.73	-12.58	8.82

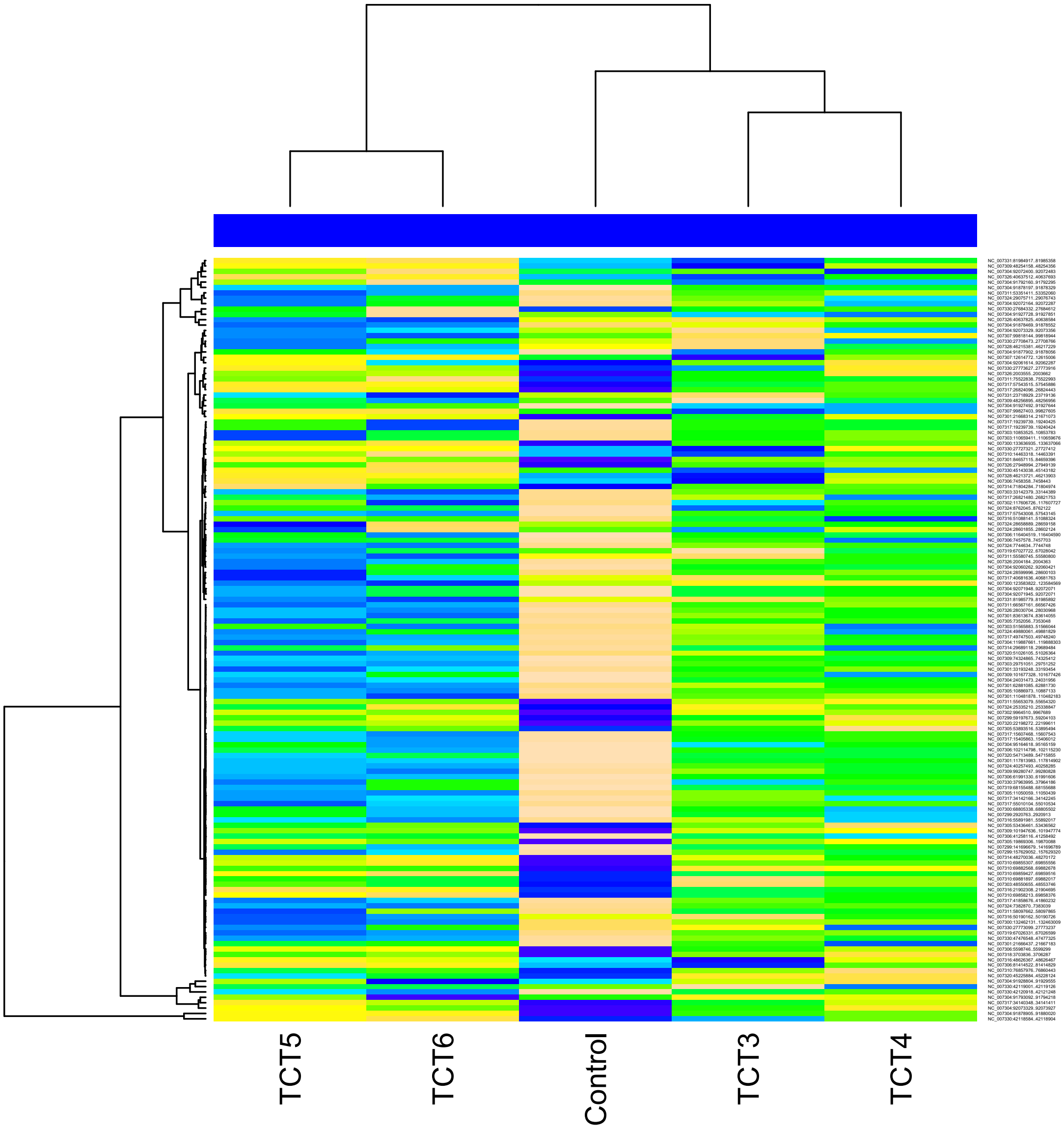

Figure 1: **Heatmap of exon inclusion changes relative to gene expression changes.** Heat map along the dendrograms of exon inclusions that have been found significantly different from Control. Blue color indicates exons relatively underexpressed and sandstone color indicates exons relatively overexpressed compared to the gene expression level.
