# Supplementary material for: Accurate Diagnostics for Bovine tuberculosis Based on High-Throughput Sequencing
Source: PLoS One. 2012 Nov 30;7(11):e50147. doi: 10.1371/journal.pone.0050147 (PMC3511461; doi:10.1371/journal.pone.0050147)
Supplement: Supporting Information S6 — Reference sequences used. (PDF) [file pone.0050147.s006.pdf]

Supporting Information S6

Accurate diagnostics for *Bovine tuberculosis* based on high-throughput sequencing

Alexander Churbanov and Brook Milligan

Reference sequences used

All experiments were conducted with the Btau 4.0 based reference genome sequences and annotations from NCBI GenBank `http://www.ncbi.nlm.nih.gov/`.

```
>gi|194719325|ref|NC_007299.3|NC_007299 Bos taurus chromosome 1
>gi|194719396|ref|NC_007300.3|NC_007300 Bos taurus chromosome 2
>gi|194719407|ref|NC_007301.3|NC_007301 Bos taurus chromosome 3
>gi|194719408|ref|NC_007302.3|NC_007302 Bos taurus chromosome 4
>gi|194719431|ref|NC_007303.3|NC_007303 Bos taurus chromosome 5
>gi|194719536|ref|NC_007304.3|NC_007304 Bos taurus chromosome 6
>gi|194719537|ref|NC_007305.3|NC_007305 Bos taurus chromosome 7
>gi|194719538|ref|NC_007306.3|NC_007306 Bos taurus chromosome 8
>gi|194719539|ref|NC_007307.3|NC_007307 Bos taurus chromosome 9
>gi|194719326|ref|NC_007308.3|NC_007308 Bos taurus chromosome 10
>gi|194719387|ref|NC_007309.3|NC_007309 Bos taurus chromosome 11
>gi|194719388|ref|NC_007310.3|NC_007310 Bos taurus chromosome 12
>gi|194719389|ref|NC_007311.3|NC_007311 Bos taurus chromosome 13
>gi|194719390|ref|NC_007312.3|NC_007312 Bos taurus chromosome 14
>gi|194719391|ref|NC_007313.3|NC_007313 Bos taurus chromosome 15
>gi|194719392|ref|NC_007314.3|NC_007314 Bos taurus chromosome 16
>gi|194719393|ref|NC_007315.3|NC_007315 Bos taurus chromosome 17
>gi|194719394|ref|NC_007316.3|NC_007316 Bos taurus chromosome 18
>gi|194719395|ref|NC_007317.3|NC_007317 Bos taurus chromosome 19
>gi|194719397|ref|NC_007318.3|NC_007318 Bos taurus chromosome 20
>gi|194719398|ref|NC_007319.3|NC_007319 Bos taurus chromosome 21
>gi|194719399|ref|NC_007320.3|NC_007320 Bos taurus chromosome 22
>gi|194719400|ref|NC_007324.3|NC_007324 Bos taurus chromosome 23
>gi|194719401|ref|NC_007325.3|NC_007325 Bos taurus chromosome 24
>gi|194719402|ref|NC_007326.3|NC_007326 Bos taurus chromosome 25
>gi|194719403|ref|NC_007327.3|NC_007327 Bos taurus chromosome 26
```

```
>gi|194719404|ref|NC_007328.3|NC_007328 Bos taurus chromosome 27
>gi|194719405|ref|NC_007329.3|NC_007329 Bos taurus chromosome 28
>gi|194719406|ref|NC_007330.3|NC_007330 Bos taurus chromosome 29
>gi|194719540|ref|NC_007331.3|NC_007331 Bos taurus chromosome X
```
